# Chemokines in Non-alcoholic Fatty Liver Disease: A Systematic Review and Network Meta-Analysis

**DOI:** 10.3389/fimmu.2020.01802

**Published:** 2020-09-18

**Authors:** Xiongfeng Pan, Atipatsa Chiwanda Kaminga, Aizhong Liu, Shi Wu Wen, Jihua Chen, Jiayou Luo

**Affiliations:** ^1^Xiangya School of Public Health, Central South University, Changsha, China; ^2^Department of Mathematics and Statistics, Mzuzu University, Mzuzu, Malawi; ^3^Department of Obstetrics and Gynaecology, University of Ottawa, Ottawa, ON, Canada; ^4^Ottawa Hospital Research Institute, Ottawa, ON, Canada; ^5^Department of Food Science and Biotechnol, Kagoshima University, Kagoshima, Japan

**Keywords:** chemokines, non-alcoholic fatty liver disease, non-alcoholic steatohepatitis, systematic review, network meta-analysis

## Abstract

**Background:** Previous results on the relationship between non-alcoholic fatty liver disease (NAFLD) and chemokine concentrations were inconsistent. The purpose of this network meta-analysis was to evaluate the link between chemokine system and NAFLD.

**Methods:** Relevant data, published not later than June 31, 2019, were searched in the databases of PubMed, Embase, Cochrane Library, and Web of Science. A network meta-analysis was used to rank the chemokines by surface under the cumulative ranking (SUCRA) probabilities. In addition, standardized mean differences (SMDs) with 95% confidence intervals (CIs) were calculated as group differences in the chemokine concentrations.

**Results:** The search in the databases identified 46 relevant studies that investigated the relationship between 15 different chemokines and NAFLD using 4,753 patients and 4,059 controls. Results from the network meta-analysis showed that the concentrations of CCL2 and CXCL8 in the non-alcoholic fatty liver (NAFL) group was significantly higher than that in the control group (SMDs of 1.51 and 1.95, respectively), and the concentrations of CCL3, CCL4, CCL20, CXCL8, and CXCL10 in the non-alcoholic steatohepatitis (NASH) group was significantly higher than that in the control group (SMDs of 0.90, 2.05, 2.16, 0.91, and 1.46, respectively). SUCRA probabilities showed that CXCL8 had the highest rank in NAFL for all chemokines and CCL20 had the highest rank in NASH for all chemokines.

**Conclusion:** Elevated concentrations of CCL2, CCL4, CCL20, CXCL8, and CXCL10 may be associated with NAFL or NASH. In this regard, more population-based studies are needed to ascertain this hypothesis.

**Systematic Review Registration:** PROSPERO: CRD42020139373.

## Background

Non-alcoholic fatty liver disease (NAFLD) is currently one of the major public health problems worldwide, and its prevalence has been rising sharply following changes in lifestyle due to improvement in the living conditions of people (1, 2). NAFLD is closely related to liver insufficiency and can cause a variety of serious complications. For example, NAFLD can cause cardiometabolic complications, metabolic syndrome, and type 2 diabetes mellitus. In addition, it increases the risk of advanced cirrhosis, liver failure, liver transplantation, and hepatocellular carcinoma (1, 3). However, despite the urgent need for medical treatment of NAFLD, specific therapeutic drugs for NAFLD are not available thus far, which has spurred multidisciplinary research to better understand the potential complex causes of the NAFLD (4).

In this regard, NAFLD is a common multisystem chronic liver progressive disease that interacts with the regulation of multiple pro-inflammatory, endocrine, and metabolic pathways and affects extrahepatic organ systems (5). Specifically, there has been laboratory evidence suggesting that chemokines influenced pro-inflammation and pro-oxidative mechanisms played a crucial role in the possible pathogenetic process of NAFLD (6). Chemokines are small and highly conserved protein families divided into four subfamilies (C, CC, CXC, and CX3C). They have been shown to be involved in various biological processes of NAFLD pathophysiology, including chemotaxis, which not only regulates the migration and activities of immune cell to the site of inflammation but also mediates the secretion and production of inflammatory mediators and activates the lymphoid tissue maturation (7).

Precisely, chemokine activation in NAFLD induces a variety of additional cellular and tissue responses such as the modulation of hepatocyte proliferation, activation, extracellular matrix remodeling, angiogenesis, and direct activation of stellate cells (8). Also, previous studies have shown that chemokines promote the development of obesity by recruiting pro-inflammatory monocytes into hypertrophic fat tissue, which suggests that chemokines may be involved in energy metabolism, lipid metabolic disorders, and obesity (9).

Given the foregoing evidence, it can be deduced that chemokines and their receptors may play a vital role in the development of NAFLD and should be considered as potential therapeutic targets for NAFLD. Nevertheless, there are currently about 50 chemokines acting on 23 discrete receptors, and there has been a disparity in the results of previous studies examining the relationship between chemokines and NAFLD, perhaps due to methodological differences in conducting those studies (6). Therefore, it is currently unclear as to which chemokines may be involved in the pathogenesis of NAFLD. Network meta-analysis can quantitatively analyze the contradictory results on the relationship between chemokines and NAFLD to better understand which chemokines may play an important role in the development of NAFLD. Therefore, this study conducted a systematic review and network meta-analysis of some previous studies on the relationship between chemokines and NAFLD. The aim was to combine direct and indirect evidence on the connection between multiple chemokines and NAFLD and to accurately assess the nature and extent of this connection. Additionally, the relationship between the chemokines and histological severity of NAFLD such as non-alcoholic steatohepatitis (NASH) was assessed (10). Clarification of the magnitude of the potential effect of chemokines on the progression of NAFLD may have implications for preventing the pathophysiological transversion of non-alcoholic fatty liver (NAFL) to NASH.

## Methods

### Search Strategy and Selection Criteria

This study was based on the Cochrane Handbook and Preferred Reporting Items for Systematic Reviews and Meta-Analyses (PRISMA) guidelines (11) and registered in the International Prospective Register of Systemic Reviews (PROSPERO). The registration number is CRD42020139373.

Relevant studies were selected in accordance with the purpose of this study by two authors (AK and XP). Accordingly, the following English databases were searched for relevant studies published not later than June 31, 2019: EMBASE, Web of Science, Cochrane Library, and PubMed. Based on the nomenclature of chemokines, each database's search algorithm was designed and adjusted with respect to a combination of related terms by experienced librarians [complete search strategy is in the Appendix 1 (Supplementary Material)]. Studies were selected if they met the following inclusion criteria: the studies were case–control; reported a method for diagnosing NAFLD; and provided the mean and standard deviation (SD) of chemokine concentration, or these could be obtained from respective authors upon request. Case reports or comments, studies that reported NAFLD in combination with other diseases, and studies that did not examine humans were excluded.

### Data Extraction

Two authors (JC and AK) independently assessed all the searched studies for eligibility and extracted data, which included (1) the first author's name and publication year; (2) the laboratory characteristics of the participants such as alanine aminotransferase (ALT), glutamyl transpeptidase (GGT), aspartate aminotransferase (AST), high-density lipoprotein (HDL), triglycerides (TG), low-density lipoprotein (LDL), and insulin resistance index (HOMA-IR); (3) field of study; (4) subject characteristics such as body mass index (BMI), mean age and SD (mean, SD), and gender; (5) chemokine sample test methods and storage temperatures; and (6) sample characteristics such as material of sample, and mean and SD of chemokine concentration, were abstracted. Discrepancies between the two authors were resolved by involving the third author (JL). Finally, the Newcastle–Ottawa Quality Assessment Scale (NOS) was used to assess the quality of the eligible studies, and the risk of bias.

### Statistical Analysis

After all the relevant data were extracted, the transitivity assumption was evaluated before conducting the network meta-analysis. When the random-effects model was satisfied, the network meta-analysis, with consistency assumption, was conducted by the Markov-chain Monte Carlo method in Stata version 15.0, using the network command and self-programmed Stata routines, to compare the effects of multiple-correction chemokines. The first 20,000 iterations were discarded, and additional 50,000 iterations were executed (12, 13). Moreover, vague priors were used in the network analysis, and the average residual deviation was used to estimate the goodness of fit. The consistency between direct evidence and indirect evidence was assessed by the node-split method. The standardized mean differences (SMDs) with 95% confidence interval (CI) were calculated from continuous data in networks (14, 15). The degree of heterogeneity per network was assessed by comparing the size of the network's τ and generating adjusted funnel plots to assess the publication bias (16, 17). Meanwhile, in order to evaluate the robustness of the results obtained from the preliminary model, a sensitivity analysis was performed by excluding one study at a time. For each of the chemokines, the surface under the cumulative ranking curve (SUCRA) was also calculated to rank the chemokines. Thus, chemokines with higher SUCRA values were considered the most potent chemokines (18).

## Results

### Literature Search

The search yielded a total of 2,698 articles, of which 915 were from Web of Science, 956 were from Embase, 736 were from PubMed, and 91 were from Cochrane Library. A full-text review of 399 of the articles identified 46 eligible articles for the network meta-analysis (Appendix 1 in SupplementaryMaterial).

### Characteristics of Eligible Studies

The eligible studies consisted of 15 different chemokines nodes with 92 direct and indirect comparisons involving 4,753 patients and 4,059 controls. Besides, 13 studies selected chemokines from plasma samples, whereas 33 selected chemokines were from serum samples. Also, 11 studies were conducted in the United States of America (USA), 8 in China, 5 in Italy, 3 in Japan, 5 in Turkey, 2 in Belgium, 2 in Iran, 1 in Brazil, 1 in Egypt, 1 in Greece, 1 in India, 1 in Ireland, 1 in Lebanon, 1 in Norway, 1 in Poland, 1 in the United Kingdom (UK), and 1 in Ukraine. The mean (SD) age of patients was 45.91 (12.31), and 42 studies that focused on the BMI of patients reported a mean (SD) BMI of 33.24 (6.86). Furthermore, 28 studies diagnosed NAFLD exclusively using liver biopsy specimen scores, whereas 18 studies diagnosed NAFLD using ultrasonography and magnetic resonance. In addition, 39 studies used ELISA to analyze chemokines, and 27 studies reported temperature at which samples were stored. Moreover, of the 46 eligible studies, 25 investigated NAFL (not developed into NASH), while 21 investigated NASH (Appendix 2 in Supplementary Material). No evidence of statistical heterogeneity was observed, and, in general, a primary inconsistency test was not significant for NAFL (*p* = 0.999) and NASH (*p* = 0.692), suggesting that consistency models could be used for analysis. However, there was a significant difference between direct and indirect coefficients of control-CCL2, CCL3-CXCL10, and CCL4-CXCL10 in the node-splitting test, but no significant differences were observed in other comparisons (Appendix 3 in Supplementary Material). Meanwhile, a summary of the previous knowledge; classification of chemokines including the chemokine subfamily, chemokine common name and systematic name; chemokine corresponding receptor types, different distributions of chemokines, and receptors of chemokines in different cells; and chemokines and their receptors was made in relation to the roles they may play in the NAFL/NASH (Appendix 4 in Supplementary Material).

### Main Outcomes

**Figure 1:**
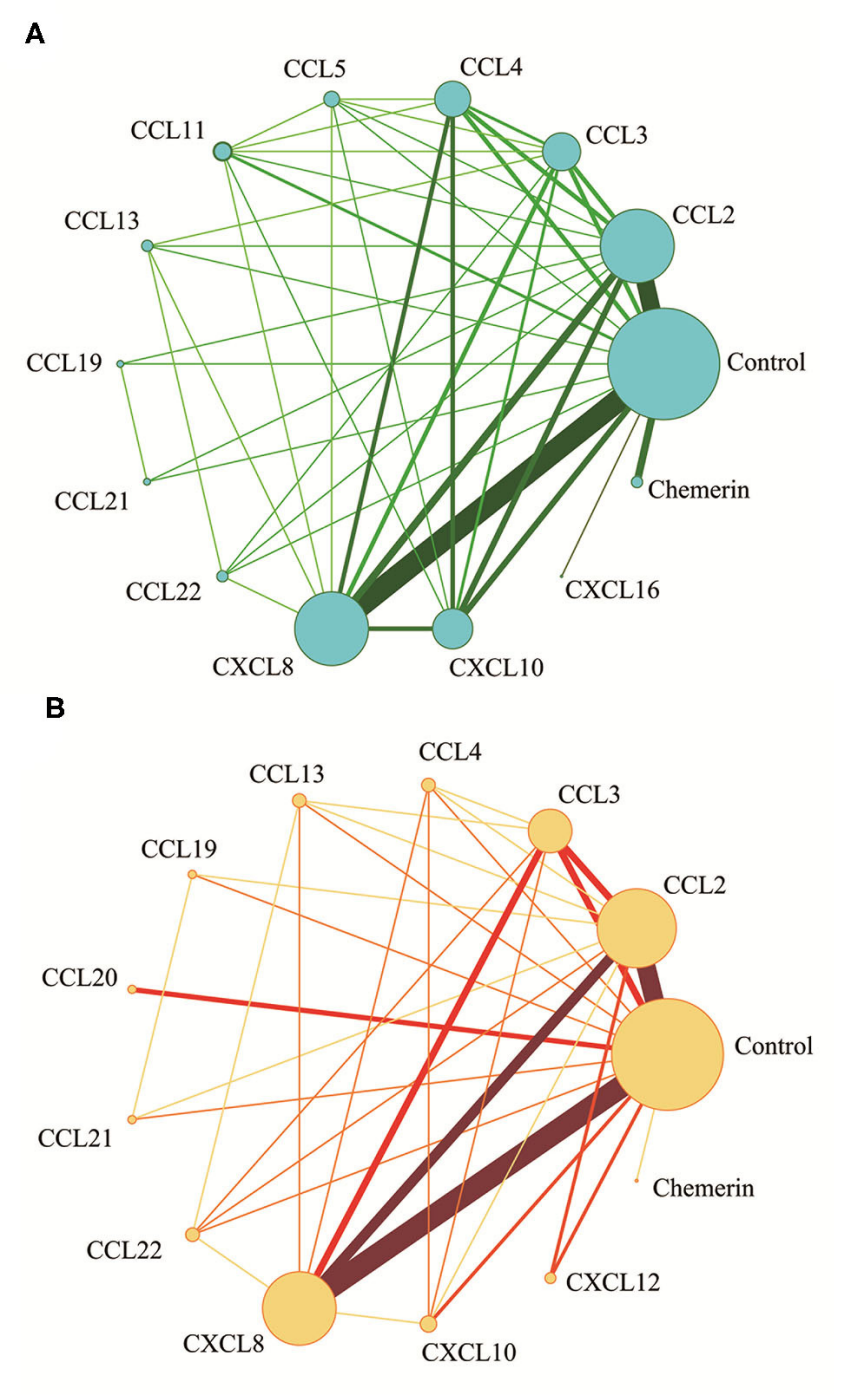
Network meta-analysis of chemokines comparisons for NAFL **(A)** and NASH **(B)**. The width of lines is proportional to the number of studies comparing every pair of chemokines. The size of each circle is proportional to the sample size (i.e., number of participants).

**Figure 2:**
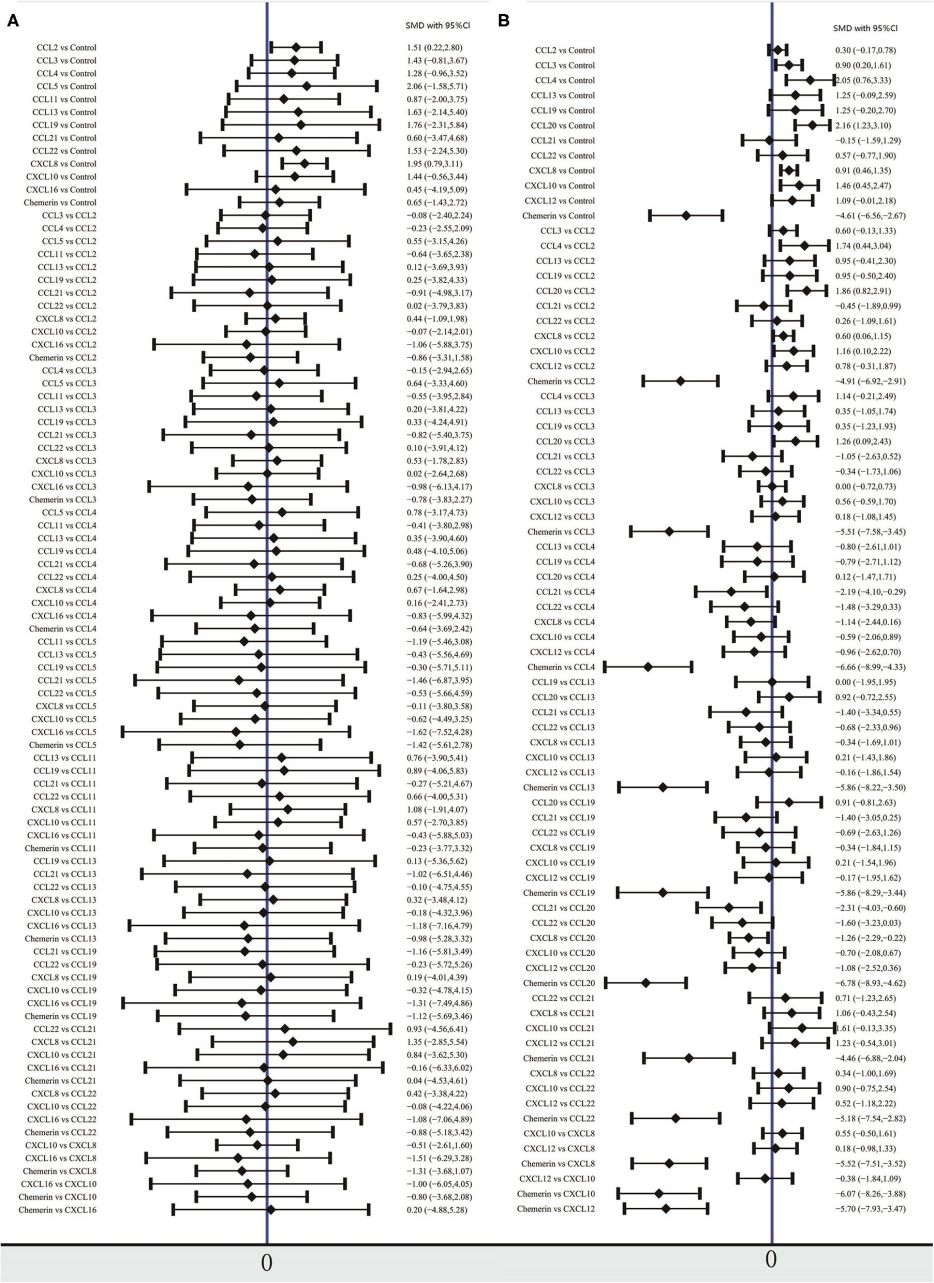
Forest plot of chemokines comparisons for NAFL **(A)** and NASH **(B)**. The summary effect size for each chemokines is denoted by a diamond. It also shows standardized mean differences in effect size and 95% CI. Means more than 0 indicate that the concentration of chemokines in left column was higher than that in the right column. SMD, standardized mean difference; CI, confidence interval.

The network meta-analysis in this study consisted of 14 NAFL nodes and 13 NASH nodes. Each node involved a different chemokine in the patients, or controls (Figure 1). Results of the network meta-analysis showed that the concentrations of CCL2 and CXCL8 in the NAFL group (SMDs of 1.51 and 1.95, respectively) were significantly higher than that in the control group (Table 1A). Similarly, concentrations of CCL3, CCL4, CCL20, CXCL8, and CXCL10 in the NASH group (SMDs of 0.90, 2.05, 2.16, 0.91, and 1.46, respectively) were significantly higher than that in the control group (Table 1B). Figure 2 show the details.

**Table 1A:** Estimated differences of the effect of chemokines on NAFL.

							**NAFL**						
	**CCL2**	**CCL3**	**CCL4**	**CCL5**	**CCL11**	**CCL13**	**CCL19**	**CCL21**	**CCL22**	**CXCL8**	**CXCL10**	**CXCL16**	**Chemerin**
Control	**1.51 (0.22, 2.80)**	1.43 (−0.81, 3.67)	1.28 (−0.96, 3.52)	2.06 (−1.58, 5.71)	0.87 (−2.00, 3.75)	1.63 (−2.14, 5.40)	1.76 (−2.31, 5.84)	0.60 (−3.47, 4.68)	1.53 (−2.24, 5.30)	**1.95 (0.79, 3.11)**	1.44 (−0.56, 3.44)	0.45 (−4.19, 5.09)	0.65 (−1.43, 2.72)
CCL2		−0.08 (−2.40, 2.24)	−0.23 (−2.55, 2.09)	0.55 (−3.15, 4.26)	−0.64 (−3.65, 2.38)	0.12 (−3.69, 3.93)	0.25 (−3.82, 4.33)	−0.91 (−4.98, 3.17)	0.02 (−3.79, 3.83)	0.44 (−1.09, 1.98)	−0.07 (−2.14, 2.01)	−1.06 (−5.88, 3.75)	−0.86 (−3.31, 1.58)
CCL3			−0.15 (−2.94, 2.65)	0.64 (−3.33, 4.60)	−0.55 (−3.95, 2.84)	0.20 (−3.81, 4.22)	0.33 (−4.24, 4.91)	−0.82 (−5.40, 3.75)	0.10 (−3.91, 4.12)	0.53 (−1.78, 2.83)	0.02 (−2.64, 2.68)	−0.98 (−6.13, 4.17)	−0.78 (−3.83, 2.27)
CCL4				0.78 (−3.17, 4.73)	−0.41 (−3.80, 2.98)	0.35 (−3.90, 4.60)	0.48 (−4.10, 5.06)	−0.68 (−5.26, 3.90)	0.25 (−4.00, 4.50)	0.67 (−1.64, 2.98)	0.16 (−2.41, 2.73)	−0.83 (−5.99, 4.32)	−0.64 (−3.69, 2.42)
CCL5					−1.19 (−5.46, 3.08)	−0.43 (−5.56, 4.69)	−0.30 (−5.71, 5.11)	−1.46 (−6.87, 3.95)	−0.53 (−5.66, 4.59)	−0.11 (−3.80, 3.58)	−0.62 (−4.49, 3.25)	−1.62 (−7.52, 4.28)	−1.42 (−5.61, 2.78)
CCL11						0.76 (−3.90, 5.41)	0.89 (−4.06, 5.83)	−0.27 (−5.21, 4.67)	0.66 (−4.00, 5.31)	1.08 (−1.91, 4.07)	0.57 (−2.70, 3.85)	−0.43 (−5.88, 5.03)	−0.23 (−3.77, 3.32)
CCL13							0.13 (−5.36, 5.62)	−1.02 (−6.51, 4.46)	−0.10 (−4.75, 4.55)	0.32 (−3.48, 4.12)	−0.18 (−4.32, 3.96)	−1.18 (−7.16, 4.79)	−0.98 (−5.28, 3.32)
CCL19								−1.16 (−5.81, 3.49)	−0.23 (−5.72, 5.26)	0.19 (−4.01, 4.39)	−0.32 (−4.78, 4.15)	−1.31 (−7.49, 4.86)	−1.12 (−5.69, 3.46)
CCL21									0.93 (−4.56, 6.41)	1.35 (−2.85, 5.54)	0.84 (−3.62, 5.30)	−0.16 (−6.33, 6.02)	0.04 (−4.53, 4.61)
CCL22										0.42 (−3.38, 4.22)	−0.08 (−4.22, 4.06)	−1.08 (−7.06, 4.89)	−0.88 (−5.18, 3.42)
CXCL8											−0.51 (−2.61, 1.60)	−1.51 (−6.29, 3.28)	−1.31 (−3.68, 1.07)
CXCL10												−1.00 (−6.05, 4.05)	−0.80 (−3.68, 2.08)
CXCL16													0.20 (−4.88, 5.28)

*Numbers above the diagonal show standardized mean differences in effect size and 95% CI. Means more than 0 indicate that the chemokines on that row reduce in NAFL than the chemokines on that column. NAFL, non-alcoholic fatty liver. The bold values means the SMD values with statistically significant (p < 0.05)*.

**Table 1B:** Estimated differences of the effect of chemokines on NASH.

						**NASH**						
	**CCL2**	**CCL3**	**CCL4**	**CCL13**	**CCL19**	**CCL20**	**CCL21**	**CCL22**	**CXCL8**	**CXCL10**	**CXCL12**	**Chemerin**
Control	0.30 (−0.17, 0.78)	**0.90 (0.20, 1.61)**	**2.05 (0.76, 3.33)**	1.25 (−0.09, 2.59)	1.25 (−0.20, 2.70)	**2.16 (1.23, 3.10)**	−0.15 (−1.59, 1.29)	0.57 (−0.77, 1.90)	**0.91 (0.46, 1.35)**	**1.46 (0.45, 2.47)**	1.09 (−0.01, 2.18)	**−4.61 (−6.56**, **−2.67)**
CCL2		0.60 (−0.13, 1.33)	**1.74 (0.44, 3.04)**	0.95 (−0.41, 2.30)	0.95 (−0.50, 2.40)	**1.86 (0.82, 2.91)**	−0.45 (−1.89, 0.99)	0.26 (−1.09, 1.61)	**0.60 (0.06, 1.15)**	**1.16 (0.10, 2.22)**	0.78 (−0.31, 1.87)	**−4.91 (−6.92**, **−2.91)**
CCL3			1.14 (−0.21, 2.49)	0.35 (−1.05, 1.74)	0.35 (−1.23, 1.93)	**1.26 (0.09, 2.43)**	−1.05 (−2.63, 0.52)	−0.34 (−1.73, 1.06)	0.00 (−0.72, 0.73)	0.56 (−0.59, 1.70)	0.18 (−1.08, 1.45)	**−5.51 (−7.58**, **−3.45)**
CCL4				−0.80 (−2.61, 1.01)	−0.79 (−2.71, 1.12)	0.12 (−1.47, 1.71)	**−2.19 (−4.10**, **−0.29)**	−1.48 (−3.29, 0.33)	−1.14 (−2.44, 0.16)	−0.59 (−2.06, 0.89)	−0.96 (−2.62, 0.70)	**−6.66 (−8.99**, **−4.33)**
CCL13					0.00 (−1.95, 1.95)	0.92 (−0.72, 2.55)	−1.40 (−3.34, 0.55)	−0.68 (−2.33, 0.96)	−0.34 (−1.69, 1.01)	0.21 (−1.43, 1.86)	−0.16 (−1.86, 1.54)	**−5.86 (−8.22**, **−3.50)**
CCL19						0.91 (−0.81, 2.63)	−1.40 (−3.05, 0.25)	−0.69 (−2.63, 1.26)	−0.34 (−1.84, 1.15)	0.21 (−1.54, 1.96)	−0.17 (−1.95, 1.62)	**−5.86 (−8.29**, **−3.44)**
CCL20							**−2.31 (−4.03**, **−0.60)**	−1.60 (−3.23, 0.03)	**−1.26 (−2.29**, **−0.22)**	−0.70 (−2.08, 0.67)	−1.08 (−2.52, 0.36)	**−6.78 (−8.93**, **−4.62)**
CCL21								0.71 (−1.23, 2.65)	1.06 (−0.43, 2.54)	1.61 (−0.13, 3.35)	1.23 (−0.54, 3.01)	**−4.46 (−6.88**, **−2.04)**
CCL22									0.34 (−1.00, 1.69)	0.90 (−0.75, 2.54)	0.52 (−1.18, 2.22)	**−5.18 (−7.54**, **−2.82)**
CXCL8										0.55 (−0.50, 1.61)	0.18 (−0.98, 1.33)	**−5.52 (−7.51**, **−3.52)**
CXCL10											−0.38 (−1.84, 1.09)	**−6.07 (−8.26**, **−3.88)**
CXCL12												**−5.70 (−7.93**, **−3.47)**

*Numbers above the diagonal show standardized mean differences in effect size and 95% CI. Means more than 0 indicate that the chemokines on that row reduce in NASH than the chemokines on that column. NASH, non-alcoholic steatohepatitis. The bold values means the SMD values with statistically significant (p < 0.05)*.

**Figure 3:**
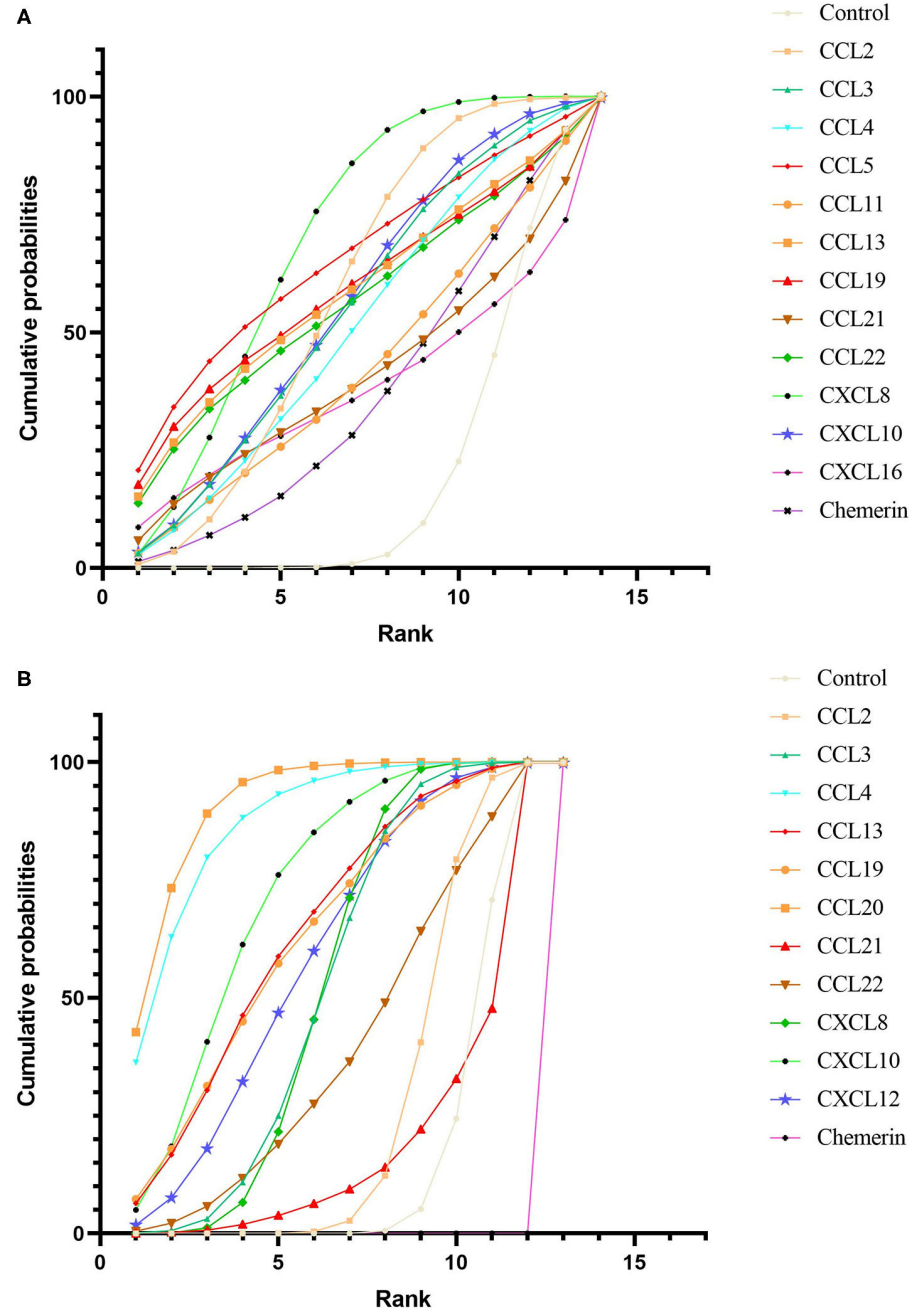
Cumulative rank probability plot of chemokines comparisons for NAFL **(A)** and NASH **(B)**. This plot shows the relative cumulative probabilities for each of the chemokines in the network. SUCRA values are presented in the legend. SUCRA, surface under cumulative ranking curve.

After obtaining ranking graphs of probability distributions of chemokines, the SUCRA probabilities of all chemokines were compared (Appendix 5 in Supplementary Material and Figure 3). Accordingly, SUCRA probabilities showed that CXCL8 was ranked the highest in NAFL, followed by CCL5, CCL19, CCL2, CCL13, CCL22, CXCL10, CCL3, CCL4, CCL11, CCL21, CXCL16, and chemerin. Additionally, SUCRA for CCL20 was 91.5%, which was ranked the highest in NASH, followed by CCL4, CXCL10, CCL13, CCL19, CXCL12, CCL3, CXCL8, CCL22, CCL2, CCL21, and chemerin. Comparison-adjusted funnel plots for NAFL and NASH showed no evidence of asymmetry (Appendix 6 in Supplementary Material). Study quality assessment using NOS indicated that 25 studies were of moderate quality, whereas 21 were of high quality. Each GRADE framework of the main network outcomes is shown in Appendix 7 (Supplementary Material). Heterogeneity analysis showed that most of the loop-specific heterogeneity confidence intervals included 0, suggesting that the heterogeneity was not significant, but some of them reported large IF values, suggesting that the direct comparison results were slightly different from the indirect comparison results, hence these findings should be interpreted with caution (Appendix 8 in Supplementary Material). Finally, sensitivity analyses for the rank order (SUCRA ranks) in different chemokines indicated that any single study influenced little change in the rank order (Appendix 9 in Supplementary Material).

## Discussion

This study is based on 46 studies, which included 4,753 NAFL/NASH patients and 4,059 controls, to study the relationship between chemokines concentrations and NAFL/NASH. Specifically, the outcomes of the 46 studies were synthesized using network meta-analysis to quantify this relationship. To the best of our knowledge, this is the largest contemporary systematic review of chemokine concentrations in NAFLD (NAFL/NASH). Basically, the approach in this network meta-analysis was to analyze the concentration differences of chemokines between the NAFL/NASH patients and the controls and rank the chemokines in question according to their effect sizes. The results indicated that there was a significant increase in the concentrations of CCL2 and CXCL8 in the NAFL group when compared with the control group; and the concentrations of CCL3, CCL4, CCL20, CXCL8, and CXCL10 were increased in the NASH group when compared with the control group. In addition, SUCRA probabilities showed that CCL20 ranked highest in NASH in terms of the effect size on NAFL/NASH.

According to experimental studies, hepatic stellate cells (HSCs), Kupffer cells, and portal fibroblasts play a crucial role in the pathophysiological progression of NASH (8). They secrete large amounts of chemokines, oxygen radicals, and collagens via autocrine/paracrine. Upon binding to related receptors, these chemokines promote the development of liver fibrosis by producing large amounts of extracellular matrix proteins (6). Interestingly, activation phosphorylation of the extracellular signal-regulated kinase (eRK) can bring about migration and proliferation of human HSCs, hence enhancing these effects (19). Moreover, during the transition from NAFL to NASH, chemokines recruit immune cells and infiltrate into the liver and adipose tissue, such as GR1+ monocytes, which play a role in pro-inflammation, systemic insulin resistance, and pro-liver fibrosis by producing inducible nitric oxide synthase (20). Therefore, it has been suggested that CXC chemokines participate in acute inflammation, while most CC chemokines are involved in the mediation of chronic inflammation (8, 9). Furthermore, previous studies have demonstrated that CXCL9, CXCL10, and CXCL11 can attract T cells to the liver (21). Resident natural killer T cells expressing CXCR6 in these T cells can modulate the inflammatory response in the liver and migrate with the local secretion of CXCL16. On the other hand, T helper type 2 lymphocytes are attracted by CCL17 and CCL22, which helps inhibit the expression of pro-inflammatory cells (22). Regulatory T cells are attracted by CXCL9 secretion, which inhibits the pro-inflammatory response (21). Besides, CCL2, CCL3, CCL5, CXCL4, CXCL10, and CXCL16 promote fibrosis by activating or attracting HSCs, while CX3CL1 prevents fibrosis by affecting monocyte apoptosis (23).

Consistent with the results of this study, previous studies found that CCL2 signaling was associated with metabolic disorders during the development of non-alcoholic steatohepatitis and contributed to the lipid accumulation in hepatocytes. This could be explained by the fact that CCL2 and its receptors are upregulated in the liver, and this can promote macrophage accumulation in the liver and adipose tissue, which may lead to liver steatosis, hepatitis, and fibrosis. Moreover, several groups have reported that Kupffer cells and activated liver myofibroblasts secrete CXCL8 and CCL2 (24). Thus, it is well-known that CXCL8 is the key chemokine that attracts neutrophils, mainly releasing reactive oxygen species and protease through chemokine receptors, CXCR1 and CXCR2, thereby evoking hepatocyte necrosis (25). Meanwhile, damaged liver cells, activated HSCs, and Kupffer cells secrete high levels of CCL2, which further promotes the above pathophysiological processes via the activation of peroxisome proliferator-activated receptor α gene expression (26).

Additionally, experimental studies have shown that CCR2 and CCL2-deficient mice exhibited attenuated inflammation in adipose tissue, decreased adipose tissue macrophages, and protection against high fat diet-induced insulin resistance. Interestingly, mice treated with a pharmacological antagonist of CCR2 showed increased insulin sensitivity and decreased adipose tissue macrophages levels without weight loss (27). Besides, clinical studies have shown that the plasma CCL2 concentrations were higher in obese patients than in lean individuals, for both adults and children (28). In spite of that, another clinical report showed that obese patients showed a corresponding decrease in CCL2 concentrations after lifestyle improvement program.

Recently, experimental studies have shown that CXCL8 is a CXC chemokine with pro-inflammatory and pro-angiogenesis properties (25). Macrophages are a major component of NAFL and NASH, and studies have shown that these can produce higher levels of CXCL8 in coculture of liver-activated macrophages, hence inducing CXCL8/mir-17 clusters. Therefore, CXCL8 may play a role in the recruitment of neutrophils in the process of NASH via activation of the AKT/mTOR/STAT3 pathway. At the same time, the intrahepatic expression of CXCL8 in the blood and liver was upregulated in patients with NAFL. In connection with the preceding evidence, this study found that CXCL8 may play an important role in both NAFL and NASH stages, and its probability of SUCRA indicated that CXCL8 ranked the highest in NAFL (25). Therefore, CXCL8 could be considered as an important marker and potential therapeutic target for NAFL and NASH.

Furthermore, studies revealed that high levels of CCL20 are released by injured hepatocytes and adipocytes to stimulate the migration of CCR6-expressing Th17 cells and gd T cells into injured liver via PI3K/AKT and Wnt/β-catenin pathways (29, 30). The Th17 cells in this process can induce the production of pro-inflammatory cytokines, tumor necrosis factor-alpha (TNF-a), IL-6, and IL-1, which may further aggravate hepatic inflammation (30). Noteworthily, experimental studies showed that *in vivo* knockdown of CCL20 reduced LPS-associated hepatic injury, suggesting that CCL20 may play a mediator role in hepatic inflammation, damage, and fibrosis in NAFL and NASH (31). This is consistent with results of this study in that SUCRA probabilities showed that CCL20 was ranked highest in NASH. Moreover, the nature and intensity of chemokines response can reflect the location and severity of hepatic damage, suggesting that, unlike other chemokines, CCL20 may play an important role in NASH, implying that it could be an attractive candidate for NASH therapeutic targeting.

Also, some previous studies indicated that, while inhibition of CCR1 and CCR5 inhibited the progression of liver fibrosis in mice, concentrations of CCl4 and CCl3 increased in mouse liver fibrogenesis models and cirrhotic patients. As regards CXCL10, preliminary studies indicated that it is secreted by liver sinusoidal endothelium when hepatocytes correlated with the severity of lobular inflammation (32). Thus, CXCL10 induced oxidative stress, fibrosis, and inflammation of NASH by activating the nuclear factor kappa-light-chain-enhancer via activated B-cells (NF-κB) pathway (32). In addition, some experimental data indicated that CXCL10 promoted steatosis by activating macrophages, hence causing associated fibrosis and hepatic injury in NASH.

Further, this study indicated that specific receptors, CCR1, CCR2, CCR5, CCR6, CXCR1, and CXCR2, bind to the above chemokines and may influence the progression of NAFL/NASH. Recent studies have shown that CCL3 and CCL4 have the same cognate chemokine receptors (CCR1 and CCR5), and the interactions between CCR1 and CCR5 promote fibrosis in mice by activation of HSCs and recruiting (bone marrow–derived) macrophages (33, 34). Mechanistically, the murine models lacking CCR1 and CCR5 show a reduced degree of liver fibrosis (33). Liver scarring in chimeric murine models revealed that CCR1 mediates its pro-fibrogenic effects in hematopoietic cells, whereas CCR5 mediates its pro-fibrogenic effects in hepatocytes (33).

Interestingly, a similar hypothesis has been proposed to suggest that the CCR2-CCL2 axis might be relevant to the progression of NAFL/NASH. In this regard, there has been evidence showing that obese mice have hepatic accumulation of bone marrow–derived CCR2-expressing Gr1+ monocytes, and these Gr1+ monocytes preferentially differentiate into macrophages, which produce inducible nitric oxide synthase and exert pro-fibrogenic and pro-inflammatory effects (20, 35). Moreover, in an experimental model, the pharmacological targeting of CCR2 antagonist prevented steatosis and reduced macrophage infiltration (36, 37). Therefore, pharmacological targeting of CCR2 may be more effective than CCL2 in directly affecting the progression of NAFL/NASH. However, pharmacological targeting of the CCR2 antagonist needs further investigation in murine models and preclinical trials of NAFL/NASH to ascertain its efficacy in blocking pro-inflammatory receptors.

Also, studies have shown that CCR6 has both pro-fibrogenic and pro-inflammatory effects (30, 38). Specifically, CCR6 has been associated with the positioning of IL-17-expressing T-helper cells (pro-fibrogenic) (30). On the other hand, CCL20 can attract lymphocytes and dendritic cells expressing CCR6 into the liver (pro-inflammatory) (38). The importance of CXCR1 and CXCR2 to the progression of NAFL/NASH has been further highlighted by experimental models (39). That is, analysis of experimental fibrosis and inflammation models revealed that CXCR1 and CXCR2 attract neutrophils to promote liver inflammatory response and liver injury (39, 40). Further, the infiltration of inflammatory neutrophils is characterized by high expression of CXCL8, CXCR1, and CXCR2, which recruit neutrophils into the liver to release reactive oxygen species as well as proteases leading to hepatocyte injury (39, 41).

Recently, studies have shown that, in addition to the foregoing conventional chemokine receptors (G-protein-coupled receptors), a distinct subfamily of chemokine receptors [atypical chemokine receptors (ACKRs)], involved in the fine-tuning chemokine-based responses in immunological microcircumstance, has been recognized (42). The ACKRs include, at present, four accepted members: ACKR1 (also known as Duffy antigen receptor for chemokine DARC), ACKR2 (also known as D6), ACKR3 (also known as CXCR7), and ACKR4 (also known as CCX-CKR) (43). Besides, ACKR5 has not yet been confirmed as a member of the ACKRs (43). These ACKR members are capable of binding to several chemokines and inhibiting their function by internalization, scavenge, or transportation of chemokines (Appendix 4d in Supplementary Material) (44). Therefore, although the ACKRs have not been recognized in the pathophysiology of NAFL/NASH, it can be conjectured that they control the immune and inflammatory responses. Furthermore, this study has shown that the concentrations of CCL2 and CXCL8 in the NAFL group were significantly higher than that in the control group, and the concentrations of CCL3, CCL4, CCL20, CXCL8, and CXCL10 in the NASH group were significantly higher than that in the control group. Thus, according to the chemokine-binding profiles of ACKRs, ACKR1 and ACKR2 may play a key role in the pathophysiology of NAFL/NASH. For example, previous studies have shown that ACKR1 binds numerous inflammatory CC and CXC chemokines (45, 46). In addition, clinical studies have shown that individuals with ACKR1 expression deficiency have higher concentrations of circulating inflammatory chemokines (46, 47). Functionally, ACKR1 has been suggested to act as either a chemokine transporter or a chemokine “sink” of inflammatory chemokines, thereby limiting excessive leukocyte extravasation (48). Moreover, comprehensive binding studies have shown that ACKR2 is of great value as an effective scavenger and degrader of inflammatory CC chemokines (48, 49). Also, ACKR2 appears to bind and internalize ligands to facilitate lysosomal degradation (50, 51). Thus, a better understanding of how ACKRs control the immune and inflammatory responses, at the molecular and cellular levels, in patients with NAFL/NASH may pave way toward novel and improved therapeutic strategies for NAFL/NASH.

Some strengths and potential limitations need to be considered when using the results of this study. First, although the comparisons involving some chemokines (only cited in a limited number of studies) in NAFL/NASH yielded significant results, more studies are needed in different populations to confirm this outcome. Besides, only overall chemokine levels were analyzed and this did not quantify potentially demographic changes and important clinical indicators at the individual patient level (due to lack of relevant information in the eligible studies used), such as smoking cigarettes, drinking alcohol, course of disease, stage of the disease, whether patients were receiving medication or not, physical activity, symptom severity, and blood pressure. These, therefore, should be taken into account in similar future investigations. Second, despite the lack of inconsistency and heterogeneity between direct and indirect comparisons, the models used in this study demonstrated the validity of the results. However, there was high loop-specific heterogeneity between some comparisons involving CCL4-CXCL10. Third, the original data used to accomplish the aims of this study came from cross-sectional studies, which cannot be used to make causal inferences. Therefore, large-scale population-based studies are needed in the future to examine the causal effects of chemokines in NAFL/NASH. Despite the foregoing limitations, chemokines are a diverse and systematic network that coordinates the protective and reparative processes in an injured liver by participating in the directing, mobilizing, and positioning of immune and inflammatory cells in NAFL.

## Conclusions

Based on the pathogeneses of NAFL and NASH, the findings of this study suggest that CCL2, CCL4, CCL20, CXCL8, and CXCL10 may play a role in the relationship between chemokines and NAFLD. Although there is coordination between chemokines and other inflammatory factor systems' signaling pathways, the findings of this study provide a rationale for evaluating chemokines in the pathophysiological process of NAFLD and might open up new perspectives in early diagnosis, identification of novel biomarkers, and providing novel targets for pharmacological interventions.

## Data Availability Statement

All datasets presented in this study are included in the article/Supplementary Material.

## Author Contributions

XP and JL collected the data, performed the statistical analyses, and wrote the manuscript with support from other authors. AL performed statistical analyses. JC and AC critically appraised study design and results. SW designed, supervised, and implemented the research. All authors discussed the results and contributed to the final manuscript.

## Conflict of Interest

The authors declare that the research was conducted in the absence of any commercial or financial relationships that could be construed as a potential conflict of interest.
