# Supplementary material for: Chemokines in Non-alcoholic Fatty Liver Disease: A Systematic Review and Network Meta-Analysis
Source: Front Immunol. 2020 Sep 18;11:1802. doi: 10.3389/fimmu.2020.01802 (PMC7530185; doi:10.3389/fimmu.2020.01802)
Supplement: Supplementary file 1 [file Table_1.DOC]

**Supplementary appendix**

**Contents**

**Appendix 1a:** Electronic search strategies

**Appendix 1b:** Study selection flow chart

**Appendix 2a:** Description of included studies

**Appendix 2b:** Characteristics of included studies

**Appendix 2c:** Original data of included studies

**Appendix 3a:** Local inconsistency for the network of Chemokines in NAFL group

**Appendix 3b:** Local inconsistency for the network of Chemokines in NASH group

**Appendix 4a:** The classification of chemokines and their receptors.

**Appendix 4b:** The distribution—cell type of chemokines receptors.

**Appendix 4c:** The suggested chemokines and chemokines receptors involvement in NAFLD.

**Appendix 4d:** The chemokine-binding profiles of the atypical chemokine receptors.

**Appendix 5a:** Rank probability of Chemokines

**Appendix 5b:** Rank and SUCRA of different chemokines

**Appendix 6:** Comparison-adjusted funnel plot

**Appendix 7a:** Risk of bias assessments

**Appendix 7b:** GRADE for the primary outcomes of NAFL group

**Appendix 7b:** GRADE for the primary outcomes of NASH group

**Appendix 8a:** Loop-Specific heterogeneity for the network of Chemokines in NAFL group

**Appendix 8b:** Loop-Specific heterogeneity for the network of Chemokines in NASH group

**Appendix 9a:** Sensitivity analyses for the rank order (SUCRA ranks) in differing chemokine in NAFL.

**Appendix 9b:** Sensitivity analyses for the rank order (SUCRA ranks) in differing chemokine in NASH.

**Appendices References**

**Appendix 1a: Electronic search strategies**

| **Search** | **Query** |
| --- | --- |
| 1 | chemokine* |
| 2 | ccl1 or ccl2 or ccl3 or ccl4 or ccl5 or ccl6 or ccl7 or ccl8 or ccl9 or ccl10 or ccl11 or ccl12 or ccl13 or ccl14 or ccl15 or ccl16 or ccl17 or ccl18 or ccl19 or ccl20 or ccl21 or ccl22 or ccl23 or ccl24 or ccl25 or ccl26 or ccl27 or ccl28 |
| 3 | cxcl1 or cxcl2 or cxcl3 or cxcl4 or cxcl5 or cxcl6 or cxcl7 or cxcl8 or cxcl9 or cxcl10 or cxcl11 or cxcl12 or cxcl13 or cxcl14 or cxcl15 or cxcl16 or cxcl17 |
| 4 | xcl1 or xcl2 |
| 5 | cx3cl1 |
| 6 | ccl or cxcl or xcl or cx3cl |
| 7 | scya1 or scya2 or scya3 or scya4 or scya5 or scya6 or scya7 or scya8 or scya9 or scya10 or scya11 or scya12 or scya13 or scya14 or scya15 or scya16 or scya17 or scya18 or scya19 or scya20 or scya21 or scya22 or scya23 or scya24 or scya25 or scya26 or scya27 or scya28 |
| 8 | scyb1 or scyb2 or scyb3 or scyb4 or scyb5 or scyb6 or scyb7 or scyb8 or scyb9 or scyb10 or scyb11 or scyb12 or scyb13 or scyb14 or scyb15 or scyb16 or scyb17 |
| 9 | scyc1 or scyc2 |
| 10 | sycd1 |
| 11 | scya or scyb or scyc or scyd |
| 12 | chemokine receptor* |
| 13 | ccr1 or ccr2 or ccr2b or ccr3 or ccr4 or ccr5 or ccr6 or ccr7 or ccr8 or ccr9 or ccr10 |
| 14 | cxcr1 or cxcr2 or cxcr3 or cxcr3b or cxcr4 or cxcr5 or cxcr6 or cxcr7 |
| 15 | xcr1 |
| 16 | cx3cr1 |
| 17 | ccr or cxcr or xcr or cx3cr |
| 18 | chemotactic cytokine* or chemokine* |
| 19 | i-309 or i309 or tca-3 or tca3 or sise |
| 20 | IL-8 or GCP-2 or CXCR1 or NAP-2 or ENA-78 or GROα or GROβ or GROγ or PF4 or IP-10 or MIG or I-TAC or SDF-1 or BCA-1 or SR-PSOX or BRAK or MCP-1 or MCP-4 or CCR2 or MCP-3 or MCP-2 or MIP-1β or MIP-1α or CCR5 or RANTES or MPIF-1 or HCC-1 or HCC-2 or HCC-4 or Eotaxin or Eotaxin-2 or Eotaxin-3 or TARC or CCR4 or MDC or MIP-3α or ELC or CCR7 or SLC or I-309 or TECK or CTACK or MEC or PARC or Lymphotactin or XCR1 or SCM-1β or Fractalkine or Chemerin |
| 21 | S1 OR S2 OR S3 OR S4 OR S5 OR S6 OR S7 OR S8 OR S9 OR S10 OR S11 OR S12 OR S13 OR S14 OR S15 OR S16 OR S17 OR S18 OR S19 OR S20 |
| 22 | Non alcoholic Fatty Liver Disease or NAFLD or Nonalcoholic Fatty Liver Disease |
| 23 | Non alcoholic Fatty Liver Disease* |
| 24 | Fatty Liver, Nonalcoholic or Fatty Livers, Nonalcoholic or Liver, Nonalcoholic Fatty or Livers, Nonalcoholic Fatty or Nonalcoholic Fatty Liver or Nonalcoholic Fatty Livers or Nonalcoholic Steatohepatitis or Nonalcoholic Steatohepatitides or Steatohepatitides, Nonalcoholic or Steatohepatitis, Nonalcoholic or Non-alcoholic Fatty Liver Disease or non-alcoholic steatohepatitis or NASH or Fatty Liver or nonalcoholic steatohepatitis or non-alcoholic fatty liver disease |
| 25 | S22 OR S23 OR S24 |
| 26 | S21 AND S25 |

**956 in** **Embase**

('non alcoholic fatty liver disease':ti OR nafld:ti OR 'nonalcoholic fatty liver disease':ti OR 'fatty liver, nonalcoholic':ti OR 'fatty livers, nonalcoholic':ti OR 'liver, nonalcoholic fatty':ti OR 'livers, nonalcoholic fatty':ti OR 'nonalcoholic fatty liver':ti OR 'nonalcoholic fatty livers':ti OR 'nonalcoholic steatohepatitides':ti OR 'steatohepatitides, nonalcoholic':ti OR 'steatohepatitis, nonalcoholic':ti OR 'non-alcoholic steatohepatitis':ti OR nash:ti OR 'fatty liver':ti OR 'nonalcoholic steatohepatitis':ti OR 'non-alcoholic fatty liver disease':ti) AND (ccl1:ti,ab,kw OR ccl2:ti,ab,kw OR ccl3:ti,ab,kw OR ccl4:ti,ab,kw OR ccl5:ti,ab,kw OR ccl6:ti,ab,kw OR ccl7:ti,ab,kw OR ccl8:ti,ab,kw OR ccl9:ti,ab,kw OR ccl10:ti,ab,kw OR ccl11:ti,ab,kw OR ccl12:ti,ab,kw OR ccl13:ti,ab,kw OR ccl14:ti,ab,kw OR ccl15:ti,ab,kw OR ccl16:ti,ab,kw OR ccl17:ti,ab,kw OR ccl18:ti,ab,kw OR ccl19:ti,ab,kw OR ccl20:ti,ab,kw OR ccl21:ti,ab,kw OR ccl22:ti,ab,kw OR ccl23:ti,ab,kw OR ccl24:ti,ab,kw OR ccl25:ti,ab,kw OR ccl26:ti,ab,kw OR ccl27:ti,ab,kw OR ccl28:ti,ab,kw OR cxcl1:ti,ab,kw OR cxcl2:ti,ab,kw OR cxcl3:ti,ab,kw OR cxcl4:ti,ab,kw OR cxcl5:ti,ab,kw OR cxcl6:ti,ab,kw OR cxcl7:ti,ab,kw OR cxcl8:ti,ab,kw OR cxcl9:ti,ab,kw OR cxcl10:ti,ab,kw OR cxcl11:ti,ab,kw OR cxcl12:ti,ab,kw OR cxcl13:ti,ab,kw OR cxcl14:ti,ab,kw OR cxcl15:ti,ab,kw OR cxcl16:ti,ab,kw OR cxcl17:ti,ab,kw OR xcl1:ti,ab,kw OR xcl2:ti,ab,kw OR cx3cl1:ti,ab,kw OR ccl:ti,ab,kw OR cxcl:ti,ab,kw OR xcl:ti,ab,kw OR cx3cl:ti,ab,kw OR scya1:ti,ab,kw OR scya2:ti,ab,kw OR scya3:ti,ab,kw OR scya4:ti,ab,kw OR scya5:ti,ab,kw OR scya6:ti,ab,kw OR scya7:ti,ab,kw OR scya8:ti,ab,kw OR scya9:ti,ab,kw OR scya10:ti,ab,kw OR scya11:ti,ab,kw OR scya12:ti,ab,kw OR scya13:ti,ab,kw OR scya14:ti,ab,kw OR scya15:ti,ab,kw OR scya16:ti,ab,kw OR scya17:ti,ab,kw OR scya18:ti,ab,kw OR scya19:ti,ab,kw OR scya20:ti,ab,kw OR scya21:ti,ab,kw OR scya22:ti,ab,kw OR scya23:ti,ab,kw OR scya24:ti,ab,kw OR scya25:ti,ab,kw OR scya26:ti,ab,kw OR scya27:ti,ab,kw OR scya28:ti,ab,kw OR scyb1:ti,ab,kw OR scyb2:ti,ab,kw OR scyb3:ti,ab,kw OR scyb4:ti,ab,kw OR scyb5:ti,ab,kw OR scyb6:ti,ab,kw OR scyb7:ti,ab,kw OR scyb8:ti,ab,kw OR scyb9:ti,ab,kw OR scyb10:ti,ab,kw OR scyb11:ti,ab,kw OR scyb12:ti,ab,kw OR scyb13:ti,ab,kw OR scyb14:ti,ab,kw OR scyb15:ti,ab,kw OR scyb16:ti,ab,kw OR scyb17:ti,ab,kw OR scyc1:ti,ab,kw OR scyc2:ti,ab,kw OR sycd1:ti,ab,kw OR scya:ti,ab,kw OR scyb:ti,ab,kw OR scyc:ti,ab,kw OR scyd:ti,ab,kw OR 'chemokine receptor*':ti,ab,kw OR ccr1:ti,ab,kw OR ccr2b:ti,ab,kw OR ccr3:ti,ab,kw OR ccr6:ti,ab,kw OR ccr8:ti,ab,kw OR ccr9:ti,ab,kw OR ccr10:ti,ab,kw OR cxcr2:ti,ab,kw OR cxcr3:ti,ab,kw OR cxcr3b:ti,ab,kw OR cxcr4:ti,ab,kw OR cxcr5:ti,ab,kw OR cxcr6:ti,ab,kw OR cxcr7:ti,ab,kw OR cx3cr1:ti,ab,kw OR ccr:ti,ab,kw OR cxcr:ti,ab,kw OR xcr:ti,ab,kw OR cx3cr:ti,ab,kw OR 'chemotactic cytokine*':ti,ab,kw OR chemokine*:ti,ab,kw OR i309:ti,ab,kw OR 'tca 3':ti,ab,kw OR tca3:ti,ab,kw OR sise:ti,ab,kw OR 'il 8':ti,ab,kw OR 'gcp 2':ti,ab,kw OR cxcr1:ti,ab,kw OR 'nap 2':ti,ab,kw OR 'ena 78':ti,ab,kw OR groα:ti,ab,kw OR groβ:ti,ab,kw OR groγ:ti,ab,kw OR pf4:ti,ab,kw OR 'ip 10':ti,ab,kw OR mig:ti,ab,kw OR 'i tac':ti,ab,kw OR 'sdf 1':ti,ab,kw OR 'bca 1':ti,ab,kw OR 'sr psox':ti,ab,kw OR brak:ti,ab,kw OR 'mcp 1':ti,ab,kw OR 'mcp 4':ti,ab,kw OR ccr2:ti,ab,kw OR 'mcp 3':ti,ab,kw OR 'mcp 2':ti,ab,kw OR 'mip 1β':ti,ab,kw OR 'mip 1α':ti,ab,kw OR ccr5:ti,ab,kw OR rantes:ti,ab,kw OR 'mpif 1':ti,ab,kw OR 'hcc 1':ti,ab,kw OR 'hcc 2':ti,ab,kw OR 'hcc 4':ti,ab,kw OR eotaxin:ti,ab,kw OR 'eotaxin 2':ti,ab,kw OR 'eotaxin 3':ti,ab,kw OR tarc:ti,ab,kw OR ccr4:ti,ab,kw OR mdc:ti,ab,kw OR 'mip 3α':ti,ab,kw OR elc:ti,ab,kw OR ccr7:ti,ab,kw OR slc:ti,ab,kw OR 'i 309':ti,ab,kw OR teck:ti,ab,kw OR ctack:ti,ab,kw OR mec:ti,ab,kw OR parc:ti,ab,kw OR lymphotactin:ti,ab,kw OR xcr1:ti,ab,kw OR 'scm 1β':ti,ab,kw OR fractalkine:ti,ab,kw OR chemerin:ti,ab,kw)

**736 in PubMed：**

(((Non alcoholic Fatty Liver Disease[Title/Abstract] OR NAFLD[Title/Abstract] OR Nonalcoholic Fatty Liver Disease[Title/Abstract]) OR (non alcoholic fatty liver disease[All Fields] OR non alcoholic fatty liver diseases[All Fields])) OR (Fatty Liver, Nonalcoholic[Title/Abstract] OR (("fatty liver"[MeSH Terms] OR ("fatty"[All Fields] AND "liver"[All Fields]) OR "fatty liver"[All Fields] OR ("fatty"[All Fields] AND "livers"[All Fields]) OR "fatty livers"[All Fields]) AND Nonalcoholic[Title/Abstract]) OR Liver, Nonalcoholic Fatty[Title/Abstract] OR (("liver"[MeSH Terms] OR "liver"[All Fields] OR "livers"[All Fields]) AND Nonalcoholic Fatty[Title/Abstract]) OR Nonalcoholic Fatty Liver[Title/Abstract] OR Nonalcoholic Fatty Livers[Title/Abstract] OR Nonalcoholic Steatohepatitis[Title/Abstract] OR (("fatty liver"[MeSH Terms] OR ("fatty"[All Fields] AND "liver"[All Fields]) OR "fatty liver"[All Fields]) AND Nonalcoholic[Title/Abstract]) OR (("fatty liver"[MeSH Terms] OR ("fatty"[All Fields] AND "liver"[All Fields]) OR "fatty liver"[All Fields] OR "steatohepatitis"[All Fields]) AND Nonalcoholic[Title/Abstract]) OR Non-alcoholic Fatty Liver Disease[Title/Abstract] OR non-alcoholic steatohepatitis[Title/Abstract] OR NASH[Title/Abstract] OR Fatty Liver[Title/Abstract] OR nonalcoholic steatohepatitis[Title/Abstract] OR non-alcoholic fatty liver disease[Title/Abstract])) AND (((((((((((((((((((chemokine[Title/Abstract] OR chemokine'[Title/Abstract] OR chemokine's[Title/Abstract] OR chemokine1[Title/Abstract] OR chemokine11[Title/Abstract] OR chemokine7[Title/Abstract] OR chemokineactivated[Title/Abstract] OR chemokineand[Title/Abstract] OR chemokinehicytokinehipd[Title/Abstract] OR chemokineinduced[Title/Abstract] OR chemokinelike[Title/Abstract] OR chemokinemediated[Title/Abstract] OR chemokinemia[Title/Abstract] OR chemokiner[Title/Abstract] OR chemokinereceptor[Title/Abstract] OR chemokinereceptors[Title/Abstract] OR chemokinerelated[Title/Abstract] OR chemokinergic[Title/Abstract] OR chemokines[Title/Abstract] OR chemokines'[Title/Abstract] OR chemokineschemokines[Title/Abstract] OR chemokinese[Title/Abstract] OR chemokineses[Title/Abstract] OR chemokinesin[Title/Abstract] OR chemokinesins[Title/Abstract] OR chemokinesis[Title/Abstract] OR chemokinesplay[Title/Abstract] OR chemokinetic[Title/Abstract] OR chemokinetically[Title/Abstract] OR chemokinetics[Title/Abstract]) OR (ccl1[Title/Abstract] OR ccl2[Title/Abstract] OR ccl3[Title/Abstract] OR ccl4[Title/Abstract] OR ccl5[Title/Abstract] OR ccl6[Title/Abstract] OR ccl7[Title/Abstract] OR ccl8[Title/Abstract] OR ccl9[Title/Abstract] OR ccl10[Title/Abstract] OR ccl11[Title/Abstract] OR ccl12[Title/Abstract] OR ccl13[Title/Abstract] OR ccl14[Title/Abstract] OR ccl15[Title/Abstract] OR ccl16[Title/Abstract] OR ccl17[Title/Abstract] OR ccl18[Title/Abstract] OR ccl19[Title/Abstract] OR ccl20[Title/Abstract] OR ccl21[Title/Abstract] OR ccl22[Title/Abstract] OR ccl23[Title/Abstract] OR ccl24[Title/Abstract] OR ccl25[Title/Abstract] OR ccl26[Title/Abstract] OR ccl27[Title/Abstract] OR ccl28[Title/Abstract])) OR (cxcl1[Title/Abstract] OR cxcl2[Title/Abstract] OR cxcl3[Title/Abstract] OR cxcl4[Title/Abstract] OR cxcl5[Title/Abstract] OR cxcl6[Title/Abstract] OR cxcl7[Title/Abstract] OR cxcl8[Title/Abstract] OR cxcl9[Title/Abstract] OR cxcl10[Title/Abstract] OR cxcl11[Title/Abstract] OR cxcl12[Title/Abstract] OR cxcl13[Title/Abstract] OR cxcl14[Title/Abstract] OR cxcl15[Title/Abstract] OR cxcl16[Title/Abstract] OR cxcl17[Title/Abstract])) OR (xcl1[Title/Abstract] OR xcl2[Title/Abstract])) OR cx3cl1[Title/Abstract]) OR (ccl[Title/Abstract] OR cxcl[Title/Abstract] OR xcl[Title/Abstract] OR cx3cl[Title/Abstract])) OR (scya1[Title/Abstract] OR scya2[Title/Abstract] OR scya3[Title/Abstract] OR scya4[Title/Abstract] OR scya5[Title/Abstract] OR scya6[Title/Abstract] OR scya7[Title/Abstract] OR scya8[Title/Abstract] OR scya9[Title/Abstract] OR scya10[Title/Abstract] OR scya11[Title/Abstract] OR scya12[Title/Abstract] OR scya13[Title/Abstract] OR scya14[Title/Abstract] OR scya15[Title/Abstract] OR scya16[Title/Abstract] OR scya17[Title/Abstract] OR scya18[Title/Abstract] OR scya19[Title/Abstract] OR scya20[Title/Abstract] OR scya21[Title/Abstract] OR scya22[Title/Abstract] OR scya23[Title/Abstract] OR scya24[Title/Abstract] OR scya25[Title/Abstract] OR scya26[Title/Abstract])) OR (scyb1[Title/Abstract] OR scyb2[Title/Abstract] OR scyb5[Title/Abstract] OR scyb6[Title/Abstract] OR scyb9[Title/Abstract] OR scyb10[Title/Abstract] OR scyb11[Title/Abstract] OR scyb12[Title/Abstract] OR scyb13[Title/Abstract] OR scyb14[Title/Abstract])) OR scyc1[Title/Abstract]) OR (scya[Title/Abstract] OR scyb[Title/Abstract] OR scyc[Title/Abstract] OR scyd[Title/Abstract])) OR (chemokine receptor[Title/Abstract] OR chemokine receptors[Title/Abstract])) OR (ccr1[Title/Abstract] OR ccr2[Title/Abstract] OR ccr2b[Title/Abstract] OR ccr3[Title/Abstract] OR ccr4[Title/Abstract] OR ccr5[Title/Abstract] OR ccr6[Title/Abstract] OR ccr7[Title/Abstract] OR ccr8[Title/Abstract] OR ccr9[Title/Abstract] OR ccr10[Title/Abstract])) OR (cxcr1[Title/Abstract] OR cxcr2[Title/Abstract] OR cxcr3[Title/Abstract] OR cxcr3b[Title/Abstract] OR cxcr4[Title/Abstract] OR cxcr5[Title/Abstract] OR cxcr6[Title/Abstract] OR cxcr7[Title/Abstract])) OR xcr1[Title/Abstract]) OR cx3cr1[Title/Abstract]) OR (ccr[Title/Abstract] OR cxcr[Title/Abstract] OR xcr[Title/Abstract] OR cx3cr[Title/Abstract])) OR ((chemotactic cytokine[Title/Abstract] OR chemotactic cytokines[Title/Abstract]) OR (chemokine[Title/Abstract] OR chemokine'[Title/Abstract] OR chemokine's[Title/Abstract] OR chemokine1[Title/Abstract] OR chemokine11[Title/Abstract] OR chemokine7[Title/Abstract] OR chemokineactivated[Title/Abstract] OR chemokineand[Title/Abstract] OR chemokinehicytokinehipd[Title/Abstract] OR chemokineinduced[Title/Abstract] OR chemokinelike[Title/Abstract] OR chemokinemediated[Title/Abstract] OR chemokinemia[Title/Abstract] OR chemokiner[Title/Abstract] OR chemokinereceptor[Title/Abstract] OR chemokinereceptors[Title/Abstract] OR chemokinerelated[Title/Abstract] OR chemokinergic[Title/Abstract] OR chemokines[Title/Abstract] OR chemokines'[Title/Abstract] OR chemokineschemokines[Title/Abstract] OR chemokinese[Title/Abstract] OR chemokineses[Title/Abstract] OR chemokinesin[Title/Abstract] OR chemokinesins[Title/Abstract] OR chemokinesis[Title/Abstract] OR chemokinesplay[Title/Abstract] OR chemokinetic[Title/Abstract] OR chemokinetically[Title/Abstract] OR chemokinetics[Title/Abstract]))) OR (i-309[Title/Abstract] OR i309[Title/Abstract] OR tca-3[Title/Abstract] OR tca3[Title/Abstract] OR sise[Title/Abstract])) OR (IL-8[Title/Abstract] OR GCP-2[Title/Abstract] OR CXCR1[Title/Abstract] OR NAP-2[Title/Abstract] OR ENA-78[Title/Abstract] OR GROalpha[Title/Abstract] OR GRObeta[Title/Abstract] OR GROgamma[Title/Abstract] OR PF4[Title/Abstract] OR IP-10[Title/Abstract] OR MIG[Title/Abstract] OR I-TAC[Title/Abstract] OR SDF-1[Title/Abstract] OR BCA-1[Title/Abstract] OR SR-PSOX[Title/Abstract] OR BRAK[Title/Abstract] OR MCP-1[Title/Abstract] OR MCP-4[Title/Abstract] OR CCR2[Title/Abstract] OR MCP-3[Title/Abstract] OR MCP-2[Title/Abstract] OR MIP-1beta[Title/Abstract] OR MIP-1alpha[Title/Abstract] OR CCR5[Title/Abstract] OR RANTES[Title/Abstract] OR MPIF-1[Title/Abstract] OR HCC-1[Title/Abstract] OR HCC-2[Title/Abstract] OR HCC-4[Title/Abstract] OR Eotaxin[Title/Abstract] OR Eotaxin-2[Title/Abstract] OR Eotaxin-3[Title/Abstract] OR TARC[Title/Abstract] OR CCR4[Title/Abstract] OR MDC[Title/Abstract] OR MIP-3alpha[Title/Abstract] OR ELC[Title/Abstract] OR CCR7[Title/Abstract] OR SLC[Title/Abstract] OR I-309[Title/Abstract] OR TECK[Title/Abstract] OR CTACK[Title/Abstract] OR MEC[Title/Abstract] OR PARC[Title/Abstract] OR Lymphotactin[Title/Abstract] OR XCR1[Title/Abstract] OR SCM-1beta[Title/Abstract] OR Chemerin[Title/Abstract] OR Fractalkine[Title/Abstract]))

**915 in Web of Science**

TI=( Non alcoholic Fatty Liver Disease OR NAFLD OR Nonalcoholic Fatty Liver Disease OR Fatty Liver, Nonalcoholic OR Fatty Livers, Nonalcoholic OR Liver, Nonalcoholic Fatty OR Livers, Nonalcoholic Fatty OR Nonalcoholic Fatty Liver OR Nonalcoholic Fatty Livers OR Nonalcoholic Steatohepatitis OR Nonalcoholic Steatohepatitides OR Steatohepatitides, Nonalcoholic OR Steatohepatitis, Nonalcoholic OR Non-alcoholic Fatty Liver Disease OR non-alcoholic steatohepatitis OR NASH OR Fatty Liver OR nonalcoholic steatohepatitis OR non-alcoholic fatty liver disease) AND TS= (chemokine* or ccl1 or ccl2 or ccl3 or ccl4 or ccl5 or ccl6 or ccl7 or ccl8 or ccl9 or ccl10 or ccl11 or ccl12 or ccl13 or ccl14 or ccl15 or ccl16 or ccl17 or ccl18 or ccl19 or ccl20 or ccl21 or ccl22 or ccl23 or ccl24 or ccl25 or ccl26 or ccl27 or ccl28 or cxcl1 or cxcl2 or cxcl3 or cxcl4 or cxcl5 or cxcl6 or cxcl7 or cxcl8 or cxcl9 or cxcl10 or cxcl11 or cxcl12 or cxcl13 or cxcl14 or cxcl15 or cxcl16 or cxcl17 or xcl1 or xcl2 or cx3cl1 or ccl or cxcl or xcl or cx3cl or scya1 or scya2 or scya3 or scya4 or scya5 or scya6 or scya7 or scya8 or scya9 or scya10 or scya11 or scya12 or scya13 or scya14 or scya15 or scya16 or scya17 or scya18 or scya19 or scya20 or scya21 or scya22 or scya23 or scya24 or scya25 or scya26 or scya27 or scya28 or scyb1 or scyb2 or scyb3 or scyb4 or scyb5 or scyb6 or scyb7 or scyb8 or scyb9 or scyb10 or scyb11 or scyb12 or scyb13 or scyb14 or scyb15 or scyb16 or scyb17 or scyc1 or scyc2 or sycd1 or scya or scyb or scyc or scyd or chemokine receptor* or ccr1 or ccr2 or ccr2b or ccr3 or ccr4 or ccr5 or ccr6 or ccr7 or ccr8 or ccr9 or ccr10 or cxcr1 or cxcr2 or cxcr3 or cxcr3b or cxcr4 or cxcr5 or cxcr6 or cxcr7 or xcr1 or cx3cr1 or ccr or cxcr or xcr or cx3cr or chemotactic cytokine* or chemokine* or i-309 or i309 or tca-3 or tca3 or sise or IL-8 or GCP-2 or CXCR1 or NAP-2 or ENA-78 or GROα or GROβ or GROγ or PF4 or IP-10 or MIG or I-TAC or SDF-1 or BCA-1 or SR-PSOX or BRAK or MCP-1 or MCP-4 or CCR2 or MCP-3 or MCP-2 or MIP-1β or MIP-1α or CCR5 or RANTES or MPIF-1 or HCC-1 or HCC-2 or HCC-4 or Eotaxin or Eotaxin-2 or Eotaxin-3 or TARC or CCR4 or MDC or MIP-3α or ELC or CCR7 or SLC or I-309 or TECK or CTACK or MEC or PARC or Lymphotactin or XCR1 or SCM-1β or Fractalkine or Chemerin)

**91 in** **Cochrane library**

Non alcoholic Fatty Liver Disease OR NAFLD OR Nonalcoholic Fatty Liver Disease OR Fatty Liver, Nonalcoholic OR Fatty Livers, Nonalcoholic OR Liver, Nonalcoholic Fatty OR Livers, Nonalcoholic Fatty OR Nonalcoholic Fatty Liver OR Nonalcoholic Fatty Livers OR Nonalcoholic Steatohepatitis OR Nonalcoholic Steatohepatitides OR Steatohepatitides, Nonalcoholic OR Steatohepatitis, Nonalcoholic OR Non-alcoholic Fatty Liver Disease OR non-alcoholic steatohepatitis OR NASH OR Fatty Liver OR nonalcoholic steatohepatitis OR non-alcoholic fatty liver disease in All Text AND chemokine* or ccl1 or ccl2 or ccl3 or ccl4 or ccl5 or ccl6 or ccl7 or ccl8 or ccl9 or ccl10 or ccl11 or ccl12 or ccl13 or ccl14 or ccl15 or ccl16 or ccl17 or ccl18 or ccl19 or ccl20 or ccl21 or ccl22 or ccl23 or ccl24 or ccl25 or ccl26 or ccl27 or ccl28 or cxcl1 or cxcl2 or cxcl3 or cxcl4 or cxcl5 or cxcl6 or cxcl7 or cxcl8 or cxcl9 or cxcl10 or cxcl11 or cxcl12 or cxcl13 or cxcl14 or cxcl15 or cxcl16 or cxcl17 or xcl1 or xcl2 or cx3cl1 or ccl or cxcl or xcl or cx3cl or scya1 or scya2 or scya3 or scya4 or scya5 or scya6 or scya7 or scya8 or scya9 or scya10 or scya11 or scya12 or scya13 or scya14 or scya15 or scya16 or scya17 or scya18 or scya19 or scya20 or scya21 or scya22 or scya23 or scya24 or scya25 or scya26 or scya27 or scya28 or scyb1 or scyb2 or scyb3 or scyb4 or scyb5 or scyb6 or scyb7 or scyb8 or scyb9 or scyb10 or scyb11 or scyb12 or scyb13 or scyb14 or scyb15 or scyb16 or scyb17 or scyc1 or scyc2 or sycd1 or scya or scyb or scyc or scyd or chemokine receptor* or ccr1 or ccr2 or ccr2b or ccr3 or ccr4 or ccr5 or ccr6 or ccr7 or ccr8 or ccr9 or ccr10 or cxcr1 or cxcr2 or cxcr3 or cxcr3b or cxcr4 or cxcr5 or cxcr6 or cxcr7 or xcr1 or cx3cr1 or ccr or cxcr or xcr or cx3cr or chemotactic cytokine* or chemokine* or i-309 or i309 or tca-3 or tca3 or sise or IL-8 or GCP-2 or CXCR1 or NAP-2 or ENA-78 or GROα or GROβ or GROγ or PF4 or IP-10 or MIG or I-TAC or SDF-1 or BCA-1 or SR-PSOX or BRAK or MCP-1 or MCP-4 or CCR2 or MCP-3 or MCP-2 or MIP-1β or MIP-1α or CCR5 or RANTES or MPIF-1 or HCC-1 or HCC-2 or HCC-4 or Eotaxin or Eotaxin-2 or Eotaxin-3 or TARC or CCR4 or MDC or MIP-3α or ELC or CCR7 or SLC or I-309 or TECK or CTACK or MEC or PARC or Lymphotactin or XCR1 or SCM-1β or Fractalkine or Chemerin in All Text

**Appendix 1b: Study selection flow chart. A flow chart demonstrating the selection process of articles included in the analysis as well as in the qualitative summary.**


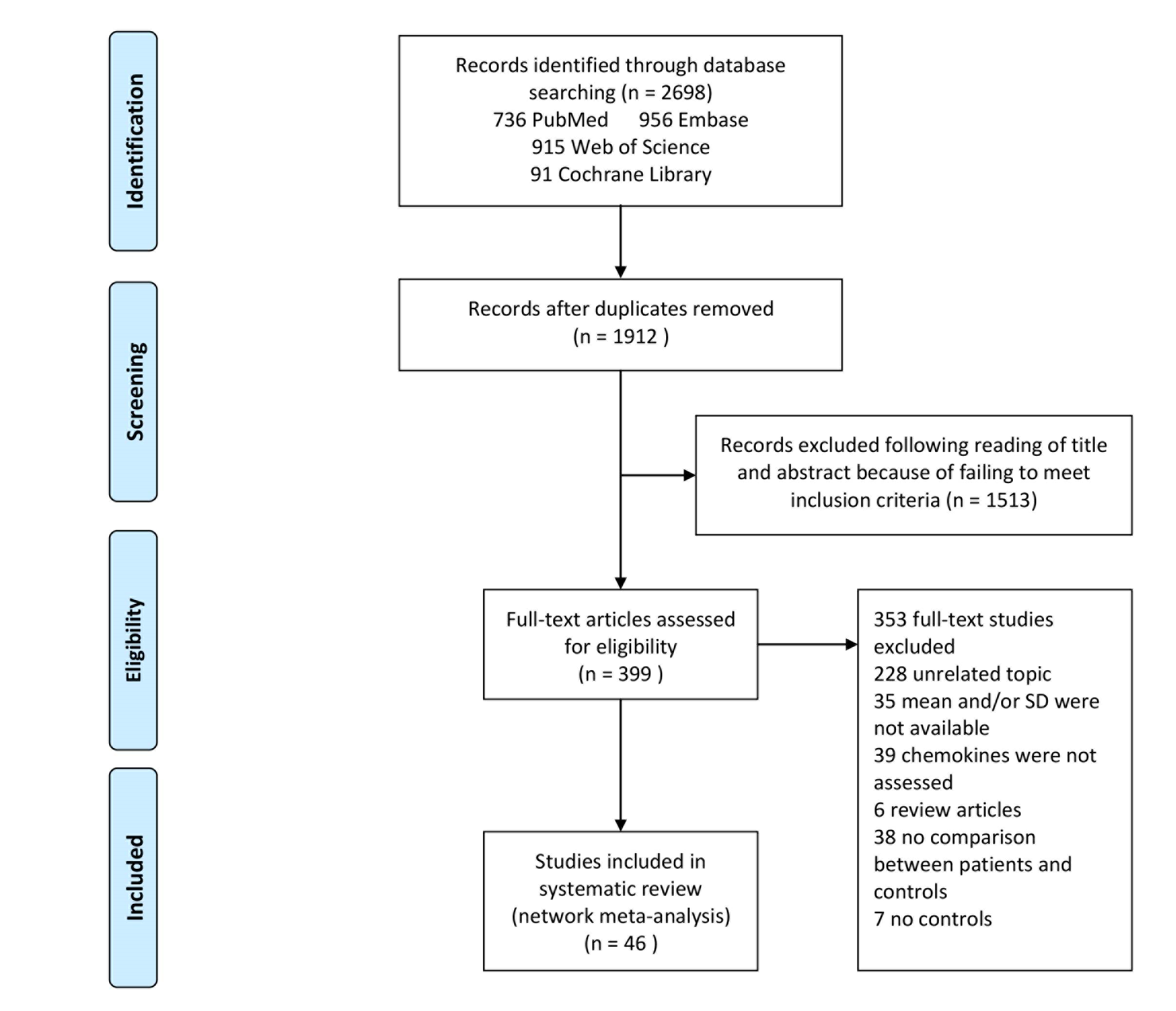


Appendix 2a: Description of included studies

| **Study** |  | **Chemokines** | **Material** | **Country** | **NOS** | **Diagnosis of NAFLD** | **Methods** | **Frozen** | **NAFLD type** | **Stage of the disease** | **Medication intervention** |
| --- | --- | --- | --- | --- | --- | --- | --- | --- | --- | --- | --- |
| Ajmera 2017 | [1] | CCL2, CXCL8 | Plasma | USA | 7 | Liver biopsy specimens scored | ELISA | −80℃ | NASH | No fibrosis: 26%, stage 1: 29%, stage 2: 20%, stage 3: 17% , stage 4: 8% | Pioglitazone or vitamin E 237（36.6%） |
| Alisi 2014 | [2] | CCL2 | Plasma | Italy | 7 | Liver biopsy specimens scored | ELISA | −80℃ | NAFL | Steatosis grade (1/2/3): 38(34.5%)/53(48.2%)/19(17.3%) | NA |
| Ando 2018 | [3] | CCL2, CXCL12 | Serum | Japan | 8 | Liver biopsy specimens scored | Fluorescent beads-based immunoassay | NR | NASH | Steatosis grade (1/2/3): Early NASH 4/15/5，Advanced NASH 4/5/0；Fibrosis stage (1/2/3/4):Early NASH 14/10/0/0，Advanced NASH 0/0/3/6 | NA |
| Baltieri 2018 | [4] | CXCL8 | Plasma | Brazil | 5 | Liver biopsy specimens scored | Western-blot analysis | NR | NASH | Mild steatosis 19 (100%); Fibrosis (absent/mild/moderate)3 (15.8%)/12 (63.1%)/4 (21.1%); Steatohepatitis (absent/mild/moderate)7 (36.8%)/10 (52.6%)/2 (10.6%) | NA |
| Barchetta 2018 | [5] | CXCL8 | Serum | Italy | 8 | Magnetic resonance | ELISA | NR | NAFL | NA | 24-week oral high-dose vitamin D supplementation |
| Chang 2015 | [6] | CCL2, CXCL10 | Plasma | China | 7 | Liver biopsy specimens scored | Highly sensitive magnetic bead-based assay | −70℃ | NAFL | NA | NA |
| Chu 2018 | [7] | CCL20 | Serum | USA | 6 | Liver biopsy specimens scored | ELISA | NR | NASH | Fibrosis 73（grade1/2/3/4/:18/18/28/13) | NA |
| Dinu 2018 | [8] | CCL2, CCL4, CXCL8, CXCL10 | Plasma | Italy | 8 | Ultrasonography | Bio-Plex cytokine assay | −80℃ | NAFL | Steatosis: graded 1 (mild steatosis) 28(70%), graded 2 (moderate steatosis) 12(30%) | NA |
| Duplessis 2015 | [9] | CCL2, CCL3, CCL13, CCL22, CXCL8 | Plasma | Belgium | 8 | Liver biopsy specimens scored | Custom Meso Scale Discovery V-plex assays | −80℃ | NASH | NA | NA |
| Fitzpatrick 2012 | [10] | CCL2 | Plasma | UK | 7 | Liver biopsy specimens scored | ELISA | −80℃ | NASH | Inflammation(minimal/mild/moderate):7/25/8; Ballooning 36 (90%);Fibrosis(0/F1/F2/F3): 1(2.5%)/11(27.5%)/15(37.5%)/13 (32.5%) | NA |
| Haukeland 2006 | [11] | CCL2, CCL19, CCL21 | Serum | Norway | 5 | Liver biopsy specimens scored | ELISA | −80℃ | NASH | In 25 NASH, inflammation(mild/moderate):19/6, Fibrosis stage (0/1/2/3/4):8/13/2/1/1; Among 22 simple steatosis: 2 stage 1 fibrosis | NA |
| Jamal 2017 | [12] | CXCL8 | Serum | Iran | 8 | Ultrasonography | ELISA | NR | NAFL | Steatosis grade(less than 33%/33%~66%/more than 66%):66.7%/18.5%/14.8%; Fibrosis degree (perisinusoidal/periportal/bridging/cirrhosis): 55.6%/33.3%/9.3%/1.9% | NA |
| Jamali 2016 | [13] | CXCL8 | Serum | Iran | 8 | Liver biopsy specimens scored | ELISA | NR | NAFL | Steatosis grade(less than 33%/33%~66%/more than 66%):66.7%/18.5%/14.8%;Fibrosis stage(1/2/3/4): 55.6%/33.3%/9.3%/1.9% | NA |
| Jarrar 2007 | [14] | CXCL8 | Serum | USA | 6 | Liver biopsy specimens scored | ELISA | NR | NAFL | NA | NA |
| Jiang 2018 | [15] | CXCL16 | Serum | China | 5 | Liver biopsy specimens scored | ELISA | 4℃ | NAFL | NA | NA |
| Kobyliak 2017 | [16] | CXCL8 | Serum | Ukraine | 5 | Ultrasonography | ELISA | NR | NAFL | NA | NA |
| Kugelmas 2003 | [17] | CXCL8 | Serum | USA | 5 | Liver biopsy specimens scored | ELISA | −70℃ | NASH | NA | A step 1 American Heart Association diet plus aerobic exercise with or without 800 IU of vitamin E daily |
| Kukla 2010 | [18] | Chemerin | Serum | Poland | 6 | Liver biopsy specimens scored | ELISA | −70℃ | NAFL | Steatosis (G0/1/2/3):41 (1/14/12/14); Fibrosis (F1A/1B/1C/2/3–4): 13(32%) (7/6/2/1/0) | NA |
| Kumar 2013 | [19] | CXCL8 | Serum | India | 5 | Liver biopsy specimens scored | ELISA | −80℃ | NAFL | Fibrosis stage(no/I/II/III/IV):12(63.16%)/ 4 (21.05%)/2 (10.53%)/ 1(5.26%)/0 | NA |
| Louthan 2005 | [20] | CXCL8 | Serum | USA | 5 | Liver biopsy specimens scored | ELISA | −70℃ | NAFL | NA | NA |
| Musso 2017 | [21] | CCL2 | Plasma | Italy | 8 | Ultrasonography | ELISA | −80℃ | NAFL | NA | NA |
| Page 2013 | [22] | CCL2 | Serum | USA | 7 | Liver biopsy specimens scored | ELISA | NR | NASH | Fibrosis: NASH 22 (100%), No NASH 10 (67%) | NA |
| Parker 2015 | [23] | CCL2 | Serum | USA | 5 | Liver biopsy specimens scored | ELISA | NR | NAFL | NA | NA |
| Perito 2017 | [24] | CXCL8 | Plasma | USA | 8 | Liver biopsy specimens scored | ELISA | −80℃ | NASH | Steatosis grade(0/1/2/3) (less than 5%/5%-33%/34%~66%/more than 66%):2%/26%/32%/40%; Fibrosis stage (0/1/2/3/4): 32%/38%/15%/14%/1% | NA |
| Plessis 2016 | [25] | CCL2, CCL3, CXCL8 | Plasma | Belgium | 8 | Liver biopsy specimens scored | ELISA | −80℃ | NASH | NA | NA |
| Polat 2013 | [26] | CCL2 | Serum | USA | 5 | Liver biopsy specimens scored | ELISA | NR | NAFL | NA | NA |
| Polyzos 2014 | [27] | Chemerin | Serum | Greece | 8 | Liver biopsy specimens scored | ELISA | −30℃ | NASH | Steatosis grade(<=33%/>33%):20/11;Fibrosis stage (absent/present):10/21 | NA |
| Ponziani 2019 | [28] | CCL2, CCL3, CCL4, CCL5, CCL11,CXCL8,CXCL10 | Plasma | Italy | 5 | Liver biopsy specimens scored | ELISA | NR | NAFL | NA | NA |
| Qi 2017 | [29] | CXCL8 | Serum | China | 6 | Liver biopsy specimens scored | High performance liquid chromatography-mass spectrometry | 4℃ | NASH | NA | NA |
| Serhal 2016 | [30] | CCL20 | Serum | Lebanon | 5 | Liver biopsy specimens scored | ELISA | 4℃ | NASH | NA | NA |
| Shanab 2011 | [31] | CXCL8 | Plasma | Ireland | 5 | Liver biopsy specimens scored | ELISA | −80℃ | NASH | NA | NA |
| Shoji 2016 | [32] | CCL20 | Serum | Japan | 4 | Liver biopsy specimens scored | ELISA | NR | NASH | Fibrosis(Stage 0–1/Stage 2/Stage 3/Stage 4):80/46/41/30 | NA |
| Tang 2014 | [33] | CXCL8 | Plasma | China | 4 | Ultrasonography | ELISA | −20℃ | NAFL | NA | NA |
| Tarantino 2014 | [34] | CCL11 | Serum | USA | 6 | Ultrasonography | ELISA | NR | NAFL | NA | NA |
| Torer 2007 | [35] | CXCL8 | Serum | Turkey | 6 | Ultrasonography | ELISA | −80℃ | NASH | NA | NA |
| Tuncer 2003 | [36] | CXCL8 | Serum | Turkey | 4 | Ultrasonography | Chemiluminescent immunometric method | NR | NAFL | NA | NA |
| Uysal 2011 | [37] | CXCL8 | Serum | Turkey | 5 | Ultrasonography | ELISA | 4℃ | NASH | NA | NA |
| Wada 2017 | [38] | CCL2, CCL3, CCL4,CXCL8,CXCL10 | Serum | Japan | 7 | Liver biopsy specimens scored | ELISA | −30℃ | NASH | NA | NA |
| Yang 2017 | [39] | CXCL8 | Serum | China | 7 | Ultrasonography | ELISA | NR | NAFL | 219 NAFLD patients(mild steatosis/moderate steatosis/severe steatosis : 91/102/26)(no advanced fibrosis/indeterminate liver fibrosis/advanced liver fibrosis: 76/110/33) | NA |
| Ye 2014 | [40] | Chemerin | Serum | China | 8 | Ultrasonography | ELISA | −80℃ | NAFL | NA | NA |
| Yilmaz 2010 | [41] | Chemerin | Serum | Turkey | 7 | Ultrasonography | ELISA | −80℃ | NAFL | NA | NA |
| Yilmaz 2011 | [42] | Chemerin | Serum | Turkey | 7 | Ultrasonography | ELISA | −80℃ | NAFL | NA | NA |
| Youness 2018 | [43] | CCL2 | Serum | Egypt | 6 | Ultrasonography | ELISA | −80℃ | NAFL | Fibrosis Stage 0/1/2/3: 5/7/12/7 | NA |
| Younossi 2008 | [44] | CXCL8 | Serum | USA | 6 | Ultrasonography | ELISA | NR | NASH | NA | NA |
| Zhang 2014 | [45] | CXCL10 | Serum | China | 5 | Ultrasonography | ELISA | NR | NASH | NA | NA |
| Zhu 2016 | [46] | CCL2, CXCL8 | Serum | China | 8 | Ultrasonography | ELISA | NR | NAFL | NA | NA |

**Appendix 2b: Characteristics** of included studies

| **Study** |  | **Male, n(%)** | **BMI** | **Mean Age** | **AST(IU/mL)** | **ALT(IU/mL)** | **GGT(IU/mL)** | **TG(mg/dL）** | **LDL-C(mg/dL）** | **HDL-C(mg/dL）** | **HOMA-IR** |
| --- | --- | --- | --- | --- | --- | --- | --- | --- | --- | --- | --- |
| Ajmera 2017 | [1] | 126(34) | 34.50 | 47.90 | 66.70 | 90.40 | NR | 194.00 | NR | 42.60 | 6.70 |
| Alisi 2014 | [2] | 64(58) | 25.60 | 11.00 | 48.00 | 67.00 | 23.00 | 90.00 | NR | NR | 2.10 |
| Ando 2018 | [3] | 24(73) | 28.50 | 46.10 | 54.50 | 89.70 | 62.00 | 208.60 | 132.00 | 46.40 | NR |
| Baltieri 2018 | [4] | 0(0) | 36.20 | 37.30 | NR | NR | NR | NR | NR | NR | NR |
| Barchetta 2018 | [5] | 35(72) | 30.50 | 56.20 | 26.90 | 39.60 | 51.20 | 135.00 | 100.70 | 48.40 | 4.40 |
| Chang 2015 | [6] | 27(56) | 25.05 | 53.50 | 27.00 | 29.00 | NR | 146.00 | 132.53 | 43.00 | 1.60 |
| Chu 2018 | [7] | 19(25) | 45.90 | 56.00 | 43.00 | 77.00 | NR | 165.00 | NR | 41.00 | NR |
| Dinu 2018 | [8] | 12(30) | 28.80 | 55.20 | 25.80 | 40.00 | 31.40 | 139.20 | 145.30 | 55.30 | NR |
| Duplessis 2015 | [9] | 24(31) | 44.00 | 43.00 | 25.00 | 31.00 | 28.00 | 144.00 | 121.60 | 45.60 | NR |
| Fitzpatrick 2012 | [10] | 30(75) | NR | 13.40 | 43.00 | 67.00 | 30.00 | 157.50 | NR | NR | 4.08 |
| Haukeland 2006 | [11] | 15(60) | 31.60 | 45.30 | NR | NR | NR | NR | NR | NR | NR |
| Jamal 2017 | [12] | 35(65) | 30.55 | 37.02 | 42.18 | 65.91 | 54.12 | 150.09 | 100.89 | 48.51 | NR |
| Jamali 2016 | [13] | 17(61) | 29.92 | 35.00 | 42.18 | 49.67 | 55.40 | 167.57 | 103.15 | 48.66 | NR |
| Jarrar 2007 | [14] | 11(42) | 47.50 | 43.90 | 35.10 | 45.96 | NR | 191.00 | NR | NR | NR |
| Jiang 2018 | [15] | 61(52) | 33.00 | 53.00 | NR | NR | NR | NR | NR | NR | NR |
| Kobyliak 2017 | [16] | 58(37) | 37.00 | 68.00 | 40.81 | 42.10 | NR | NR | NR | NR | NR |
| Kugelmas 2003 | [17] | 0(0) | 34.37 | 54.80 | 42.86 | 56.57 | NR | NR | NR | NR | NR |
| Kukla 2010 | [18] | 10(24) | 30.40 | 45.70 | 52.80 | 88.00 | 102.30 | 180.60 | NR | 42.60 | 3.50 |
| Kumar 2013 | [19] | 15(60) | 26.71 | 38.60 | 74.16 | 93.36 | NR | NR | NR | NR | NR |
| Louthan 2005 | [20] | 5(71) | NR | 13.40 | NR | 68.60 | NR | NR | NR | NR | NR |
| Musso 2017 | [21] | 46(61) | 25.70 | 49.00 | 17.00 | 18.00 | 20.00 | 112.00 | 100.00 | 61.00 | 1.70 |
| Page 2013 | [22] | 6(27) | 49.00 | 49.00 | 23.00 | 35.00 | NR | 179.00 | NR | 47.00 | NR |
| Parker 2015 | [23] | 29(51) | 35.00 | 56.00 | NR | NR | NR | NR | NR | NR | NR |
| Perito 2017 | [24] | 56(77) | NR | 13.50 | 83.60 | 143.50 | NR | 170.40 | NR | 35.60 | 10.50 |
| Plessis 2016 | [25] | 22(54) | 42.00 | 44.00 | 33.00 | 30.00 | 39.00 | 126.00 | 117.80 | 53.20 | 2.80 |
| Polat 2013 | [26] | 30(50) | 32.00 | 52.00 | NR | NR | NR | NR | NR | NR | NR |
| Polyzos 2014 | [27] | 8(26) | 34.10 | 53.90 | 49.00 | 71.00 | 64.00 | 199.00 | 135.00 | 47.00 | 5.78 |
| Ponziani 2019 | [28] | 9(45) | 26.00 | 60.00 | 21.50 | 18.50 | NR | NR | NR | NR | NR |
| Qi 2017 | [29] | 15(60) | 47.50 | 56.30 | 50.70 | 65.70 | 152.00 | 198.00 | NR | NR | NR |
| Serhal 2016 | [30] | 30(77) | NR | 38.60 | 88.74 | 115.75 | NR | NR | NR | NR | NR |
| Shanab 2011 | [31] | 8(10) | 30.00 | 51.17 | NR | 87.68 | 261.30 | NR | NR | NR | NR |
| Shoji 2016 | [32] | 47(58) | 27.50 | 50.00 | 41.00 | 75.00 | NR | NR | NR | NR | NR |
| Tang 2014 | [33] | 18(60) | 31.20 | 36.20 | NR | NR | NR | NR | NR | NR | NR |
| Tarantino 2014 | [34] | 36(45) | 42.30 | 46.00 | NR | 28.00 | 25.00 | 123.50 | NR | 49.30 | 2.78 |
| Torer 2007 | [35] | 62(67) | 29.40 | 45.30 | 44.60 | 75.50 | 74.10 | 178.00 | NR | NR | NR |
| Tuncer 2003 | [36] | 18(60) | 29.50 | 50.20 | NR | 81.00 | 68.00 | NR | NR | NR | NR |
| Uysal 2011 | [37] | 23(38) | 33.00 | 48.00 | NR | 45.40 | NR | NR | NR | NR | NR |
| Wada 2017 | [38] | 35(44) | 27.40 | 57.00 | 61.00 | 75.00 | 85.00 | 152.00 | NR | 49.00 | 3.60 |
| Yang 2017 | [39] | 139(63) | 26.16 | 47.27 | 37.89 | 47.30 | 67.25 | 216.90 | 112.50 | 46.74 | NR |
| Ye 2014 | [40] | 156(36) | 26.60 | 61.10 | 29.70 | 35.30 | 33.70 | 207.00 | 121.60 | 45.60 | 2.30 |
| Yilmaz 2010 | [41] | 50(50) | 30.60 | 48.00 | 44.00 | 68.00 | NR | 185.00 | 148.00 | 45.00 | 3.70 |
| Yilmaz 2011 | [42] | 26(48) | 31.20 | 47.00 | 44.00 | 68.00 | NR | 181.00 | 149.00 | 44.00 | 3.70 |
| Youness 2018 | [43] | 19(59) | 30.32 | 46.50 | 49.45 | 62.12 | 60.08 | 183.12 | NR | NR | NR |
| Younossi 2008 | [44] | 9(41) | 48.20 | 42.50 | 35.90 | 47.90 | NR | 177.80 | NR | NR | 3.40 |
| Zhang 2014 | [45] | 93(63) | 35.00 | 56.00 | NR | NR | NR | NR | NR | NR | NR |
| Zhu 2016 | [46] | 57(66) | 26.16 | 53.00 | 21.00 | 23.00 | 34.00 | 140.40 | 99.94 | 41.04 | 3.86 |

Appendix 2c: Original data of included studies

|  |  | **NAFLD** |  |  | **Control** | |  |
| --- | --- | --- | --- | --- | --- | --- | --- |
| **Study** |  | **N** | **Mean** | **SD** | **N** | **Mean** | **SD** |
| Ajmera 2017 | CXCL8 | 376 | 4.70 | 4.30 | 272 | 3.70 | 5.10 |
| Ajmera 2017 | CCL2 | 376 | 254.40 | 98.00 | 272 | 247.30 | 79.80 |
| Alisi 2014 | CCL2 | 110 | 170.00 | 53.00 | 40 | 102.00 | 25.00 |
| Ando 2018A | CXCL12 | 24 | 230.20 | 182.40 | 14 | 133.40 | 45.60 |
| Ando 2018B | CXCL12 | 9 | 371.30 | 227.50 | 14 | 133.40 | 45.60 |
| Ando 2018A | CCL2 | 24 | 55.10 | 22.20 | 14 | 35.20 | 10.40 |
| Ando 2018B | CCL2 | 9 | 54.20 | 23.60 | 14 | 35.20 | 10.40 |
| Baltieri 2018 | CXCL8 | 12 | 15.60 | 9.30 | 7 | 12.60 | 5.70 |
| Barchetta 2018 | CXCL8 | 35 | 78.60 | 125.30 | 27 | 67.90 | 121.40 |
| Chang 2015 | CXCL10 | 48 | 369.44 | 235.23 | 43 | 293.37 | 135.65 |
| Chang 2015 | CCL2 | 48 | 297.56 | 156.25 | 43 | 239.63 | 158.71 |
| Chu 2018 | CCL20 | 77 | 29.30 | 13.70 | 106 | 2.20 | 1.30 |
| Dinu 2018 | CXCL8 | 20 | 9.52 | 5.09 | 20 | 6.18 | 2.78 |
| Dinu 2018 | CXCL10 | 20 | 984.70 | 774.50 | 20 | 901.70 | 514.30 |
| Dinu 2018 | CCL2 | 20 | 41.80 | 25.10 | 20 | 44.70 | 44.40 |
| Dinu 2018 | CCL4 | 20 | 71.90 | 40.50 | 20 | 90.20 | 44.80 |
| Duplessis 2015A | CXCL8 | 20 | 1.40 | 0.60 | 17 | 1.60 | 0.80 |
| Duplessis 2015B | CXCL8 | 24 | 1.70 | 0.70 | 17 | 1.60 | 0.80 |
| Duplessis 2015A | CCL2 | 20 | 182.00 | 97.00 | 17 | 163.00 | 102.00 |
| Duplessis 2015B | CCL2 | 24 | 191.00 | 113.00 | 17 | 163.00 | 102.00 |
| Duplessis 2015A | CCL13 | 20 | 6.70 | 3.20 | 17 | 5.70 | 3.80 |
| Duplessis 2015B | CCL13 | 24 | 8.70 | 5.10 | 17 | 5.70 | 3.80 |
| Duplessis 2015A | CCL22 | 20 | 715.00 | 217.00 | 17 | 634.00 | 239.00 |
| Duplessis 2015B | CCL22 | 24 | 673.00 | 199.00 | 17 | 634.00 | 239.00 |
| Duplessis 2015A | CCL3 | 20 | 5.90 | 2.70 | 17 | 6.50 | 3.30 |
| Duplessis 2015B | CCL3 | 24 | 6.70 | 3.10 | 17 | 6.50 | 3.30 |
| Fitzpatrick 2012 | CCL2 | 28 | 201.00 | 139.00 | 12 | 199.00 | 136.00 |
| Haukeland 2006A | CCL2 | 22 | 137.00 | 153.00 | 30 | 85.00 | 100.00 |
| Haukeland 2006B | CCL2 | 25 | 199.00 | 92.00 | 30 | 85.00 | 100.00 |
| Haukeland 2006A | CCL19 | 22 | 275.00 | 278.00 | 30 | 90.00 | 136.00 |
| Haukeland 2006B | CCL19 | 25 | 355.00 | 358.00 | 30 | 90.00 | 136.00 |
| Haukeland 2006A | CCL21 | 22 | 415.00 | 177.00 | 30 | 392.00 | 121.00 |
| Haukeland 2006B | CCL21 | 25 | 415.00 | 227.00 | 30 | 392.00 | 121.00 |
| Jamal 2017 | CXCL8 | 54 | 27.41 | 24.99 | 54 | 15.59 | 12.42 |
| Jamali 2016 | CXCL8 | 28 | 38.60 | 28.21 | 24 | 25.51 | 13.11 |
| Jarrar 2007 | CXCL8 | 45 | 24.10 | 38.50 | 38 | 7.80 | 3.60 |
| Jiang 2018 | CXCL16 | 117 | 139.00 | 89.00 | 15 | 101.00 | 25.00 |
| Kobyliak 2017 | CXCL8 | 157 | 29.18 | 1.27 | 88 | 22.05 | 0.99 |
| Kugelmas 2003 | CXCL8 | 16 | 11.50 | 3.70 | 18 | 8.10 | 2.50 |
| Kukla 2010 | Chemerin | 41 | 24.70 | 17.10 | 10 | 6.10 | 2.50 |
| Kumar 2013 | CXCL8 | 25 | 282.26 | 182.53 | 25 | 37.32 | 35.22 |
| Louthan 2005 | CXCL8 | 7 | 0.70 | 0.10 | 12 | 0.50 | 0.10 |
| Louthan 2005 | CXCL8 | 7 | 0.70 | 0.10 | 11 | 0.70 | 0.20 |
| Musso 2017 | CCL2 | 69 | 144.00 | 17.00 | 69 | 123.00 | 21.00 |
| Page 2013 | CCL2 | 22 | 464.00 | 118.00 | 15 | 486.00 | 218.00 |
| Parker 2015 | CCL2 | 56 | 330.00 | 157.20 | 56 | 207.00 | 90.60 |
| Perito 2017 | CXCL8 | 73 | 3.40 | 1.50 | 162 | 2.90 | 2.20 |
| Perito 2017 | CCL2 | 73 | 245.70 | 90.00 | 162 | 246.90 | 105.30 |
| Plessis 2016A | CCL2 | 41 | 118.00 | 52.00 | 34 | 143.00 | 72.00 |
| Plessis 2016A | CCL3 | 41 | 7.40 | 3.10 | 34 | 6.70 | 3.50 |
| Plessis 2016A | CXCL8 | 41 | 1.80 | 0.70 | 34 | 1.70 | 0.50 |
| Plessis 2016B | CCL2 | 16 | 140.00 | 55.00 | 34 | 143.00 | 72.00 |
| Plessis 2016B | CCL3 | 16 | 9.30 | 3.80 | 34 | 6.70 | 3.50 |
| Plessis 2016B | CXCL8 | 16 | 3.30 | 1.10 | 34 | 1.70 | 0.50 |
| Polat 2013 | CCL2 | 60 | 190.73 | 87.56 | 20 | 165.75 | 59.63 |
| Polyzos 2014A | Chemerin | 15 | 12.90 | 0.70 | 24 | 11.80 | 0.60 |
| Polyzos 2014B | Chemerin | 16 | 11.30 | 0.70 | 28 | 13.30 | 0.10 |
| Ponziani 2019 | CXCL8 | 20 | 7.10 | 0.35 | 20 | 7.10 | 0.77 |
| Ponziani 2019 | CCL2 | 20 | 19.07 | 2.04 | 20 | 13.97 | 3.08 |
| Ponziani 2019 | CCL3 | 20 | 1.50 | 0.14 | 20 | 1.50 | 0.21 |
| Ponziani 2019 | CCL4 | 20 | 104.17 | 4.71 | 20 | 112.19 | 5.61 |
| Ponziani 2019 | CCL5 | 20 | 4201.14 | 1449.48 | 20 | 2990.37 | 893.29 |
| Ponziani 2019 | CCL11 | 20 | 71.49 | 29.94 | 20 | 67.19 | 30.10 |
| Ponziani 2019 | CXCL10 | 20 | 557.62 | 130.02 | 20 | 355.23 | 169.60 |
| Qi 2017 | CXCL8 | 25 | 17.73 | 8.70 | 25 | 25.15 | 13.87 |
| Serhal 2016 | CCL20 | 39 | 58.60 | 21.70 | 37 | 15.30 | 5.50 |
| Shanab 2011 | CXCL8 | 18 | 18.14 | 4.30 | 16 | 8.24 | 0.82 |
| Shoji 2016 | CCL20 | 117 | 10.70 | 7.30 | 80 | 5.30 | 3.60 |
| Tang 2014 | CXCL8 | 30 | 73.09 | 20.38 | 30 | 31.42 | 6.13 |
| Tarantino 2014 | CCL11 | 80 | 24.60 | 6.30 | 80 | 19.40 | 7.20 |
| Torer 2007 | CXCL8 | 57 | 104.21 | 36.31 | 35 | 47.53 | 21.21 |
| Tuncer 2003 | CXCL8 | 30 | 43.68 | 40.20 | 30 | 9.51 | 1.20 |
| Uysal 2011 | CXCL8 | 60 | 26.36 | 9.18 | 28 | 11.69 | 4.80 |
| Wada 2017A | CXCL8 | 20 | 210.00 | 55.00 | 15 | 63.00 | 17.00 |
| Wada 2017A | CCL3 | 20 | 109.00 | 97.00 | 15 | 11.00 | 5.00 |
| Wada 2017A | CCL4 | 20 | 1130.00 | 560.00 | 15 | 107.00 | 77.00 |
| Wada 2017A | CCL2 | 20 | 89.00 | 63.00 | 15 | 83.00 | 52.00 |
| Wada 2017A | CXCL10 | 20 | 810.00 | 230.00 | 15 | 510.00 | 170.00 |
| Wada 2017B | CXCL8 | 59 | 153.00 | 69.00 | 15 | 63.00 | 17.00 |
| Wada 2017B | CCL3 | 59 | 51.00 | 33.00 | 15 | 11.00 | 5.00 |
| Wada 2017B | CCL4 | 59 | 670.00 | 390.00 | 15 | 107.00 | 77.00 |
| Wada 2017B | CCL2 | 59 | 118.00 | 77.00 | 15 | 83.00 | 52.00 |
| Wada 2017B | CXCL10 | 59 | 1270.00 | 650.00 | 15 | 510.00 | 170.00 |
| Yang 2017 | CXCL8 | 219 | 1126.98 | 92.95 | 166 | 361.80 | 42.38 |
| Ye 2014 | Chemerin | 436 | 77.84 | 4.37 | 467 | 85.91 | 4.80 |
| Yilmaz 2010 | Chemerin | 99 | 219.00 | 83.00 | 75 | 159.00 | 43.00 |
| Yilmaz 2011 | Chemerin | 54 | 235.00 | 84.00 | 56 | 151.00 | 37.00 |
| Youness 2018 | CCL2 | 22 | 19.28 | 1.00 | 24 | 7.25 | 0.91 |
| Younossi 2008 | CXCL8 | 22 | 24.00 | 29.40 | 32 | 7.30 | 3.10 |
| Zhang 2014 | CXCL10 | 69 | 248.00 | 59.00 | 73 | 111.00 | 33.00 |
| Zhu 2016 | CXCL8 | 86 | 7.82 | 3.22 | 86 | 6.57 | 2.79 |
| Zhu 2016 | CCL2 | 86 | 271.15 | 98.45 | 86 | 307.84 | 106.91 |

**Appendix 3a: Local inconsistency for the network of Chemokines in NAFL** group (node-splitting method)

| **Side** |  | **Direct** |  | **Indirect** |  | **Difference** |  |  |  |
| --- | --- | --- | --- | --- | --- | --- | --- | --- | --- |
|  |  | **Coef.** | **Std.Err.** | **Coef.** | **Std.Err.** | **Coef.** | **Std.Err.** | **P>|z|** | **tau** |
| A | B | 1.258639 | .6837742 | 4.133276 | 2.222066 | -2.874637 | 2.324908 | 0.216 | 2.323388 |
| A | C | .6539906 | 1.373931 | 3.194207 | 2.077406 | -2.540216 | 2.491423 | 0.308 | 2.355247 |
| A | D | .5579007 | 1.37624 | 2.943128 | 2.084393 | -2.385227 | 2.498086 | 0.340 | 2.359791 |
| A | E | 1.322425 | 2.403633 | 3.25055 | 3.037005 | -1.928126 | 3.872928 | 0.619 | 2.38151 |
| A | F | .4776367 | 1.693887 | 2.201012 | 3.101461 | -1.723375 | 3.533982 | 0.626 | 2.382277 |
| A | G | .3491404 | 2.382974 | 4.077913 | 3.297265 | -3.728772 | 4.070214 | 0.360 | 2.359911 |
| A | H | 1.184783 | 2.400601 | 3.700365 | 4.385766 | -2.515582 | 4.996732 | 0.615 | 2.382503 |
| A | I | .0272364 | 2.398982 | 2.542818 | 4.385916 | -2.515582 | 4.996733 | 0.615 | 2.382503 |
| A | J | .249386 | 2.382916 | 3.978158 | 3.297206 | -3.728772 | 4.070214 | 0.360 | 2.359911 |
| A | K | 1.847128 | .6206464 | 3.500581 | 2.367663 | -1.653453 | 2.447624 | 0.499 | 2.38153 |
| A | L | .7123366 | 1.180852 | 3.544593 | 2.002614 | -2.832257 | 2.325075 | 0.223 | 2.341684 |
| B | C | .1763846 | 1.39193 | -.8553204 | 2.388015 | 1.031705 | 2.763818 | 0.709 | 2.388163 |
| B | D | .1905936 | 1.388314 | -1.468675 | 2.382479 | 1.659268 | 2.757235 | 0.547 | 2.381658 |
| B | E | .1400678 | 2.406051 | 1.262499 | 3.168558 | -1.122431 | 3.97834 | 0.778 | 2.385166 |
| B | F | -.9966795 | 2.409005 | -.3824907 | 2.054832 | -.6141888 | 3.166524 | 0.846 | 2.387463 |
| B | G | .1122109 | 2.410644 | .1227316 | 3.459505 | -.0105207 | 4.216454 | 0.998 | 2.389801 |
| B | H | .8307101 | 2.402302 | -1.684871 | 4.382972 | 2.515582 | 4.996733 | 0.615 | 2.382503 |
| B | I | -.3268368 | 2.401629 | -2.842418 | 4.381567 | 2.515582 | 4.996732 | 0.615 | 2.382503 |
| B | J | .0124679 | 2.410633 | .0229886 | 3.459396 | -.0105207 | 4.216454 | 0.998 | 2.389801 |
| B | K | .2440935 | 1.073994 | .6751226 | 1.181788 | -.4310291 | 1.596874 | 0.787 | 2.383102 |
| B | L | .3520855 | 1.196942 | -1.710972 | 2.370655 | 2.063058 | 2.655463 | 0.437 | 2.375805 |
| C | D | -.0876928 | 1.704508 | -.2956301 | 2.749984 | .2079373 | 3.235411 | 0.949 | 2.389685 |
| C | E | 1.323698 | 2.400151 | -1.186857 | 3.90728 | 2.510555 | 4.585421 | 0.584 | 2.377996 |
| C | F | .1867208 | 2.403254 | -1.407879 | 2.575428 | 1.5946 | 3.522618 | 0.651 | 2.382334 |
| C | G | .5486427 | 2.407711 | -.8177977 | 4.123888 | 1.36644 | 4.775216 | 0.775 | 2.386565 |
| C | J | .4488895 | 2.407616 | -.9175509 | 4.123875 | 1.36644 | 4.775216 | 0.775 | 2.386565 |
| C | K | -.3207238 | 1.356632 | 2.895622 | 2.269391 | -3.216346 | 2.643459 | 0.224 | 2.328105 |
| C | L | .0868202 | 1.707007 | -.1150753 | 2.34711 | .2018956 | 2.902358 | 0.945 | 2.389657 |
| D | E | 1.557696 | 2.397596 | -1.240315 | 3.869338 | 2.798011 | 4.55225 | 0.539 | 2.374944 |
| D | F | .4202098 | 2.401438 | -1.355178 | 2.562201 | 1.775387 | 3.51185 | 0.613 | 2.380402 |
| D | K | .1189772 | 1.376798 | 2.233028 | 2.317282 | -2.114051 | 2.694989 | 0.433 | 2.362863 |
| D | L | .2762598 | 1.391721 | -1.043353 | 4.527636 | 1.319613 | 4.736564 | 0.781 | 2.386877 |
| E | F | -1.136439 | 2.411866 | -1.492178 | 5.627874 | .3557391 | 6.122568 | 0.954 | 2.390142 |
| E | K | -1.324097 | 2.383167 | 1.960833 | 3.114173 | -3.28493 | 3.921234 | 0.402 | 2.360853 |
| E | L | .8498462 | 2.361943 | -3.939402 | 3.552623 | 4.789248 | 4.268169 | 0.262 | 2.340164 |
| F | K | -.1867533 | 2.391826 | 1.975616 | 2.010926 | -2.162369 | 3.124923 | 0.489 | 2.370805 |
| F | L | 1.987573 | 2.385248 | -.8213702 | 2.366447 | 2.808943 | 3.361125 | 0.403 | 2.361402 |
| G | K | -.6111886 | 2.391714 | 2.207301 | 3.395359 | -2.81849 | 4.153148 | 0.497 | 2.370356 |
| J | K | -.5114027 | 2.391607 | 2.307087 | 3.395363 | -2.81849 | 4.153148 | 0.497 | 2.370356 |
| K | L | .1578786 | 1.377689 | -1.573264 | 1.742401 | 1.731143 | 2.22112 | 0.436 | 2.363534 |

A, Control; B, CCL2; C, CCL3; D, CCL4; E, CCL5; F, CCL11; G, CCL13; H, CCL19; I, CCL21; J, CCL22; K, CXCL8; L, CXCL10; M, CXCL16; N, Chemerin

**Appendix 3b: Local inconsistency for the network of Chemokines in NASH group (node-splitting method)**

| **Side** |  | **Direct** |  | **Indirect** |  | **Difference** |  |  |  |
| --- | --- | --- | --- | --- | --- | --- | --- | --- | --- |
|  |  | **Coef.** | **Std.Err.** | **Coef.** | **Std.Err.** | **Coef.** | **Std.Err.** | **P>|z|** | **tau** |
| A | B | .1612376 | .2401786 | 1.799146 | .7835301 | -1.637908 | .8204801 | **0.046** | .7418191 |
| A | C | .6319465 | .4152756 | 1.69559 | .712699 | -1.063644 | .8286993 | 0.199 | .7788074 |
| A | D | 1.891317 | .8572886 | 2.309912 | 1.133557 | -.4185955 | 1.454569 | 0.774 | .8033645 |
| A | E | .8183151 | .856158 | 2.056001 | 1.177471 | -1.237686 | 1.466425 | 0.399 | .7936752 |
| A | F | 1.425713 | .8527198 | .6692544 | 1.558145 | .7564588 | 1.771951 | 0.669 | .8022206 |
| A | H | .0228114 | .8466958 | -.7336474 | 1.560755 | .7564588 | 1.771951 | 0.669 | .8022206 |
| A | I | .1347813 | .8546818 | 1.372467 | 1.17705 | -1.237686 | 1.466425 | 0.399 | .7936752 |
| A | J | .8373271 | .2329424 | 1.918364 | .8879425 | -1.081037 | .9185765 | 0.239 | .7804311 |
| A | K | 1.770636 | .58406 | .3344917 | 1.141919 | 1.436144 | 1.301902 | 0.270 | .7818518 |
| A | L | 1.267945 | .6353183 | .4276496 | 1.207633 | .8402953 | 1.362784 | 0.537 | .7978254 |
| B | C | .6320531 | .4246276 | .4641154 | .8684285 | .1679378 | .9667472 | 0.862 | .8050458 |
| B | D | 1.733316 | .8297906 | 1.761149 | 1.173138 | -.0278333 | 1.435418 | 0.985 | .8060263 |
| B | E | .5496318 | .8456076 | 1.757258 | 1.21003 | -1.207626 | 1.474788 | 0.413 | .7940726 |
| B | F | .775588 | .8524014 | 1.532047 | 1.558667 | -.7564588 | 1.771951 | 0.669 | .8022206 |
| B | H | -.6273138 | .8517865 | .129145 | 1.552423 | -.7564588 | 1.771951 | 0.669 | .8022206 |
| B | I | -.1349973 | .8449587 | 1.072629 | 1.208603 | -1.207626 | 1.474788 | 0.413 | .7940726 |
| B | J | .5007037 | .3403854 | .8385313 | .5086937 | -.3378276 | .612048 | 0.581 | .7998672 |
| B | K | .4667648 | .8053217 | 1.714198 | .7236806 | -1.247434 | 1.082847 | 0.249 | .7837621 |
| B | L | .6079569 | .629449 | 1.448252 | 1.21684 | -.8402953 | 1.362784 | 0.537 | .7978254 |
| C | D | .4555546 | .7824285 | 2.932366 | 1.263753 | -2.476811 | 1.484979 | 0.095 | .760233 |
| C | E | .7709432 | .8420324 | -.7350453 | 1.340729 | 1.505988 | 1.582047 | 0.341 | .789548 |
| C | I | .0867311 | .8406833 | -1.419257 | 1.340179 | 1.505988 | 1.582047 | 0.341 | .789548 |
| C | J | -.0453895 | .4244293 | .2018115 | .8313899 | -.2472011 | .9341282 | 0.791 | .8043377 |
| C | K | -.8159445 | .7198263 | 2.030186 | .7499272 | -2.846131 | 1.04002 | **0.006** | .6950963 |
| D | J | -1.288562 | .8272853 | -.8372579 | 1.162401 | -.4513045 | 1.424505 | 0.751 | .8048319 |
| D | K | -1.264495 | .7421866 | 3.380419 | 1.792319 | -4.644913 | 1.938216 | **0.017** | .7171417 |
| E | J | -.7225441 | .8485691 | .429054 | 1.203877 | -1.151598 | 1.471555 | 0.434 | .7966903 |
| I | J | -.0385357 | .847381 | 1.113062 | 1.20309 | -1.151598 | 1.471555 | 0.434 | .7966903 |
| J | K | .0226875 | .8129661 | .9699095 | .7214211 | -.947222 | 1.086903 | 0.383 | .7918426 |

A, Control; B, CCL2; C, CCL3; D, CCL4; E, CCL13; F, CCL19; G, CCL20; H, CCL21; I, CCL22; J, CXCL8; K, CXCL10; L, CXCL12; M, Chemerin

**Appendix 4a: The classification of chemokines and their receptors.**

| **Subfamily of chemokine** | **Name of chemokine** | **Other names of chemokine** | **Receptors** |
| --- | --- | --- | --- |
| **C chemokines** | XCL1 | Limphotactin α, SCM-1α | XCR1 |
|  | XCL2 | Limphotactin β, SCM-1β | XCR1 |
| **CC chemokines** | CCL1 | I-309 | CCR8 |
|  | **CCL2** | MCP-1 | **CCR2** |
|  | **CCL3** | MIP-1α | **CCR1,CCR5** |
|  | **CCL4** | MIP-1β | **CCR5,CCR8** |
|  | CCL5 | RANTES | CCR1,CCR3,CCR5 |
|  | CCL6 | C-10,Mrp-1 | CCR1 |
|  | CCL7 | MCP-3 | CCR1,CCR2,CCR3 |
|  | CCL8 | MCP-2 | CCR1,CCR2,CCR3,CCR5,CCR8 |
|  | CCL9 | MIP-1γ,MRP2 | CCR1,CCR3 |
|  | CCL11 | Eotaxin-1 | CCR3,CCR5 |
|  | CCL12 | MCP-5 | CCR2 |
|  | CCL13 | MCP-4 | CCR1,CCR2，CCR3,CCR5 |
|  | CCL14 | HCC-1 | CCR1,CCR5 |
|  | CCL15 | HCC-2, Leukotactin-1, MIP-5 | CCR1,CCR3 |
|  | CCL16 | HCC-4, LEC, NCC-4, MTN1 | CCR1,CCR2,CCR5,CCR8 |
|  | CCL17 | TARC | CCR4 |
|  | CCL18 | MIP-4, AMAC1 | CCR8 |
|  | CCL19 | ELC,MIP-3β | CCR7 |
|  | **CCL20** | LARC,MIP-3α | **CCR6** |
|  | CCL21 | SLC,6Ckine | CCR7 |
|  | CCL22 | MDC | CCR4 |
|  | CCL23 | MPIF-1,MIP-3 | CCR1,CCR3 |
|  | CCL24 | MPIF-2,Eotaxin-2 | CCR3 |
|  | CCL25 | TECK | CCR9 |
|  | CCL26 | Eotaxin-3,MIP-4α | CCR3,CX3CR1 |
|  | CCL27 | CTAK | CCR10 |
|  | CCL28 | MEC | CCR3,CCR10 |
| **CXC chemokines** | CXCL1 | GROα,MGSA | CXCR2 |
|  | CXCL2 | GROβ,MIP-2α | CXCR2 |
|  | CXCL3 | GROγ,MIP-2β | CXCR2 |
|  | CXCL4 | PF-4 | CXCR3 |
|  | CXCL5 | ENA-78 | CXCR2 |
|  | CXCL6 | GCP-2 | CXCR1,CXCR2 |
|  | CXCL7 | NAP-2 | CXCR2 |
|  | **CXCL8** | IL-8 | **CXCR1,CXCR2** |
|  | CXCL9 | Mig | CXCR3 |
|  | **CXCL10** | IP-10 | **CXCR3** |
|  | CXCL11 | I-TAC | CXCR3,CXCR7 |
|  | CXCL12 | SDF-1 | CXCR4,CXCR7 |
|  | CXCL13 | BCA-1,BLC | CXCR5,CXCR3 |
|  | CXCL14 | BRAK | Unknown |
|  | CXCL15 | Lungkine | Unknown |
|  | CXCL16 | SRPSOX | CXCR6 |
| **CX3C chemokines** | CX3CL1 | Fractalkine | CX3CR1 |

**Appendix 4b: The distribution—cell type of chemokines receptors.**

| **Receptors of Chemokine** | **Distribution—Cell Type** |
| --- | --- |
| **CXCR1** | Neutrophils, monocytes, mast cells, basophils, dendric cells, CD8 T cells,natural killer cells |
| **CXCR2** | Neutrophils, monocytes, mast cells, basophils, dendric cells, natural killer cells |
| **CXCR3** | Basophils, Th1 cells, CD8 T cells, natural killer cells, Treg cells |
| **CXCR4** | Widely expressed |
| **CXCR5** | Basophils, CD8 T cells |
| **CXCR6** | Th1 cells, Th17 cells, natural killer cells, plasma cells |
| **CCR2** | Monocytes, macrophages, Th1 cells, basophil, natural killer cells |
| **CCR5** | Dendric cells, monocytes, macrophages, natural killer cells, Th1 cells, TH17 cells, |
| **CCR1** | Neutrophils, monocytes, macrophages, Th1 cells, basophils, dendric cells |
| **CCR3** | Eosinophils, basophils, Th2 cells, mast cells, dendric cells |
| **CCR4** | Th2 cells, Th17 cells, Treg cells, monocytes, basophils, CD4 & CD8 T cells |
| **CCR6** | Th17 cells, natural killer cells, Treg cells |
| **CCR7** | Dendric cells (mature), T cells, basophils |
| **CCR8** | Dendirc cells, monocytes, macrophages, Th2 cells, Treg cells |
| **CCR9** | Basophils, dendric cells |
| **CCR10** | T cells, IgA+ plasma cells |
| **XCR1** | Dendric cells |
| **CX3CR1** | Monocytes, macrophages, Th1 cells, dendric cells, natural killer cells |

**Appendix 4c: The suggested chemokines and chemokines receptors involvement in NAFLD.**

| **Suggested involvement in NAFLD** | **Chemokines/receptors of chemokines** |
| --- | --- |
| **Fibrosis** | CCL1 and CCR8 |
|  | CCL25 and CCR9 |
|  | CXCL11 |
|  | CXCL16 and CXCR6 |
|  | CX3CL1 |
| **Fibrosis, NASH** | CCL3 and CCR5 |
|  | CXCL9 and CXCR3 |
|  | CCL4 and CCR5 |
|  | CCL5 and CCR1, CCR5 |
|  | CCL20 and CCR6 |
|  | CXCL10 and CXCR3 |
|  | CXCL12 and CXCR4,CXCR7 |
|  | CCL2 and CCR2 |

**Appendix 4d:** The chemokine-binding profiles of the atypical chemokine receptors.

| **Atypical receptor** | **Chemokine ligands** | **Biological functions** |
| --- | --- | --- |
| **ACKR1 (DARC)** | CCL2, CCL5, CCL7, CCL11, CCL13, CCL14, CCL17, CXCL1, CXCL3, CXCL5, CXCL6, CXCL8, CXCL11 | Chemokine transporter |
| **ACKR2 (D6)** | CCL2, CCL3, CCL4, CCL5, CCL7, CCL8, CCL11, CCL13, CCL14, CCL17, CCL22 | Chemokine scavenger |
| **ACKR3 (CXCR7)** | CXCL11, CXCL12 | Chemokine scavenger |
| **ACKR4 (CCX-CKR)** | CCL19, CCL21, CCL25, CXCL13 | Chemokine scavenger |

**Appendix 5a:** **Rank probability of Chemokines in NAFL group (A) and Chemokines in NASH group (B) for response rate in the network analysis.**


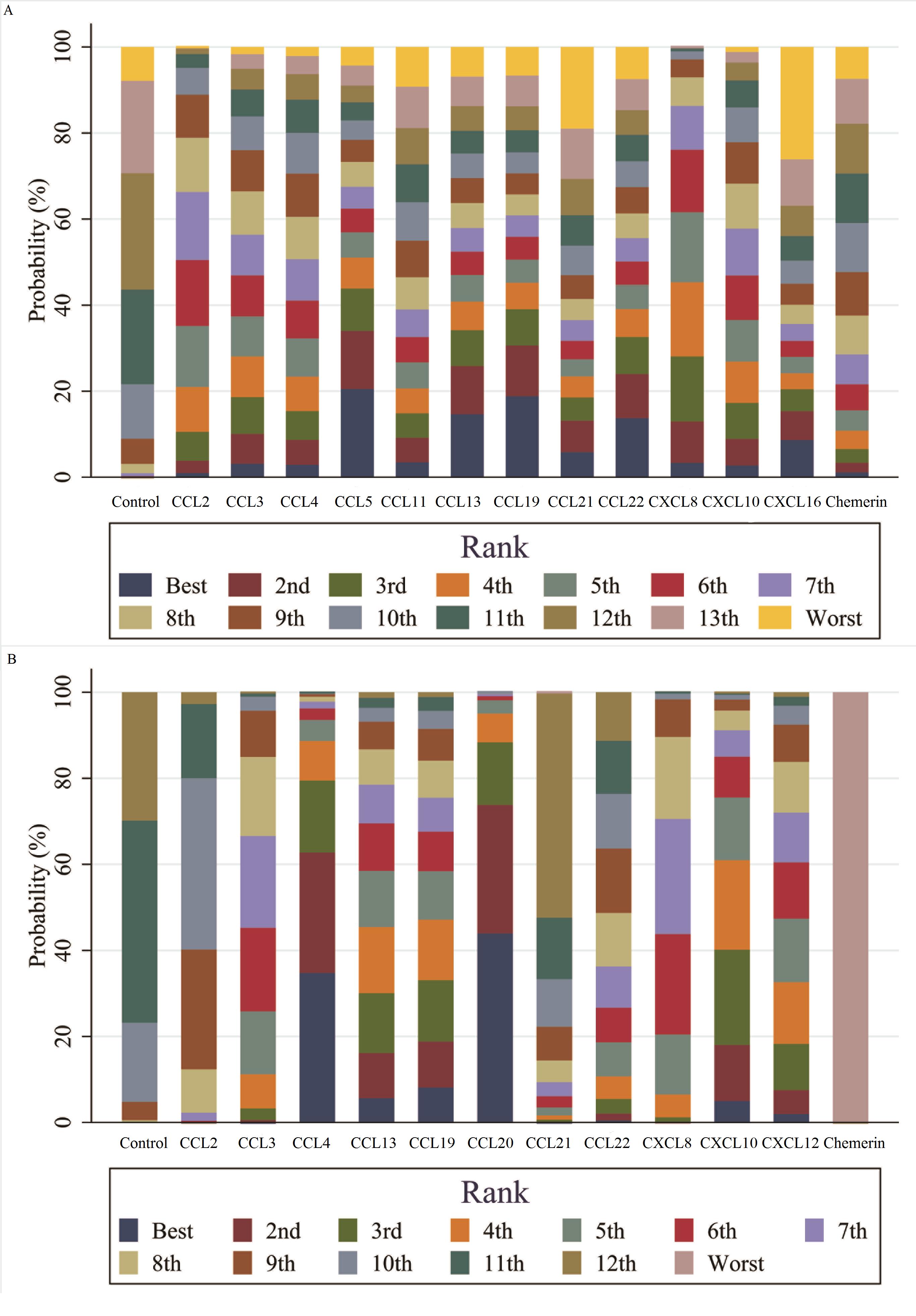


**Appendix 5b: Rank and SUCRA of the effect of different chemokines in NAFL** and NASH

| **NAFL** | **SUCRA** | **MeanRank** |
| --- | --- | --- |
| **CXCL8** | 69.4 | 5.0 |
| CCL5 | 65.0 | 5.6 |
| CCL19 | 59.5 | 6.3 |
| **CCL2** | 57.6 | 6.5 |
| CCL13 | 57.0 | 6.6 |
| CCL22 | 55.3 | 6.8 |
| CXCL10 | 55.1 | 6.8 |
| CCL3 | 54.6 | 6.9 |
| CCL4 | 51.1 | 7.4 |
| CCL11 | 42.8 | 8.4 |
| CCL21 | 39.2 | 8.9 |
| CXCL16 | 37.9 | 9.1 |
| Chemerin | 36.7 | 9.2 |
| Control | 18.6 | 11.6 |
| **NASH** |  |  |
| **CCL20** | 91.5 | 2.0 |
| **CCL4** | 87.6 | 2.5 |
| **CXCL10** | 72.4 | 4.3 |
| CCL13 | 64.9 | 5.2 |
| CCL19 | 64.9 | 5.2 |
| CXCL12 | 59.4 | 5.9 |
| **CCL3** | 52.7 | 6.7 |
| **CXCL8** | 52.5 | 6.7 |
| CCL22 | 39.8 | 8.2 |
| CCL2 | 27.7 | 9.7 |
| CCL21 | 19.9 | 10.6 |
| Control | 16.6 | 11.0 |
| Chemerin | 0.0 | 13.0 |

This table shows the relative cumulative probabilities for each of the chemokines in the network. SUCRA values are presented in the legend. SUCRA, surface under cumulative ranking curve.

**Appendix 6: Comparison-adjusted funnel plot for the network of Chemokines in NAFL group (A) and Chemokines in NASH group (B)**


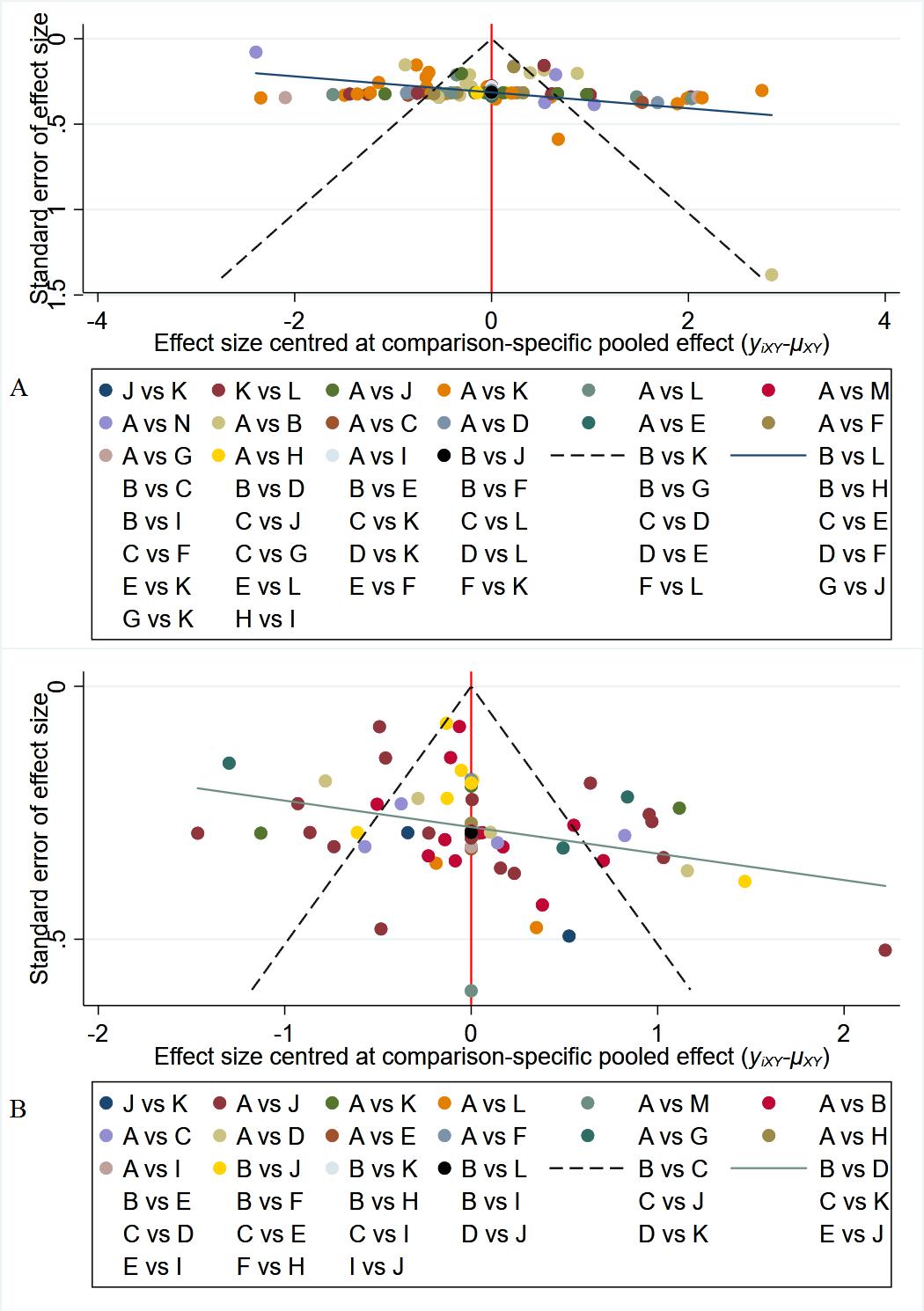


(A) A, Control; B, CCL2; C, CCL3; D, CCL4; E, CCL5; F, CCL11; G, CCL13; H, CCL19; I, CCL21; J, CCL22; K, CXCL8; L, CXCL10; M, CXCL16; N, Chemerin

(B) A, Control; B, CCL2; C, CCL3; D, CCL4; E, CCL13; F, CCL19; G, CCL20; H, CCL21; I, CCL22; J, CXCL8; K, CXCL10; L, CXCL12; M, Chemerin

**Appendix 7a:** Risk of bias assessments

| **Study** | **NOS** | **Selection** | | | | **Comparability** | | **Outcome** | | |
| --- | --- | --- | --- | --- | --- | --- | --- | --- | --- | --- |
|  |  | **1** | **2** | **3** | **4** | **5** | **6** | **7** | **8** | **9** |
| Ajmera 2017 | 7 | 1 | 1 | 1 | 1 | 1 | 0 | 1 | 1 | 0 |
| Alisi 2014 | 7 | 1 | 1 | 1 | 1 | 1 | 0 | 1 | 1 | 0 |
| Ando 2018 | 8 | 1 | 1 | 1 | 1 | 1 | 1 | 1 | 1 | 0 |
| Baltieri 2018 | 5 | 1 | 0 | 0 | 1 | 1 | 0 | 1 | 1 | 0 |
| Barchetta 2018 | 8 | 1 | 1 | 1 | 1 | 1 | 1 | 1 | 1 | 0 |
| Chang 2015 | 7 | 1 | 0 | 1 | 1 | 1 | 1 | 1 | 1 | 0 |
| Chu 2018 | 6 | 1 | 0 | 1 | 1 | 1 | 0 | 1 | 1 | 0 |
| Dinu 2018 | 8 | 1 | 1 | 1 | 1 | 1 | 1 | 1 | 1 | 0 |
| Duplessis 2015 | 8 | 1 | 1 | 1 | 1 | 1 | 1 | 1 | 1 | 0 |
| Fitzpatrick 2012 | 7 | 1 | 0 | 1 | 1 | 1 | 1 | 1 | 1 | 0 |
| Haukeland 2006 | 5 | 1 | 0 | 0 | 1 | 1 | 0 | 1 | 1 | 0 |
| Jamal 2017 | 8 | 1 | 1 | 1 | 1 | 1 | 1 | 1 | 1 | 0 |
| Jamali 2016 | 8 | 1 | 1 | 1 | 1 | 1 | 1 | 1 | 1 | 0 |
| Jarrar 2007 | 6 | 1 | 0 | 1 | 1 | 1 | 0 | 1 | 1 | 0 |
| Jiang 2018 | 5 | 1 | 0 | 0 | 1 | 1 | 0 | 1 | 1 | 0 |
| Kobyliak 2017 | 5 | 1 | 0 | 0 | 1 | 1 | 0 | 1 | 1 | 0 |
| Kugelmas 2003 | 5 | 1 | 0 | 0 | 1 | 1 | 0 | 1 | 1 | 0 |
| Kukla 2010 | 6 | 1 | 0 | 1 | 1 | 1 | 0 | 1 | 1 | 0 |
| Kumar 2013 | 5 | 1 | 1 | 0 | 1 | 0 | 0 | 1 | 1 | 0 |
| Louthan 2005 | 5 | 1 | 0 | 0 | 1 | 1 | 0 | 1 | 1 | 0 |
| Musso 2017 | 8 | 1 | 1 | 1 | 1 | 1 | 1 | 1 | 1 | 0 |
| Page 2013 | 7 | 1 | 1 | 1 | 1 | 1 | 0 | 1 | 1 | 0 |
| Parker 2015 | 5 | 1 | 0 | 0 | 1 | 1 | 0 | 1 | 1 | 0 |
| Perito 2017 | 8 | 1 | 1 | 1 | 1 | 1 | 1 | 1 | 1 | 0 |
| Plessis 2016 | 8 | 1 | 1 | 1 | 1 | 1 | 1 | 1 | 1 | 0 |
| Polat 2013 | 5 | 1 | 0 | 0 | 1 | 1 | 0 | 1 | 1 | 0 |
| Polyzos 2014 | 8 | 1 | 1 | 1 | 1 | 1 | 1 | 1 | 1 | 0 |
| Ponziani 2019 | 5 | 1 | 0 | 0 | 1 | 1 | 0 | 1 | 1 | 0 |
| Qi 2017 | 6 | 1 | 1 | 0 | 1 | 1 | 0 | 1 | 1 | 0 |
| Serhal 2016 | 5 | 1 | 0 | 0 | 1 | 1 | 0 | 1 | 1 | 0 |
| Shanab 2011 | 5 | 1 | 0 | 0 | 1 | 1 | 0 | 1 | 1 | 0 |
| Shoji 2016 | 4 | 1 | 0 | 0 | 1 | 1 | 0 | 0 | 1 | 0 |
| Tang 2014 | 4 | 0 | 0 | 0 | 1 | 1 | 0 | 1 | 1 | 0 |
| Tarantino 2014 | 6 | 1 | 0 | 1 | 1 | 1 | 0 | 1 | 1 | 0 |
| Torer 2007 | 6 | 1 | 0 | 1 | 1 | 1 | 0 | 1 | 1 | 0 |
| Tuncer 2003 | 4 | 1 | 0 | 0 | 0 | 1 | 0 | 1 | 1 | 0 |
| Uysal 2011 | 5 | 1 | 0 | 0 | 1 | 1 | 0 | 1 | 1 | 0 |
| Wada 2017 | 7 | 1 | 1 | 1 | 1 | 1 | 0 | 1 | 1 | 0 |
| Yang 2017 | 7 | 1 | 1 | 1 | 1 | 1 | 0 | 1 | 1 | 0 |
| Ye 2014 | 8 | 1 | 1 | 1 | 1 | 1 | 1 | 1 | 1 | 0 |
| Yilmaz 2010 | 7 | 1 | 1 | 1 | 1 | 1 | 0 | 1 | 1 | 0 |
| Yilmaz 2011 | 7 | 1 | 1 | 1 | 1 | 1 | 0 | 1 | 1 | 0 |
| Youness 2018 | 6 | 1 | 0 | 1 | 1 | 1 | 0 | 1 | 1 | 0 |
| Younossi 2008 | 6 | 1 | 0 | 1 | 1 | 1 | 0 | 1 | 1 | 0 |
| Zhang 2014 | 5 | 1 | 0 | 0 | 1 | 1 | 0 | 1 | 1 | 0 |
| Zhu 2016 | 8 | 1 | 1 | 1 | 1 | 1 | 1 | 1 | 1 | 0 |

**Appendix 7b: GRADE for the primary outcomes of NAFL** group

|  |  |  | **Study limitations** | **Imprecision** | **Inconsistency** | **Indirectness** | **Publication bias** | **GRADE** |
| --- | --- | --- | --- | --- | --- | --- | --- | --- |
| CCL2 | vs | Control | No downgrade | No downgrade | No downgrade | No downgrade | No downgrade | **MODERATE** |
| CCL3 | vs | Control | No downgrade | No downgrade | No downgrade | No downgrade | No downgrade | **MODERATE** |
| CCL4 | vs | Control | No downgrade | No downgrade | No downgrade | No downgrade | Downgrade | **LOW** |
| CCL5 | vs | Control | No downgrade | No downgrade | No downgrade | No downgrade | No downgrade | **MODERATE** |
| CCL11 | vs | Control | No downgrade | No downgrade | No downgrade | No downgrade | No downgrade | **MODERATE** |
| CCL13 | vs | Control | No downgrade | No downgrade | No downgrade | No downgrade | Downgrade | **LOW** |
| CCL19 | vs | Control | No downgrade | No downgrade | No downgrade | No downgrade | No downgrade | **MODERATE** |
| CCL21 | vs | Control | No downgrade | Downgrade because SMD between -0.80 to 0.80 | No downgrade | No downgrade | No downgrade | **LOW** |
| CCL22 | vs | Control | No downgrade | No downgrade | No downgrade | No downgrade | No downgrade | **MODERATE** |
| CXCL8 | vs | Control | No downgrade | No downgrade | No downgrade | No downgrade | No downgrade | **MODERATE** |
| CXCL10 | vs | Control | No downgrade | No downgrade | No downgrade | No downgrade | Downgrade | **LOW** |
| CXCL16 | vs | Control | No downgrade | Downgrade because SMD between -0.80 to 0.80 | No downgrade | No downgrade | No downgrade | **LOW** |
| Chemerin | vs | Control | No downgrade | Downgrade because SMD between -0.80 to 0.80 | No downgrade | No downgrade | No downgrade | **LOW** |
| CCL3 | vs | CCL2 | No downgrade | Downgrade because SMD between -0.80 to 0.80 | No downgrade | No downgrade | No downgrade | **LOW** |
| CCL4 | vs | CCL2 | No downgrade | Downgrade because SMD between -0.80 to 0.80 | No downgrade | No downgrade | No downgrade | **LOW** |
| CCL5 | vs | CCL2 | No downgrade | Downgrade because SMD between -0.80 to 0.80 | No downgrade | No downgrade | No downgrade | **LOW** |
| CCL11 | vs | CCL2 | No downgrade | Downgrade because SMD between -0.80 to 0.80 | No downgrade | No downgrade | No downgrade | **LOW** |
| CCL13 | vs | CCL2 | No downgrade | Downgrade because SMD between -0.80 to 0.80 | No downgrade | No downgrade | No downgrade | **LOW** |
| CCL19 | vs | CCL2 | No downgrade | Downgrade because SMD between -0.80 to 0.80 | No downgrade | No downgrade | No downgrade | **LOW** |
| CCL21 | vs | CCL2 | No downgrade | No downgrade | No downgrade | No downgrade | No downgrade | **MODERATE** |
| CCL22 | vs | CCL2 | No downgrade | Downgrade because SMD between -0.80 to 0.80 | No downgrade | No downgrade | No downgrade | **LOW** |
| CXCL8 | vs | CCL2 | No downgrade | Downgrade because SMD between -0.80 to 0.80 | No downgrade | No downgrade | No downgrade | **LOW** |
| CXCL10 | vs | CCL2 | No downgrade | Downgrade because SMD between -0.80 to 0.80 | No downgrade | No downgrade | No downgrade | **LOW** |
| CXCL16 | vs | CCL2 | No downgrade | No downgrade | No downgrade | No downgrade | No downgrade | **MODERATE** |
| Chemerin | vs | CCL2 | No downgrade | No downgrade | No downgrade | No downgrade | No downgrade | **MODERATE** |
| CCL4 | vs | CCL3 | No downgrade | Downgrade because SMD between -0.80 to 0.80 | No downgrade | No downgrade | No downgrade | **LOW** |
| CCL5 | vs | CCL3 | No downgrade | Downgrade because SMD between -0.80 to 0.80 | No downgrade | No downgrade | No downgrade | **LOW** |
| CCL11 | vs | CCL3 | No downgrade | Downgrade because SMD between -0.80 to 0.80 | No downgrade | No downgrade | No downgrade | **LOW** |
| CCL13 | vs | CCL3 | No downgrade | Downgrade because SMD between -0.80 to 0.80 | No downgrade | No downgrade | No downgrade | **LOW** |
| CCL19 | vs | CCL3 | No downgrade | Downgrade because SMD between -0.80 to 0.80 | No downgrade | No downgrade | No downgrade | **LOW** |
| CCL21 | vs | CCL3 | No downgrade | No downgrade | No downgrade | No downgrade | No downgrade | **MODERATE** |
| CCL22 | vs | CCL3 | No downgrade | Downgrade because SMD between -0.80 to 0.80 | No downgrade | No downgrade | No downgrade | **LOW** |
| CXCL8 | vs | CCL3 | No downgrade | Downgrade because SMD between -0.80 to 0.80 | No downgrade | No downgrade | No downgrade | **LOW** |
| CXCL10 | vs | CCL3 | No downgrade | Downgrade because SMD between -0.80 to 0.80 | No downgrade | No downgrade | No downgrade | **LOW** |
| CXCL16 | vs | CCL3 | No downgrade | No downgrade | No downgrade | No downgrade | No downgrade | **MODERATE** |
| Chemerin | vs | CCL3 | No downgrade | Downgrade because SMD between -0.80 to 0.80 | No downgrade | No downgrade | No downgrade | **LOW** |
| CCL5 | vs | CCL4 | No downgrade | Downgrade because SMD between -0.80 to 0.80 | No downgrade | No downgrade | No downgrade | **LOW** |
| CCL11 | vs | CCL4 | No downgrade | Downgrade because SMD between -0.80 to 0.80 | No downgrade | No downgrade | No downgrade | **LOW** |
| CCL13 | vs | CCL4 | No downgrade | Downgrade because SMD between -0.80 to 0.80 | No downgrade | No downgrade | No downgrade | **LOW** |
| CCL19 | vs | CCL4 | No downgrade | Downgrade because SMD between -0.80 to 0.80 | No downgrade | No downgrade | No downgrade | **LOW** |
| CCL21 | vs | CCL4 | No downgrade | Downgrade because SMD between -0.80 to 0.80 | No downgrade | No downgrade | No downgrade | **LOW** |
| CCL22 | vs | CCL4 | No downgrade | Downgrade because SMD between -0.80 to 0.80 | No downgrade | No downgrade | No downgrade | **LOW** |
| CXCL8 | vs | CCL4 | No downgrade | Downgrade because SMD between -0.80 to 0.80 | No downgrade | No downgrade | No downgrade | **LOW** |
| CXCL10 | vs | CCL4 | No downgrade | Downgrade because SMD between -0.80 to 0.80 | No downgrade | No downgrade | No downgrade | **LOW** |
| CXCL16 | vs | CCL4 | No downgrade | No downgrade | No downgrade | No downgrade | No downgrade | **MODERATE** |
| Chemerin | vs | CCL4 | No downgrade | Downgrade because SMD between -0.80 to 0.80 | No downgrade | No downgrade | No downgrade | **LOW** |
| CCL11 | vs | CCL5 | No downgrade | No downgrade | No downgrade | No downgrade | No downgrade | **MODERATE** |
| CCL13 | vs | CCL5 | No downgrade | Downgrade because SMD between -0.80 to 0.80 | No downgrade | No downgrade | No downgrade | **LOW** |
| CCL19 | vs | CCL5 | No downgrade | Downgrade because SMD between -0.80 to 0.80 | No downgrade | No downgrade | No downgrade | **LOW** |
| CCL21 | vs | CCL5 | No downgrade | No downgrade | No downgrade | No downgrade | No downgrade | **MODERATE** |
| CCL22 | vs | CCL5 | No downgrade | Downgrade because SMD between -0.80 to 0.80 | No downgrade | No downgrade | No downgrade | **LOW** |
| CXCL8 | vs | CCL5 | No downgrade | Downgrade because SMD between -0.80 to 0.80 | No downgrade | No downgrade | No downgrade | **LOW** |
| CXCL10 | vs | CCL5 | No downgrade | Downgrade because SMD between -0.80 to 0.80 | No downgrade | No downgrade | No downgrade | **LOW** |
| CXCL16 | vs | CCL5 | No downgrade | No downgrade | No downgrade | No downgrade | No downgrade | **MODERATE** |
| Chemerin | vs | CCL5 | No downgrade | No downgrade | No downgrade | No downgrade | No downgrade | **MODERATE** |
| CCL13 | vs | CCL11 | No downgrade | Downgrade because SMD between -0.80 to 0.80 | No downgrade | No downgrade | No downgrade | **LOW** |
| CCL19 | vs | CCL11 | No downgrade | No downgrade | No downgrade | No downgrade | No downgrade | **MODERATE** |
| CCL21 | vs | CCL11 | No downgrade | Downgrade because SMD between -0.80 to 0.80 | No downgrade | No downgrade | No downgrade | **LOW** |
| CCL22 | vs | CCL11 | No downgrade | Downgrade because SMD between -0.80 to 0.80 | No downgrade | No downgrade | No downgrade | **LOW** |
| CXCL8 | vs | CCL11 | No downgrade | No downgrade | No downgrade | No downgrade | No downgrade | **MODERATE** |
| CXCL10 | vs | CCL11 | No downgrade | Downgrade because SMD between -0.80 to 0.80 | No downgrade | No downgrade | No downgrade | **LOW** |
| CXCL16 | vs | CCL11 | No downgrade | Downgrade because SMD between -0.80 to 0.80 | No downgrade | No downgrade | No downgrade | **LOW** |
| Chemerin | vs | CCL11 | No downgrade | Downgrade because SMD between -0.80 to 0.80 | No downgrade | No downgrade | No downgrade | **LOW** |
| CCL19 | vs | CCL13 | No downgrade | Downgrade because SMD between -0.80 to 0.80 | No downgrade | No downgrade | No downgrade | **LOW** |
| CCL21 | vs | CCL13 | No downgrade | No downgrade | No downgrade | No downgrade | No downgrade | **MODERATE** |
| CCL22 | vs | CCL13 | No downgrade | Downgrade because SMD between -0.80 to 0.80 | No downgrade | No downgrade | No downgrade | **LOW** |
| CXCL8 | vs | CCL13 | No downgrade | Downgrade because SMD between -0.80 to 0.80 | No downgrade | No downgrade | No downgrade | **LOW** |
| CXCL10 | vs | CCL13 | No downgrade | Downgrade because SMD between -0.80 to 0.80 | No downgrade | No downgrade | No downgrade | **LOW** |
| CXCL16 | vs | CCL13 | No downgrade | No downgrade | No downgrade | No downgrade | No downgrade | **MODERATE** |
| Chemerin | vs | CCL13 | No downgrade | No downgrade | No downgrade | No downgrade | No downgrade | **MODERATE** |
| CCL21 | vs | CCL19 | No downgrade | No downgrade | No downgrade | No downgrade | No downgrade | **MODERATE** |
| CCL22 | vs | CCL19 | No downgrade | Downgrade because SMD between -0.80 to 0.80 | No downgrade | No downgrade | No downgrade | **LOW** |
| CXCL8 | vs | CCL19 | No downgrade | Downgrade because SMD between -0.80 to 0.80 | No downgrade | No downgrade | No downgrade | **LOW** |
| CXCL10 | vs | CCL19 | No downgrade | Downgrade because SMD between -0.80 to 0.80 | No downgrade | No downgrade | No downgrade | **LOW** |
| CXCL16 | vs | CCL19 | No downgrade | No downgrade | No downgrade | No downgrade | No downgrade | **MODERATE** |
| Chemerin | vs | CCL19 | No downgrade | No downgrade | No downgrade | No downgrade | No downgrade | **MODERATE** |
| CCL22 | vs | CCL21 | No downgrade | No downgrade | No downgrade | No downgrade | No downgrade | **MODERATE** |
| CXCL8 | vs | CCL21 | No downgrade | No downgrade | No downgrade | No downgrade | No downgrade | **MODERATE** |
| CXCL10 | vs | CCL21 | No downgrade | No downgrade | No downgrade | No downgrade | No downgrade | **MODERATE** |
| CXCL16 | vs | CCL21 | No downgrade | Downgrade because SMD between -0.80 to 0.80 | No downgrade | No downgrade | No downgrade | **LOW** |
| Chemerin | vs | CCL21 | No downgrade | Downgrade because SMD between -0.80 to 0.80 | No downgrade | No downgrade | No downgrade | **LOW** |
| CXCL8 | vs | CCL22 | No downgrade | Downgrade because SMD between -0.80 to 0.80 | No downgrade | No downgrade | No downgrade | **LOW** |
| CXCL10 | vs | CCL22 | No downgrade | Downgrade because SMD between -0.80 to 0.80 | No downgrade | No downgrade | No downgrade | **LOW** |
| CXCL16 | vs | CCL22 | No downgrade | No downgrade | No downgrade | No downgrade | No downgrade | **MODERATE** |
| Chemerin | vs | CCL22 | No downgrade | No downgrade | No downgrade | No downgrade | No downgrade | **MODERATE** |
| CXCL10 | vs | CXCL8 | No downgrade | Downgrade because SMD between -0.80 to 0.80 | No downgrade | No downgrade | No downgrade | **LOW** |
| CXCL16 | vs | CXCL8 | No downgrade | No downgrade | No downgrade | No downgrade | No downgrade | **MODERATE** |
| Chemerin | vs | CXCL8 | No downgrade | No downgrade | No downgrade | No downgrade | No downgrade | **MODERATE** |
| CXCL16 | vs | CXCL10 | No downgrade | No downgrade | No downgrade | No downgrade | No downgrade | **MODERATE** |
| Chemerin | vs | CXCL10 | No downgrade | Downgrade because SMD between -0.80 to 0.80 | No downgrade | No downgrade | No downgrade | **LOW** |
| Chemerin | vs | CXCL16 | No downgrade | Downgrade because SMD between -0.80 to 0.80 | No downgrade | No downgrade | No downgrade | **LOW** |

**Appendix 7c: GRADE for the primary outcomes of NASH group**

|  |  |  | **Study limitations** | **Imprecision** | **Inconsistency** | **Indirectness** | **Publication bias** | **GRADE** |
| --- | --- | --- | --- | --- | --- | --- | --- | --- |
| CCL2 | vs | Control | No downgrade | Downgrade because SMD between -0.80 to 0.80 | Downgrade because p=0.046 | No downgrade | No downgrade | **LOW** |
| CCL3 | vs | Control | No downgrade | No downgrade | No downgrade | No downgrade | No downgrade | **MODERATE** |
| CCL4 | vs | Control | No downgrade | No downgrade | No downgrade | No downgrade | No downgrade | **MODERATE** |
| CCL13 | vs | Control | No downgrade | No downgrade | No downgrade | No downgrade | Downgrade | **MODERATE** |
| CCL19 | vs | Control | No downgrade | No downgrade | No downgrade | No downgrade | No downgrade | **MODERATE** |
| CCL20 | vs | Control | No downgrade | No downgrade | No downgrade | No downgrade | No downgrade | **MODERATE** |
| CCL21 | vs | Control | No downgrade | Downgrade because SMD between -0.80 to 0.80 | No downgrade | No downgrade | No downgrade | **LOW** |
| CCL22 | vs | Control | No downgrade | Downgrade because SMD between -0.80 to 0.80 | No downgrade | No downgrade | No downgrade | **LOW** |
| CXCL8 | vs | Control | No downgrade | No downgrade | No downgrade | No downgrade | No downgrade | **MODERATE** |
| CXCL10 | vs | Control | No downgrade | No downgrade | No downgrade | No downgrade | No downgrade | **MODERATE** |
| CXCL12 | vs | Control | No downgrade | No downgrade | No downgrade | No downgrade | No downgrade | **MODERATE** |
| Chemerin | vs | Control | No downgrade | No downgrade | No downgrade | No downgrade | Downgrade | **LOW** |
| CCL3 | vs | CCL2 | No downgrade | Downgrade because SMD between -0.80 to 0.80 | No downgrade | No downgrade | No downgrade | **LOW** |
| CCL4 | vs | CCL2 | No downgrade | No downgrade | No downgrade | No downgrade | No downgrade | **MODERATE** |
| CCL13 | vs | CCL2 | No downgrade | No downgrade | No downgrade | No downgrade | No downgrade | **MODERATE** |
| CCL19 | vs | CCL2 | No downgrade | No downgrade | No downgrade | No downgrade | No downgrade | **MODERATE** |
| CCL20 | vs | CCL2 | No downgrade | No downgrade | No downgrade | No downgrade | No downgrade | **MODERATE** |
| CCL21 | vs | CCL2 | No downgrade | Downgrade because SMD between -0.80 to 0.80 | No downgrade | No downgrade | No downgrade | **LOW** |
| CCL22 | vs | CCL2 | No downgrade | Downgrade because SMD between -0.80 to 0.80 | No downgrade | No downgrade | No downgrade | **LOW** |
| CXCL8 | vs | CCL2 | No downgrade | Downgrade because SMD between -0.80 to 0.80 | No downgrade | No downgrade | Downgrade | **VERY LOW** |
| CXCL10 | vs | CCL2 | No downgrade | No downgrade | No downgrade | No downgrade | No downgrade | **MODERATE** |
| CXCL12 | vs | CCL2 | No downgrade | Downgrade because SMD between -0.80 to 0.80 | No downgrade | No downgrade | No downgrade | **LOW** |
| Chemerin | vs | CCL2 | No downgrade | No downgrade | No downgrade | No downgrade | No downgrade | **MODERATE** |
| CCL4 | vs | CCL3 | No downgrade | No downgrade | No downgrade | No downgrade | No downgrade | **MODERATE** |
| CCL13 | vs | CCL3 | No downgrade | Downgrade because SMD between -0.80 to 0.80 | No downgrade | No downgrade | No downgrade | **LOW** |
| CCL19 | vs | CCL3 | No downgrade | Downgrade because SMD between -0.80 to 0.80 | No downgrade | No downgrade | No downgrade | **LOW** |
| CCL20 | vs | CCL3 | No downgrade | No downgrade | No downgrade | No downgrade | No downgrade | **MODERATE** |
| CCL21 | vs | CCL3 | No downgrade | No downgrade | No downgrade | No downgrade | No downgrade | **MODERATE** |
| CCL22 | vs | CCL3 | No downgrade | Downgrade because SMD between -0.80 to 0.80 | No downgrade | No downgrade | No downgrade | **LOW** |
| CXCL8 | vs | CCL3 | No downgrade | Downgrade because SMD between -0.80 to 0.80 | No downgrade | No downgrade | No downgrade | **LOW** |
| CXCL10 | vs | CCL3 | No downgrade | Downgrade because SMD between -0.80 to 0.80 | Downgrade because p=0.006 | No downgrade | No downgrade | **VERY LOW** |
| CXCL12 | vs | CCL3 | No downgrade | Downgrade because SMD between -0.80 to 0.80 | No downgrade | No downgrade | No downgrade | **LOW** |
| Chemerin | vs | CCL3 | No downgrade | No downgrade | No downgrade | No downgrade | No downgrade | **MODERATE** |
| CCL13 | vs | CCL4 | No downgrade | Downgrade because SMD between -0.80 to 0.80 | No downgrade | No downgrade | No downgrade | **LOW** |
| CCL19 | vs | CCL4 | No downgrade | Downgrade because SMD between -0.80 to 0.80 | No downgrade | No downgrade | No downgrade | **LOW** |
| CCL20 | vs | CCL4 | No downgrade | Downgrade because SMD between -0.80 to 0.80 | No downgrade | No downgrade | No downgrade | **LOW** |
| CCL21 | vs | CCL4 | No downgrade | No downgrade | No downgrade | No downgrade | No downgrade | **MODERATE** |
| CCL22 | vs | CCL4 | No downgrade | No downgrade | No downgrade | No downgrade | No downgrade | **MODERATE** |
| CXCL8 | vs | CCL4 | No downgrade | No downgrade | No downgrade | No downgrade | No downgrade | **MODERATE** |
| CXCL10 | vs | CCL4 | No downgrade | Downgrade because SMD between -0.80 to 0.80 | Downgrade because p=0.017 | No downgrade | No downgrade | **VERY LOW** |
| CXCL12 | vs | CCL4 | No downgrade | No downgrade | No downgrade | No downgrade | No downgrade | **MODERATE** |
| Chemerin | vs | CCL4 | No downgrade | No downgrade | No downgrade | No downgrade | No downgrade | **MODERATE** |
| CCL19 | vs | CCL13 | No downgrade | Downgrade because SMD between -0.80 to 0.80 | No downgrade | No downgrade | No downgrade | **LOW** |
| CCL20 | vs | CCL13 | No downgrade | No downgrade | No downgrade | No downgrade | No downgrade | **MODERATE** |
| CCL21 | vs | CCL13 | No downgrade | No downgrade | No downgrade | No downgrade | No downgrade | **MODERATE** |
| CCL22 | vs | CCL13 | No downgrade | Downgrade because SMD between -0.80 to 0.80 | No downgrade | No downgrade | No downgrade | **LOW** |
| CXCL8 | vs | CCL13 | No downgrade | Downgrade because SMD between -0.80 to 0.80 | No downgrade | No downgrade | No downgrade | **LOW** |
| CXCL10 | vs | CCL13 | No downgrade | Downgrade because SMD between -0.80 to 0.80 | No downgrade | No downgrade | No downgrade | **LOW** |
| CXCL12 | vs | CCL13 | No downgrade | Downgrade because SMD between -0.80 to 0.80 | No downgrade | No downgrade | No downgrade | **LOW** |
| Chemerin | vs | CCL13 | No downgrade | No downgrade | No downgrade | No downgrade | No downgrade | **MODERATE** |
| CCL20 | vs | CCL19 | No downgrade | No downgrade | No downgrade | No downgrade | No downgrade | **MODERATE** |
| CCL21 | vs | CCL19 | No downgrade | No downgrade | No downgrade | No downgrade | No downgrade | **MODERATE** |
| CCL22 | vs | CCL19 | No downgrade | Downgrade because SMD between -0.80 to 0.80 | No downgrade | No downgrade | No downgrade | **LOW** |
| CXCL8 | vs | CCL19 | No downgrade | Downgrade because SMD between -0.80 to 0.80 | No downgrade | No downgrade | No downgrade | **LOW** |
| CXCL10 | vs | CCL19 | No downgrade | Downgrade because SMD between -0.80 to 0.80 | No downgrade | No downgrade | No downgrade | **LOW** |
| CXCL12 | vs | CCL19 | No downgrade | Downgrade because SMD between -0.80 to 0.80 | No downgrade | No downgrade | No downgrade | **LOW** |
| Chemerin | vs | CCL19 | No downgrade | No downgrade | No downgrade | No downgrade | No downgrade | **MODERATE** |
| CCL21 | vs | CCL20 | No downgrade | No downgrade | No downgrade | No downgrade | No downgrade | **MODERATE** |
| CCL22 | vs | CCL20 | No downgrade | No downgrade | No downgrade | No downgrade | No downgrade | **MODERATE** |
| CXCL8 | vs | CCL20 | No downgrade | No downgrade | No downgrade | No downgrade | No downgrade | **MODERATE** |
| CXCL10 | vs | CCL20 | No downgrade | Downgrade because SMD between -0.80 to 0.80 | No downgrade | No downgrade | No downgrade | **LOW** |
| CXCL12 | vs | CCL20 | No downgrade | No downgrade | No downgrade | No downgrade | No downgrade | **MODERATE** |
| Chemerin | vs | CCL20 | No downgrade | No downgrade | No downgrade | No downgrade | No downgrade | **MODERATE** |
| CCL22 | vs | CCL21 | No downgrade | Downgrade because SMD between -0.80 to 0.80 | No downgrade | No downgrade | No downgrade | **LOW** |
| CXCL8 | vs | CCL21 | No downgrade | No downgrade | No downgrade | No downgrade | No downgrade | **MODERATE** |
| CXCL10 | vs | CCL21 | No downgrade | No downgrade | No downgrade | No downgrade | No downgrade | **MODERATE** |
| CXCL12 | vs | CCL21 | No downgrade | No downgrade | No downgrade | No downgrade | No downgrade | **MODERATE** |
| Chemerin | vs | CCL21 | No downgrade | No downgrade | No downgrade | No downgrade | No downgrade | **MODERATE** |
| CXCL8 | vs | CCL22 | No downgrade | Downgrade because SMD between -0.80 to 0.80 | No downgrade | No downgrade | No downgrade | **LOW** |
| CXCL10 | vs | CCL22 | No downgrade | No downgrade | No downgrade | No downgrade | No downgrade | **MODERATE** |
| CXCL12 | vs | CCL22 | No downgrade | Downgrade because SMD between -0.80 to 0.80 | No downgrade | No downgrade | No downgrade | **LOW** |
| Chemerin | vs | CCL22 | No downgrade | No downgrade | No downgrade | No downgrade | No downgrade | **MODERATE** |
| CXCL10 | vs | CXCL8 | No downgrade | Downgrade because SMD between -0.80 to 0.80 | No downgrade | No downgrade | No downgrade | **LOW** |
| CXCL12 | vs | CXCL8 | No downgrade | Downgrade because SMD between -0.80 to 0.80 | No downgrade | No downgrade | No downgrade | **LOW** |
| Chemerin | vs | CXCL8 | No downgrade | No downgrade | No downgrade | No downgrade | No downgrade | **MODERATE** |
| CXCL12 | vs | CXCL10 | No downgrade | Downgrade because SMD between -0.80 to 0.80 | No downgrade | No downgrade | No downgrade | **LOW** |
| Chemerin | vs | CXCL10 | No downgrade | No downgrade | No downgrade | No downgrade | No downgrade | **MODERATE** |
| Chemerin | vs | CXCL12 | No downgrade | No downgrade | No downgrade | No downgrade | No downgrade | **MODERATE** |

Table of reasons for downgrading Based on all the above information, we GRADE each network estimate according to the following criteria.

(1) Study limitations: We downgraded by one level when the contributions from low quality of NOS comparisons were more than 30%.

(2) Imprecision: We considered a clinically meaningful threshold for SMD to be -0.80 to 0.80 and will downgraded the estimate, if the SMD point estimate is 0.80 or more or below -0.80 will not downgraded the estimate;

(3) Inconsistency: We rated two concepts, heterogeneity and incoherence (inconsistency), in this domain. For heterogeneity, we looked at the common tau, If the difference was statistically significant, it was downgraded (p<0.05). For inconsistency, we looked at the results of side splitting and we downgraded the comparisons with important inconsistency (p<0.05).

(4) Indirectness: We have assured transitivity in our network by limiting the included studies. Thus, the review team decided by default will not downgrade all the included studies for indirectness.

(5) Publication bias: The comparison-adjusted funnel plot did not suggest presence of overall publication bias in comparison. Therefore, we degrade the outliers in the funnel graph.

**Appendix 8a: Loop-Specific heterogeneity for the network of Chemokines in NAFL group**


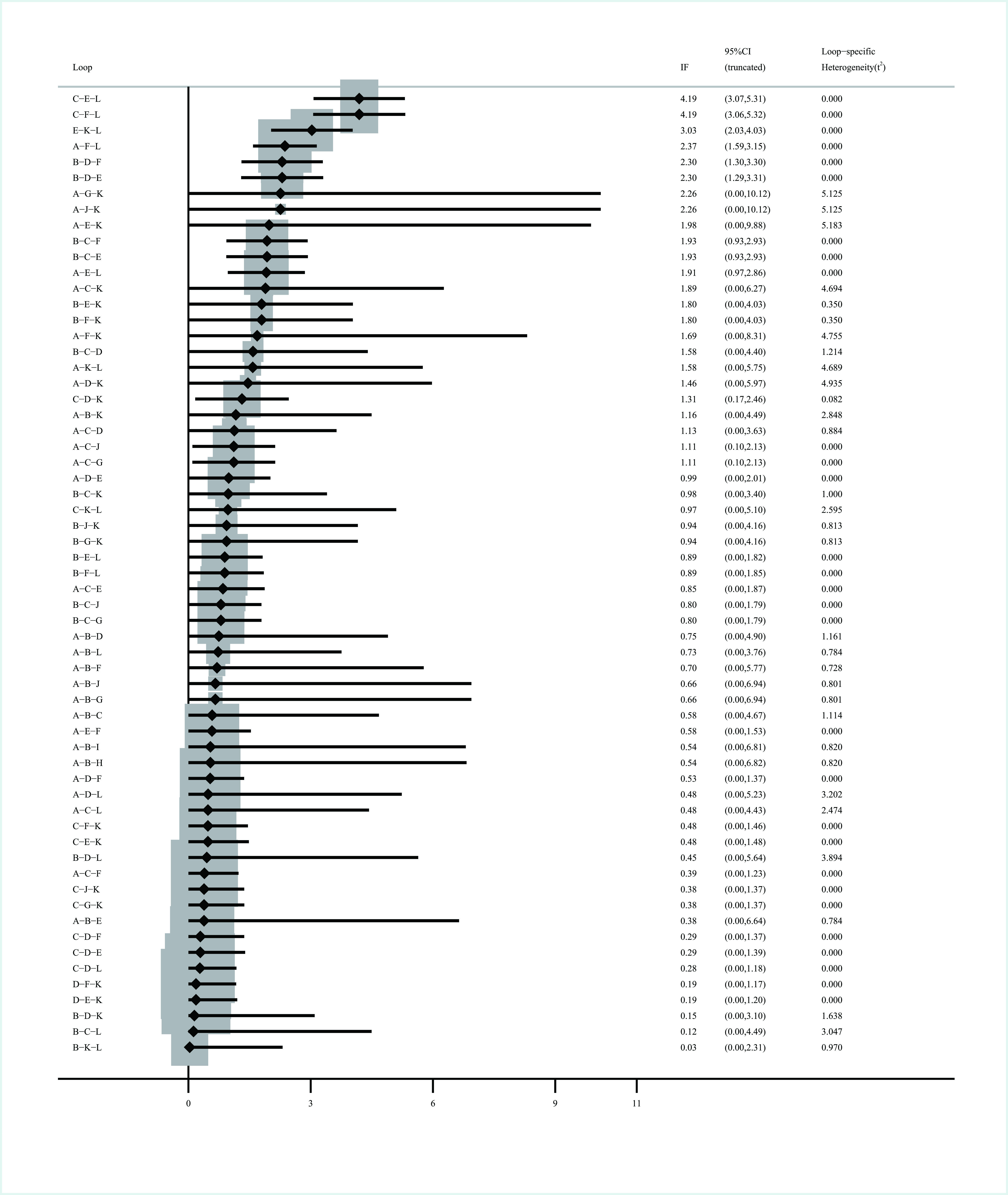


A, Control; B, CCL2; C, CCL3; D, CCL4; E, CCL13; F, CCL19; G, CCL20; H, CCL21; I, CCL22; J, CXCL8; K, CXCL10; L, CXCL12; M, Chemerin

**Appendix 8b: Loop-Specific heterogeneity for the network of Chemokines in NASH group**


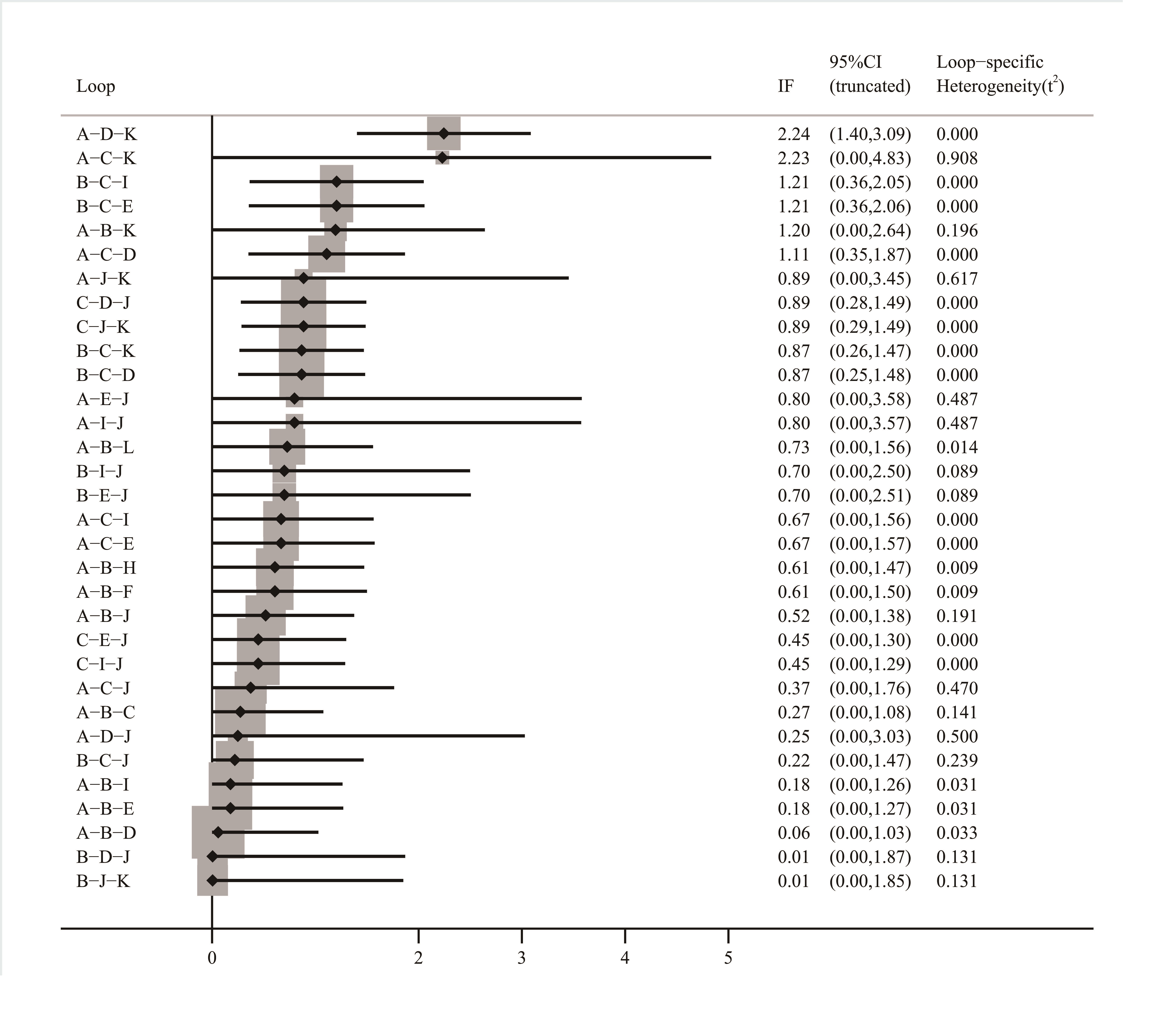


A, Control; B, CCL2; C, CCL3; D, CCL4; E, CCL13; F, CCL19; G, CCL20; H, CCL21; I, CCL22; J, CXCL8; K, CXCL10; L, CXCL12; M, Chemerin

**Appendix 9a: Sensitivity analyses for the rank order (SUCRA ranks) in differing chemokine in NAFL**.

| **NAFL** | **SUCRA** | **MeanRank** | **NAFL** | **SUCRA** | **MeanRank** | **NAFL** | **SUCRA** | **MeanRank** |
| --- | --- | --- | --- | --- | --- | --- | --- | --- |
| CXCL8 | 69.4 | 5.0 | CXCL8 | 71.4 | 4.4 | CXCL8 | 71.1 | 4.5 |
| CCL5 | 65 | 5.6 | CCL5 | 66.7 | 5.0 | CCL19 | 60.2 | 5.8 |
| CCL19 | 59.5 | 6.3 | CCL13 | 58.4 | 6.0 | CCL2 | 59.2 | 5.9 |
| CCL2 | 57.6 | 6.5 | CCL22 | 57.7 | 6.1 | CCL13 | 57.3 | 6.1 |
| CCL13 | 57 | 6.6 | CCL3 | 53.8 | 6.5 | CXCL10 | 57.6 | 6.1 |
| CCL22 | 55.3 | 6.8 | CCL19 | 53.6 | 6.6 | CCL22 | 56.7 | 6.2 |
| CXCL10 | 55.1 | 6.8 | CXCL10 | 52.9 | 6.7 | CCL3 | 55.5 | 6.3 |
| CCL3 | 54.6 | 6.9 | CCL4 | 49.1 | 7.1 | CCL4 | 51.9 | 6.8 |
| CCL4 | 51.1 | 7.4 | CCL11 | 45.8 | 7.5 | CCL11 | 43.7 | 7.8 |
| CCL11 | 42.8 | 8.4 | Chemerin | 42.5 | 7.9 | CCL21 | 40.6 | 8.1 |
| CCL21 | 39.2 | 8.9 | CXCL16 | 40.5 | 8.1 | CXCL16 | 38.4 | 8.4 |
| CXCL16 | 37.9 | 9.1 | CCL21 | 34.5 | 8.9 | Chemerin | 38.6 | 8.4 |
| Chemerin | 36.7 | 9.2 | Control | 23.2 | 10.2 | Control | 19.3 | 10.7 |
| Control | 18.6 | 11.6 |  |  |  |  |  |  |

**Table continued.**

| **NAFL** | **SUCRA** | **MeanRank** | **NAFL** | **SUCRA** | **MeanRank** | **NAFL** | **SUCRA** | **MeanRank** |
| --- | --- | --- | --- | --- | --- | --- | --- | --- |
| CXCL8 | 69.6 | 4.6 | CXCL8 | 69.4 | 4.7 | CXCL8 | 68.3 | 4.8 |
| CCL4 | 64.2 | 5.3 | CCL5 | 62.0 | 5.6 | CCL5 | 65.7 | 5.1 |
| CCL5 | 63.0 | 5.4 | CCL19 | 59.2 | 5.9 | CCL19 | 58.4 | 6.0 |
| CCL19 | 58.0 | 6.0 | CCL2 | 57.6 | 6.1 | CCL2 | 56.8 | 6.2 |
| CCL2 | 55.5 | 6.3 | CCL3 | 56.3 | 6.2 | CCL13 | 55.6 | 6.3 |
| CCL13 | 53.6 | 6.6 | CCL13 | 56.7 | 6.2 | CXCL10 | 55.6 | 6.3 |
| CCL22 | 53.4 | 6.6 | CCL22 | 56.1 | 6.3 | CCL22 | 54.8 | 6.4 |
| CXCL10 | 53.0 | 6.6 | CXCL10 | 54.6 | 6.4 | CCL3 | 53.3 | 6.6 |
| CCL11 | 43.3 | 7.8 | CCL11 | 42.0 | 8.0 | CCL4 | 49.6 | 7.0 |
| CCL21 | 39.9 | 8.2 | CCL21 | 40.1 | 8.2 | CCL21 | 39.3 | 8.3 |
| CXCL16 | 38.2 | 8.4 | CXCL16 | 38.1 | 8.4 | CXCL16 | 37.3 | 8.5 |
| Chemerin | 38.2 | 8.4 | Chemerin | 38.3 | 8.4 | Chemerin | 37.0 | 8.6 |
| Control | 20.2 | 10.6 | Control | 19.7 | 10.6 | Control | 18.2 | 10.8 |

**Table continued.**

| **NAFL** | **SUCRA** | **MeanRank** | **NAFL** | **SUCRA** | **MeanRank** | **NAFL** | **SUCRA** | **MeanRank** |
| --- | --- | --- | --- | --- | --- | --- | --- | --- |
| CXCL8 | 70.7 | 4.5 | CXCL8 | 71.0 | 4.5 | CXCL8 | 69.4 | 4.7 |
| CCL5 | 65.5 | 5.1 | CCL5 | 65.8 | 5.1 | CCL5 | 64.4 | 5.3 |
| CCL19 | 59.7 | 5.8 | CCL2 | 58.6 | 6.0 | CCL2 | 56.6 | 6.2 |
| CCL2 | 58.1 | 6.0 | CCL13 | 57.7 | 6.1 | CCL13 | 56.5 | 6.2 |
| CCL22 | 56.3 | 6.2 | CCL22 | 56.4 | 6.2 | CCL19 | 57.0 | 6.2 |
| CXCL10 | 55.6 | 6.3 | CCL3 | 55.6 | 6.3 | CCL22 | 55.1 | 6.4 |
| CCL3 | 55.3 | 6.4 | CXCL10 | 56.0 | 6.3 | CCL3 | 54.0 | 6.5 |
| CCL4 | 51.6 | 6.8 | CCL4 | 51.9 | 6.8 | CXCL10 | 54.4 | 6.5 |
| CCL11 | 42.3 | 7.9 | CCL11 | 42.5 | 7.9 | CCL4 | 50.3 | 7.0 |
| CCL21 | 40.1 | 8.2 | CCL21 | 39.7 | 8.2 | CCL11 | 41.1 | 8.1 |
| CXCL16 | 38.0 | 8.4 | CXCL16 | 38.1 | 8.4 | CXCL16 | 37.0 | 8.6 |
| Chemerin | 38.0 | 8.4 | Chemerin | 38.1 | 8.4 | Chemerin | 36.7 | 8.6 |
| Control | 18.7 | 10.8 | Control | 18.7 | 10.8 | Control | 17.3 | 10.9 |

**Table continued.**

| **NAFL** | **SUCRA** | **MeanRank** | **NAFL** | **SUCRA** | **MeanRank** | **NAFL** | **SUCRA** | **MeanRank** |
| --- | --- | --- | --- | --- | --- | --- | --- | --- |
| CXCL8 | 70.5 | 4.5 | CCL19 | 65.7 | 5.1 | CCL5 | 70 | 4.6 |
| CCL5 | 65.3 | 5.2 | CCL5 | 63.2 | 5.4 | CXCL8 | 68.1 | 4.8 |
| CCL19 | 60.1 | 5.8 | CCL2 | 61.8 | 5.6 | CCL19 | 59.2 | 5.9 |
| CCL2 | 57.9 | 6.1 | CXCL10 | 56.6 | 6.2 | CCL2 | 57.2 | 6.1 |
| CCL13 | 57.2 | 6.1 | CCL13 | 53.7 | 6.6 | CCL13 | 56.6 | 6.2 |
| CXCL10 | 55.6 | 6.3 | CCL3 | 51.3 | 6.8 | CCL22 | 55.2 | 6.4 |
| CCL3 | 55.3 | 6.4 | CCL22 | 51.9 | 6.8 | CCL3 | 53.7 | 6.6 |
| CCL4 | 51.5 | 6.8 | CCL4 | 50 | 7.0 | CCL4 | 49.9 | 7.0 |
| CCL11 | 42.2 | 7.9 | Chemerin | 45.9 | 7.5 | CCL11 | 45.3 | 7.6 |
| CCL21 | 40.1 | 8.2 | CCL21 | 42.3 | 7.9 | CCL21 | 40 | 8.2 |
| CXCL16 | 37.9 | 8.5 | CXCL16 | 42.3 | 7.9 | CXCL16 | 37.8 | 8.5 |
| Chemerin | 37.9 | 8.5 | CCL11 | 41.7 | 8.0 | Chemerin | 37.7 | 8.5 |
| Control | 18.6 | 10.8 | Control | 23.6 | 10.2 | Control | 19.4 | 10.7 |

**Table continued.**

| **NAFL** | **SUCRA** | **MeanRank** | **NAFL** | **SUCRA** | **MeanRank** |
| --- | --- | --- | --- | --- | --- |
| CXCL8 | 69.1 | 4.7 | CXCL8 | 67.3 | 4.9 |
| CCL5 | 64.2 | 5.3 | CCL5 | 63.2 | 5.4 |
| CCL19 | 59.0 | 5.9 | CCL19 | 58.5 | 6.0 |
| CCL2 | 56.5 | 6.2 | CCL2 | 55.8 | 6.3 |
| CCL13 | 56.4 | 6.2 | CCL13 | 55.9 | 6.3 |
| CCL22 | 54.9 | 6.4 | CCL22 | 54.6 | 6.4 |
| CCL3 | 53.8 | 6.5 | CCL3 | 53.2 | 6.6 |
| CXCL10 | 54.0 | 6.5 | CXCL10 | 53.4 | 6.6 |
| CCL4 | 50.0 | 7.0 | CCL4 | 49.8 | 7.0 |
| CCL11 | 40.7 | 8.1 | CCL11 | 41.1 | 8.1 |
| CCL21 | 38.8 | 8.3 | CCL21 | 39.6 | 8.3 |
| Chemerin | 36.1 | 8.7 | CXCL16 | 39.1 | 8.3 |
| Control | 16.6 | 11.0 | Control | 18.7 | 10.8 |

**Appendix 9b: Sensitivity analyses for the rank order (SUCRA ranks) in differing chemokine in** NASH.

| **NASH** | **SUCRA** | **MeanRank** | **NASH** | **SUCRA** | **MeanRank** |
| --- | --- | --- | --- | --- | --- |
| CCL20 | 91.5 | 2.0 | CCL20 | 90.8 | 2.0 |
| CCL4 | 87.6 | 2.5 | CCL4 | 86.1 | 2.5 |
| CXCL10 | 72.4 | 4.3 | CXCL10 | 71 | 4.2 |
| CCL13 | 64.9 | 5.2 | CCL13 | 61.2 | 5.3 |
| CCL19 | 64.9 | 5.2 | CCL19 | 61.3 | 5.3 |
| CXCL12 | 59.4 | 5.9 | CXCL12 | 55.4 | 5.9 |
| CCL3 | 52.7 | 6.7 | CCL3 | 48.3 | 6.7 |
| CXCL8 | 52.5 | 6.7 | CXCL8 | 48.3 | 6.7 |
| CCL22 | 39.8 | 8.2 | CCL22 | 34.7 | 8.2 |
| CCL2 | 27.7 | 9.7 | CCL2 | 21.2 | 9.7 |
| CCL21 | 19.9 | 10.6 | CCL21 | 12.6 | 10.6 |
| Control | 16.6 | 11.0 | Control | 9.1 | 11.0 |
| Chemerin | 0.0 | 13.0 |  |  |  |

Table continued.

| **NASH** | **SUCRA** | **MeanRank** | **NASH** | **SUCRA** | **MeanRank** |
| --- | --- | --- | --- | --- | --- |
| CCL20 | 88.0 | 2.3 | CCL4 | 94.4 | 1.6 |
| CCL4 | 80.2 | 3.2 | CCL20 | 88.3 | 2.3 |
| CXCL10 | 67.7 | 4.6 | CXCL10 | 75.5 | 3.7 |
| CCL13 | 64.2 | 4.9 | CCL19 | 63.1 | 5.1 |
| CCL19 | 61.1 | 5.3 | CXCL12 | 59.2 | 5.5 |
| CXCL12 | 59.0 | 5.5 | CCL13 | 58.1 | 5.6 |
| CXCL8 | 47.7 | 6.7 | CXCL8 | 53.8 | 6.1 |
| CCL3 | 46.1 | 6.9 | CCL22 | 36.5 | 8.0 |
| CCL22 | 42.0 | 7.4 | CCL2 | 30.7 | 8.6 |
| CCL21 | 26.1 | 9.1 | CCL21 | 21.0 | 9.7 |
| Control | 17.8 | 10.0 | Control | 19.4 | 9.9 |
| Chemerin | 0.0 | 12.0 | Chemerin | 0.0 | 12.0 |

Table continued.

| **NASH** | **SUCRA** | **MeanRank** | **NASH** | **SUCRA** | **MeanRank** | **NASH** | **SUCRA** | **MeanRank** |
| --- | --- | --- | --- | --- | --- | --- | --- | --- |
| CCL20 | 94.4 | 1.6 | CCL20 | 91.7 | 1.9 | CCL20 | 94.9 | 1.6 |
| CXCL10 | 77.2 | 3.5 | CCL4 | 88.0 | 2.3 | CCL4 | 83.3 | 2.8 |
| CCL13 | 68.7 | 4.4 | CXCL10 | 73.7 | 3.9 | CCL19 | 68.9 | 4.4 |
| CCL19 | 67.4 | 4.6 | CCL13 | 66.1 | 4.7 | CCL13 | 68.0 | 4.5 |
| CXCL12 | 63.5 | 5.0 | CXCL12 | 62.3 | 5.2 | CXCL12 | 64.6 | 4.9 |
| CCL3 | 59.3 | 5.5 | CCL3 | 54.7 | 6.0 | CXCL8 | 56.6 | 5.8 |
| CXCL8 | 56.4 | 5.8 | CXCL8 | 54.6 | 6.0 | CCL3 | 53.3 | 6.1 |
| CCL22 | 43.5 | 7.2 | CCL22 | 42.2 | 7.4 | CCL22 | 42.0 | 7.4 |
| CCL2 | 30 | 8.7 | CCL2 | 31.5 | 8.5 | CCL2 | 29.3 | 8.8 |
| CCL21 | 21.2 | 9.7 | Control | 18.8 | 9.9 | CCL21 | 20.6 | 9.7 |
| Control | 18.4 | 10.0 | CCL21 | 16.4 | 10.2 | Control | 18.5 | 10.0 |
| Chemerin | 0.0 | 12.0 | Chemerin | 0.0 | 12.0 | Chemerin | 0.0 | 12 |

Table continued.

| **NASH** | **SUCRA** | **MeanRank** | **NASH** | **SUCRA** | **MeanRank** | **NASH** | **SUCRA** | **MeanRank** |
| --- | --- | --- | --- | --- | --- | --- | --- | --- |
| CCL20 | 92.0 | 1.9 | CCL4 | 91.9 | 1.9 | CCL20 | 91.2 | 2.0 |
| CCL4 | 88.0 | 2.3 | CXCL10 | 78.1 | 3.4 | CCL4 | 86.6 | 2.5 |
| CXCL10 | 73.7 | 3.9 | CCL13 | 69.5 | 4.4 | CXCL10 | 70.9 | 4.2 |
| CCL19 | 65.6 | 4.8 | CCL19 | 69.3 | 4.4 | CCL13 | 62.4 | 5.1 |
| CXCL12 | 61.7 | 5.2 | CXCL12 | 65.0 | 4.8 | CXCL12 | 57.8 | 5.6 |
| CCL3 | 54.3 | 6.0 | CCL3 | 57.2 | 5.7 | CCL19 | 57.6 | 5.7 |
| CXCL8 | 54.5 | 6.0 | CXCL8 | 57.1 | 5.7 | CCL3 | 49.7 | 6.5 |
| CCL22 | 41.9 | 7.4 | CCL22 | 43.1 | 7.3 | CXCL8 | 49.6 | 6.5 |
| CCL2 | 29.6 | 8.7 | CCL2 | 30.1 | 8.7 | CCL22 | 37.1 | 7.9 |
| CCL21 | 20.7 | 9.7 | CCL21 | 20.7 | 9.7 | CCL2 | 23.9 | 9.4 |
| Control | 18.0 | 10.0 | Control | 18.0 | 10.0 | Control | 13.3 | 10.5 |
| Chemerin | 0.0 | 12 | Chemerin | 0.0 | 12 | Chemerin | 0.0 | 12 |

Table continued.

| **NASH** | **SUCRA** | **MeanRank** | **NASH** | **SUCRA** | **MeanRank** | **NASH** | **SUCRA** | **MeanRank** |
| --- | --- | --- | --- | --- | --- | --- | --- | --- |
| CCL20 | 91.2 | 2.0 | CCL20 | 93.6 | 1.7 | CCL20 | 90.9 | 2.0 |
| CCL4 | 86.8 | 2.4 | CCL4 | 84.8 | 2.7 | CCL4 | 87.1 | 2.4 |
| CXCL10 | 71.5 | 4.1 | CXCL10 | 72.7 | 4.0 | CXCL10 | 73.4 | 3.9 |
| CCL19 | 63.5 | 5.0 | CCL19 | 66.6 | 4.7 | CCL19 | 65.2 | 4.8 |
| CCL13 | 61.8 | 5.2 | CXCL12 | 62.0 | 5.2 | CCL13 | 65.0 | 4.9 |
| CXCL12 | 59.1 | 5.5 | CCL13 | 60.3 | 5.4 | CCL3 | 53.9 | 6.1 |
| CCL3 | 51.4 | 6.3 | CCL3 | 52.8 | 6.2 | CXCL8 | 53.7 | 6.1 |
| CXCL8 | 51.4 | 6.3 | CCL22 | 36.9 | 7.9 | CCL22 | 41.4 | 7.4 |
| CCL2 | 27.2 | 9.0 | CCL2 | 30.0 | 8.7 | CCL2 | 30.7 | 8.6 |
| CCL21 | 19.5 | 9.9 | CCL21 | 20.7 | 9.7 | CCL21 | 21.3 | 9.7 |
| Control | 16.5 | 10.2 | Control | 19.7 | 9.8 | Control | 17.5 | 10.1 |
| Chemerin | 0.0 | 12.0 | Chemerin | 0.0 | 12 | Chemerin | 0.0 | 12.0 |

**Appendix References**

1. Ajmera V, Perito ER, Bass NM, Terrault NA, Yates KP, Gill R, Loomba R, Diehl AM, Aouizerat BE: Novel plasma biomarkers associated with liver disease severity in adults with nonalcoholic fatty liver disease. *Hepatology* 2017, 65(1):65-77.

2. Alisi A, Nobili V, Ceccarelli S, Panera N, De Stefanis C, De Vito R, Vitali R, Bedogni G, Balsano C, Cucchiara S *et al*: Plasma high mobility group box 1 protein reflects fibrosis in pediatric nonalcoholic fatty liver disease. *Expert Review of Molecular Diagnostics* 2014, 14(6):763-771.

3. Ando W, Yokomori H, Tsutsui N, Yamanouchi E, Suzuki Y, Oda M, Inagaki Y, Otori K, Okazaki I: Serum matrix metalloproteinase-1 level represents disease activity as opposed to fibrosis in patients with histologically proven nonalcoholic steatohepatitis. *Clinical and Molecular Hepatology* 2018, 24(1):61-76.

4. Baltieri L, Chaim EA, Chaim FDM, Utrini MP, Gestic MA, Cazzo E: CORRELATION BETWEEN NONALCOHOLIC FATTY LIVER DISEASE FEATURES AND LEVELS OF ADIPOKINES AND INFLAMMATORY CYTOKINES AMONG MORBIDLY OBESE INDIVIDUALS. *Arq Gastroenterol* 2018, 55(3):247-251.

5. Barchetta I, Cimini FA, De Gioannis R, Ciccarelli G, Bertoccini L, Lenzi A, Baroni MG, Cavallo MG: Procollagen-III peptide identifies adipose tissue-associated inflammation in type 2 diabetes with or without nonalcoholic liver disease. *Diabetes/metabolism research and reviews* 2018, 34(5) (no pagination).

6. Chang C-C, Wu C-L, Su W-W, Shih K-L, Tarng D-C, Chou C-T, Chen T-Y, Kor C-T, Wu H-M: Interferon gamma-induced protein 10 is associated with insulin resistance and incident diabetes in patients with nonalcoholic fatty liver disease. *Scientific reports* 2015, 5.

7. Chu X, Jin Q, Chen H, Wood GC, Petrick A, Strodel W, Gabrielsen J, Benotti P, Mirshahi T, Carey DJ *et al*: CCL20 is up-regulated in non-alcoholic fatty liver disease fibrosis and is produced by hepatic stellate cells in response to fatty acid loading. *Journal of Translational Medicine* 2018, 16.

8. Dinu M, Whittaker A, Pagliai G, Giangrandi I, Colombini B, Gori AM, Fiorillo C, Becatti M, Casini A, Benedettelli S *et al*: A Khorasan Wheat-Based Replacement Diet Improves Risk Profile of Patients With Nonalcoholic Fatty Liver Disease (NAFLD): a Randomized Clinical Trial. *Journal of the american college of nutrition* 2018, 37(6):508‐514.

9. du Plessis J, van Pelt J, Korf H, Mathieu C, van der Schueren B, Lannoo M, Oyen T, Topal B, Fetter G, Nayler S *et al*: Association of Adipose Tissue Inflammation With Histologic Severity of Nonalcoholic Fatty Liver Disease. *Gastroenterology* 2015, 149(3):635-648.e614.

10. Fitzpatrick E, Dew TK, Quaglia A, Sherwood RA, Mitry RR, Dhawan A: Analysis of adipokine concentrations in paediatric non-alcoholic fatty liver disease. *Pediatric obesity* 2012, 7(6):471-479.

11. Haukeland JW, Damas JK, Konopski Z, Loberg EM, Haaland T, Goverud I, Torjesen PA, Birkeland K, Bjoro K, Aukrust P: Systemic inflammation in nonalcoholic fatty liver disease is characterized by elevated levels of CCL2. *Journal of Hepatology* 2006, 44(6):1167-1174.

12. Jamali R, Arj A, Razavizade M, Aarabi MH: Prediction of Nonalcoholic Fatty Liver Disease Via a Novel Panel of Serum Adipokines. *Medicine* 2016, 95(5).

13. Jamali R, Razavizade M, Arj A, Aarabi MH: Serum adipokines might predict liver histology findings in non-alcoholic fatty liver disease. *World Journal of Gastroenterology* 2016, 22(21):5096-5103.

14. Jarrar MH, Baranova A, Collantes R, Ranard B, Stepanova M, Bennett C, Fang Y, Elariny H, Goodman Z, Chandhoke V *et al*: Adipokines and cytokines in non-alcoholic fatty liver disease. *Alimentary Pharmacology and Therapeutics* 2008, 27(5):412-421.

15. Jiang L, Zhao J: CXCL16 promotes hepatocyte steatosis and fibrosis in nonalcoholic fatty liver disease via hepatocyte-stellate cell crosstalk. *Journal of Hepatology* 2018, 68:S342-S342.

16. Kobyliak N, Bosak N, Mykhalchyshyn G, Kyriienko D, Dynnyk O: Non-obese fatty liver disease in type 2 diabetes: Novel condition or similar to typical NAFLD state? *United European Gastroenterology Journal* 2017, 5(5):A627.

17. Kugelmas M, Hill DB, Vivian B, Marsano L, McClain CJ: Cytokines and NASH: a pilot study of the effects of lifestyle modification and vitamin E. *Hepatology (baltimore, md)* 2003, 38(2):413‐419.

18. Kukla M, Zwirska-Korczala K, Hartleb M, Waluga M, Chwist A, Kajor M, Ciupinska-Kajor M, Berdowska A, Wozniak-Grygiel E, Buldak R: Serum chemerin and vaspin in non-alcoholic fatty liver disease. *Scandinavian Journal of Gastroenterology* 2010, 45(2):235-242.

19. Kumar A, Sharma A, Duseja A, Das A, Dhiman RK, Chawla YK, Kohli KK, Bhansali A: Patients with Nonalcoholic Fatty Liver Disease (NAFLD) have Higher Oxidative Stress in Comparison to Chronic Viral Hepatitis. *Journal of Clinical and Experimental Hepatology* 2013, 3(1):12-18.

20. Louthan MV, Barve S, McClain CJ, Joshi-Barve S: Decreased serum adiponectin: An early event in pediatric nonalcoholic fatty liver disease. *Journal of Pediatrics* 2005, 147(6):835-838.

21. Musso G, Cassader M, Michieli FD, Paschetta E, Pinach S, Saba F, Bongiovanni D, Framarin L, Berrutti M, Leone N *et al*: MERTK rs4374383 variant predicts incident nonalcoholic fatty liver disease and diabetes: role of mononuclear cell activation and adipokine response to dietary fat. *Human molecular genetics* 2017, 26(9):1747‐1758.

22. Page S, Estep M, Afendy A, Birerdinc A, Petricoin E, Baranova A, Younossi Z: CCL-2 and Soluble Fas Ligand as Markers of Hepatic Fibrosis in Patients with Non-alcoholic Fatty Liver Disease (NAFLD). *American Journal of Gastroenterology* 2011, 106:S131-S131.

23. Parker R, Corbett C, Weston C, Armstrong M, Newsome P, Adams D: Evidence for a Role of Ccr2 in Human Non-Alcoholic Fatty Liver Disease. *Journal of Hepatology* 2015, 62:S225-S225.

24. Perito ER, Ajmera V, Bass NM, Rosenthal P, Lavine JE, Schwimmer JB, Yates KP, Diehl AM, Molleston JP, Murray KF *et al*: Association Between Cytokines and Liver Histology in Children with Nonalcoholic Fatty Liver Disease. *Hepatology Communications* 2017, 1(7):609-622.

25. Du Plessis J, Van Pelt J, Korf H, Mathieu C, Van Der Schueren B, Lannoo M, Oyen T, Topal B, Fetter G, Nayler S *et al*: Association of Adipose Tissue Inflammation with Histologic Severity of Nonalcoholic Fatty Liver Disease. *Gastroenterology* 2015, 149(3):635e614-648e614.

26. Polat Z, Uygun A, Ozel AM, Basaranoglu M, Aslan F, Gulsen M, Bagci S: Role of tweak in the pathogenesis of nonalcoholic fatty liver disease. *Gastroenterology* 2013, 144(5):S1011-S1012.

27. Polyzos SA, Kountouras J, Anastasilakis AD, Geladari EV, Mantzoros CS: Irisin in patients with nonalcoholic fatty liver disease. *Metabolism-Clinical and Experimental* 2014, 63(2):207-217.

28. Ponziani FR, Bhoori S, Castelli C, Putignani L, Rivoltini L, Del Chierico F, Sanguinetti M, Morelli D, Sterbini FP, Petito V *et al*: Hepatocellular Carcinoma Is Associated With Gut Microbiota Profile and Inflammation in Nonalcoholic Fatty Liver Disease. *Hepatology* 2019, 69(1):107-120.

29. Qi S, Xu D, Li Q, Xie N, Xia J, Huo Q, Li P, Chen Q, Huang S: Metabonomics screening of serum identifies pyroglutamate as a diagnostic biomarker for nonalcoholic steatohepatitis. *Clinica Chimica Acta* 2017, 473:89-95.

30. Serhal R, Hilal G, Boutros G, Sidaoui J, Wardi L, Ezzeddine S, Alaaeddine N: Nonalcoholic Steatohepatitis: Involvement of the Telomerase and Proinflammatory Mediators. *Biomed Research International* 2015.

31. Abu Shanab A, Scully P, Crosbie O, Buckley M, O'Mahony L, Shanahan F, Gazareen S, Murphy E, Quigley EMM: Small Intestinal Bacterial Overgrowth in Nonalcoholic Steatohepatitis: Association with Toll-Like Receptor 4 Expression and Plasma Levels of Interleukin 8. *Digestive Diseases and Sciences* 2011, 56(5):1524-1534.

32. Shoji H, Yoshio S, Mano Y, Kumagai E, Sugiyama M, Korenaga M, Arai T, Itokawa N, Atsukawa M, Aikata H *et al*: Interleukin-34 as a fibroblast-derived marker of liver fibrosis in patients with non-alcoholic fatty liver disease. *Scientific reports* 2016, 6.

33. Tang MC, Cheng L, Qiu L, Jia RG, Sun RQ, Wang XP, Hu GY, Zhao Y: Efficacy of Tiopronin in treatment of severe non-alcoholic fatty liver disease. *European Review for Medical and Pharmacological Sciences* 2014, 18(2):160-164.

34. Tarantino G, Costantini S, Finelli C, Capone F, Guerriero E, La Sala N, Gioia S, Castello G: Carotid Intima-Media Thickness Is Predicted by Combined Eotaxin Levels and Severity of Hepatic Steatosis at Ultrasonography in Obese Patients with Nonalcoholic Fatty Liver Disease. *Plos One* 2014, 9(9).

35. Torer N, Ozenirler S, Yucel A, Bukan N, Erdem O: Importance of cytokines, oxidative stress and expression of BCL-2 in the pathogenesis of non-alcoholic steatohepatitis. *Scandinavian Journal of Gastroenterology* 2007, 42(9):1095-1101.

36. Tuncer I, Özbek H, Topal C, Uygan I: The Serum Levels of IL-1β, IL-6, IL-8 and TNF-α in Nonalcoholic Fatty Liver. *Turkish Journal of Medical Sciences* 2003, 33(6):381-386.

37. Uysal S, Armutcu F, Aydogan T, Akin K, Ikizek M, Yigitoglu MR: Some inflammatory cytokine levels, iron metabolism and oxidan stress markers in subjects with nonalcoholic steatohepatitis. *Clinical Biochemistry* 2011, 44(17-18):1375-1379.

38. Wada N, Takaki A, Ikeda F, Yasunaka T, Onji M, Nouso K, Nakatsuka A, Wada J, Koike K, Miyahara K *et al*: Serum-inducible protein (IP)-10 is a disease progression-related marker for non-alcoholic fatty liver disease. *Hepatology International* 2017, 11(1):115-124.

39. Yang BB, Chen YH, Zhang C, Shi CE, Hu KF, Zhou J, Xu DX, Chen X: Low vitamin D status is associated with advanced liver fibrosis in patients with nonalcoholic fatty liver disease. *Endocrine* 2017, 55(2):582-590.

40. Ye Z, Wang S, Yang Z, He M, Zhang S, Zhang W, Wen J, Li Q, Huang Y, Wang X *et al*: Serum lipocalin-2, cathepsin S and chemerin levels and nonalcoholic fatty liver disease. *Molecular Biology Reports* 2014, 41(3):1317-1323.

41. Yilmaz Y, Yonal O, Kurt R, Alahdab YO, Eren F, Ozdogan O, Celikel CA, Imeryuz N, Kalayci C, Avsar E: Serum levels of omentin, chemerin and adipsin in patients with biopsy-proven nonalcoholic fatty liver disease. *Scandinavian Journal of Gastroenterology* 2011, 46(1):91-97.

42. Yilmaz Y, Kurt R, Gurdal A, Alahdab YO, Yonal O, Senates E, Polat N, Eren F, Imeryuz N, Oflaz H: Circulating vaspin levels and epicardial adipose tissue thickness are associated with impaired coronary flow reserve in patients with nonalcoholic fatty liver disease. *Atherosclerosis* 2011, 217(1):125-129.

43. Youness ER, Aly HF, El Nemr M: Role of apelin/monocyte chemoattractant protein-1, inflammatory, apoptotic markers in the regulation of patients with non-alcoholic fatty liver disease. *Asian Journal of Pharmaceutical and Clinical Research* 2018, 11(8):138-142.

44. Younossi ZM, Jarrar M, Nugent C, Randhawa M, Afendy M, Stepanova M, Rafiq N, Goodman Z, Chandhoke V, Baranova A: A Novel Diagnostic Biomarker Panel for Obesity-related Nonalcoholic Steatohepatitis (NASH). *Obesity Surgery* 2008, 18(11):1430-1437.

45. Zhang X, Shen J, Man K, Chu ES, Yau TO, Sung JC, Go MY, Deng J, Lu L, Wong VW *et al*: CXCL10 plays a key role as an inflammatory mediator and a non-invasive biomarker of non-alcoholic steatohepatitis. *J Hepatol* 2014, 61(6):1365-1375.

46. Zhu JZ, Zhu HT, Dai YN, Li CX, Fang ZY, Zhao DJ, Wan XY, Wang YM, Wang F, Yu CH *et al*: Serum periostin is a potential biomarker for non-alcoholic fatty liver disease: a case-control study. *Endocrine* 2016, 51(1):91-100.
